# Radiating diversification and niche conservatism jointly shape the inverse latitudinal diversity gradient of *Potentilla* L. (Rosaceae)

**DOI:** 10.1186/s12870-024-05083-8

**Published:** 2024-05-23

**Authors:** Tiantian Xue, Tao Feng, Yunfen Liang, Xudong Yang, Fei Qin, Jianghong Yu, Steven B. Janssens, Shengxiang Yu

**Affiliations:** 1 State Key Laboratory of Plant Diversity and Specialty Crops, Institute of Botany, Chinese Academy of Sciences, Beijing, 100093 China; 2 China National Botanical Garden, Beijing, 100093 China; 3 University of Chinese Academy of Sciences, Beijing, 100049 China; 4 Biosystematics Group, Wageningen University & Research, Droevendaalsesteeg 4, Wageningen, 6708 PB Gelderland the Netherlands; 5 Department of Earth System Science, Tsinghua University, Beijing, 100084 China; 6 School of Life Science, Sun Yat-Sen University, Guangzhou, 510275 China; 7 College of Forestry, Guizhou University, Guiyang, 550025 China; 8 Meise Botanic Garden, Nieuwelaan 38, Meise, BE-1860 Belgium; 9 Department of Biology, KU Leuven, Kasteelpark Arenberg 31, Leuven, BE-3001 Belgium

**Keywords:** Diversification, Evolutionary time, Inverse latitudinal diversity gradient, Niche conservatism, *Potentilla*, Species richness

## Abstract

**Background:**

The latitudinal diversity gradient (LDG), characterized by an increase in species richness from the poles to the equator, is one of the most pervasive biological patterns. However, inverse LDGs, in which species richness peaks in extratropical regions, are also found in some lineages and their causes remain unclear. Here, we test the roles of evolutionary time, diversification rates, and niche conservatism in explaining the inverse LDG of *Potentilla* (ca. 500 species). We compiled the global distributions of ~ 90% of *Potentilla* species, and reconstructed a robust phylogenetic framework based on whole-plastome sequences. Next, we analyzed the divergence time, ancestral area, diversification rate, and ancestral niche to investigate the macroevolutionary history of *Potentilla*.

**Results:**

The genus originated in the Qinghai-Tibet Plateau during the late Eocene and gradually spread to other regions of the Northern Hemisphere posterior to the late Miocene. Rapid cooling after the late Pliocene promoted the radiating diversification of *Potentilla*. The polyploidization, as well as some cold-adaptive morphological innovations, enhanced the adaptation of *Potentilla* species to the cold environment. Ancestral niche reconstruction suggests that *Potentilla* likely originated in a relatively cool environment. The species richness peaks at approximately 45 °N, a region characterized by high diversification rates, and the environmental conditions are similar to the ancestral climate niche. Evolutionary time was not significantly correlated with species richness in the latitudinal gradient.

**Conclusions:**

Our results suggest that the elevated diversification rates in middle latitude regions and the conservatism in thermal niches jointly determined the inverse LDG in *Potentilla*. This study highlights the importance of integrating evolutionary and ecological approaches to explain the diversity pattern of biological groups on a global scale.

**Supplementary Information:**

The online version contains supplementary material available at 10.1186/s12870-024-05083-8.

## Background

Understanding the drivers of the global pattern in biodiversity is a main task for ecologists and biogeographers [1]. The latitudinal diversity gradient (LDG), which refers to the decrease in species richness from the equator to the poles, stands as one of the most pervasive and widely debated patterns in biogeography [2]. However, there are also some taxonomic lineages that exhibit inverse LDGs, in which species richness reaches its zenith in extratropical regions and decreases toward the tropical regions. Such inverse LDGs have been observed in insects [3, 4], amphibians [5], birds [6, 7], mammals [6], angiosperms [8–10], and gymnosperms [11, 12]. For example, the stenammine ants species are most diverse in the 35°N to 40°N range and decrease towards the equator [3], and the Rhamnaceae species diversity is markedly higher in temperate than in tropical regions, resulting in a bimodal latitudinal diversity gradient [10]. Determining the driving forces behind these less common richness patterns is helpful in deepening our understanding of the regular LDGs.

To elucidate the evolutionary mechanisms of species richness patterns, several hypotheses have been proposed in previous studies, including the time-for-speciation hypothesis [13], the diversification rate hypothesis [14], and the niche conservatism hypothesis [15]. The time-for-speciation hypothesis suggests that regions with high species richness were colonized earlier and hence had more time to increase their diversity via ongoing speciation [13]. In contrast, the diversification rate hypothesis assumes that elevated regional species richness might arise from high diversification rates (speciation rate minus extinction rate), driven by one or more ecological variables [14, 16]. Moreover, the contemporary environment could also affect species distribution [2, 17]. The niche conservatism hypothesis states that most species tend to maintain their ancestral niches [18]. Consequently, the species richness of a group is typically high in regions with environmental conditions similar to its ancestral niche, whereas in regions with environmental conditions different from its ancestral niche, diversity tends to be low due to niche conservatism limitations on dispersal [15, 19]. Importantly, these three hypotheses are not necessarily exclusive and might act together to explain richness patterns [2, 14].

In this study, we evaluate the roles of evolutionary time, diversification rates, and niche conservatism in shaping the distribution pattern of the species-rich genus *Potentilla* L. (Rosaceae). With an approximate count of 500 species, this genus is widely distributed in Europe, temperate Asia and North America. Only a few species can also penetrate into tropical regions, a pattern that strongly suggests an inverse LDG [20–25]. Most species of this genus occur in the open habitats situated within mountainous and arctic terrains, as well as in the drylands of western North America [26]. The divergence times estimation and ancestral area reconstruction suggested that the genus originated in Asia during the Eocene, and subsequently spread to Europe and North America [26, 27]. However, the inadequate sampling and the absence of distribution data make it difficult to answer why *Potentilla* exhibits high diversity in temperate regions.

Additionally, rapid diversification may hold significance in the evolutionary history of *Potentilla*, and the information on the drivers of accelerated diversification will provide valuable insights into the underlying mechanism towards inverse LDG within the genus. Most of the extant *Potentilla* species appeared during the late Miocene to Quaternary, a period considered to have experienced rapid radiation events in the genus [26]. In particular, the Argentea clade, which is the most diverse clade of *Potentilla*, predominantly diverged after ca. 5 Ma, yet it contains more than 300 species [28, 29]. Evolutionary radiation can be triggered by abiotic factors, such as abrupt climate or tectonic changes [30–32], as well as biotic factors, such as species intrinsic properties [30, 33]. Based on the considerable proportion of polyploids in *Potentilla*, Dobeš and Paule [26] hypothesized that polyploidization might be an important element to explain its rapid speciation, yet this hypothesis has not been confirmed to date. In addition, many adaptive morphological traits within *Potentilla* have been formed in order to adapt to extreme environments. For example, *Potentilla* species in Arctic region are typically dwarf herbs covered with dense hairs to resist severe cold [20]. Unfortunately, the contributions of these abiotic and biotic factors to the high-level diversity in *Potentilla* remain unclear due to the lack of modelling diversification rates.

In order to conduct comprehensive macroevolutionary analyses, a robust phylogenetic framework is a key necessity. Although previous studies have provided a basic phylogenetic disentanglement of *Potentilla* based on a few short DNA markers [26, 28, 34–36], in-depth infrageneric relationships remain unclear, thereby hampering a proper study on spatiotemporal evolutionary trends within the genus. Recent studies have shown that plastomes can fully resolve the phylogenetic relationships between different clades and within most clades [37, 38]. Therefore, this study also utilizes plastome sequences to reconstruct a solid phylogenetic backbone with more complete sampling. We aim to: (1) estimate the divergence times and reconstruct the ancestral areas of *Potentilla*; (2) carry out diversification rate analyses, and explore the roles of abiotic and biotic factors in the accelerated diversification of *Potentilla*; (3) reconstruct its ancestral thermal niche; and (4) reveal the relationships between species richness and evolutionary time, diversification rates, deviation from ancestral niche in latitudinal gradients, and test the three hypotheses on the inverse LDG of *Potentilla*.

## Results

### Phylogenomic analysis of *Potentilla*

**Fig. 1:**
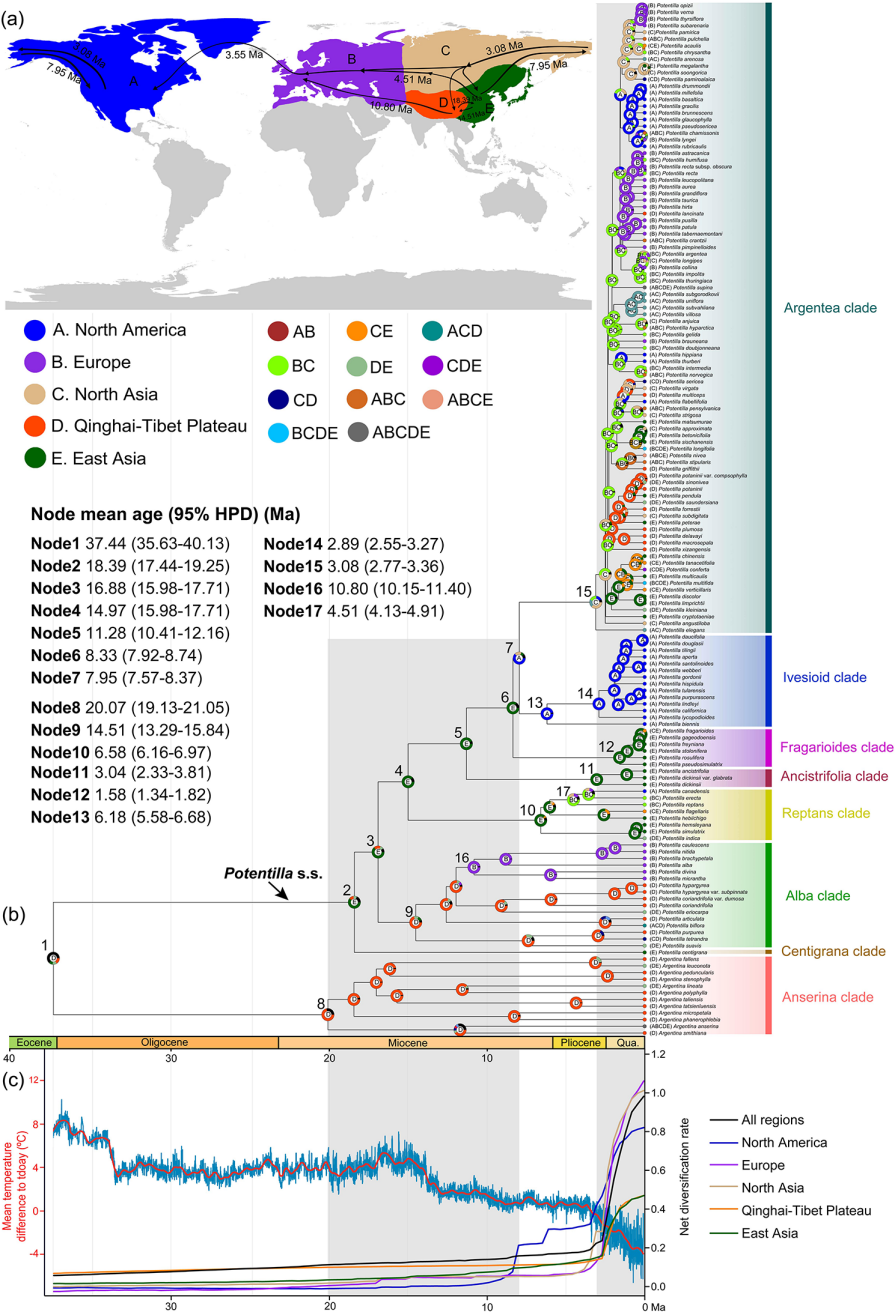
Historical biogeography of *Potentilla*. (**a**) Five biogeographic regions and potential dispersal routes of *Potentilla*. (**b**) Ancestral range estimation using BioGeoBEARS with the BAYAREALIKE + J model. Nodes 1–7 refer to the stem ages of the eight clades, nodes 8–15 refer to the crown ages of the eight clades, and nodes 16–17 refer to the dispersal events. (**c**) Temporal patterns in the net diversification rate of *Potentilla* across the globe and five biogeographic regions estimated by the empirical time-calibrated tree; red curve shows global temperature differences over the last 37.44 Ma as compared to current temperature and is modified from Westerhold et al. [88]. The map used in this study was downloaded from DIVA-GIS (http://www.diva-gis.org/Data). Qua. = Quaternary

Based on whole plastome sequences, *Potentilla* is strongly supported as monophyletic (Fig. 1; Fig. S1). A total of eight clades were identified with high bootstrap values (BS = 100% in Fig. S1). The Anserina clade is the earliest-diverging lineage and forms a sister group to *Potentilla* s.s. Within *Potentilla* s.s., the Centigrana clade is the basalmost, followed by Alba, Reptans, Ancistrifolia, Fragarioides, Ivesioid, and Argentea. Except for the Argentea clade, the internal relationships were fully or basally resolved within other clades. However, for the Argentea, which is the most diverse clade in *Potentilla*, the internal relationships were less clear. Along with three early derived species, six subclades could be retrieved in the Argentea clade, but all subclades only received low support values.

### Divergence time and ancestral range estimation

The divergence times estimated by the penalized likelihood method in treePL indicated that the stem age of *Potentilla* was 47.64 Ma (95% highest posterior density [HPD]: 45.85–50.65 Ma, Fig. S2). *Potentilla* split into the Anserina clade and *Potentilla* s.s. at 37.44 Ma (95% HPD: 35.63–40.13 Ma; node 1) (Fig. 1B). The Anserina clade began to diversify at 20.07 Ma (95% HPD: 19.13–21.05; node 8). Within *Potentilla* s.s., the stem node ages of the extant clades ranged from 18.39 to 7.95 Ma (nodes 2–7). However, except for the Centigrana and Alba clades, the other clades began to diversify after 7 Ma (nodes 10–15). All species of the Fragarioides clade (node 12), and the Argentea clade (node 15), as well as most species of the Ivesioid clade (node 14) originated after 3.08 Ma. Among the 154 taxa sampled in this study, 86% of them originated after ca. 3 Ma, and 52% originated after 1 Ma (Table. S10). For the 302 species without plastome, their simulated ages were presented in Fig. S3 and Table S11.

Ancestral area reconstruction showed that the genus *Potentilla* originated on the Qinghai-Tibet Plateau during the late Eocene (Fig. 1B, node 1). The Anserina clade was mainly distributed in the Qinghai-Tibet Plateau. Within *Potentilla* s.s., several dispersal events could be inferred (Fig. 1A, B). The early two dispersal events occurred between the Qinghai-Tibet Plateau and East Asia (nodes 2 and 9). Subsequently, three dispersal events were inferred from Asia to Europe and North America (nodes 7, 16, and 17). Among these, the first dispersal event occurred in the Alba clade, of which the most recent common ancestor originated on the Qinghai-Tibet Plateau and from where members of the clade subsequently dispersed into Europe during the middle Miocene (node 16). A second dispersal event occurred in the Reptans clade, of which the most recent common ancestor originated in East Asia and from where members of the clade subsequently dispersed towards Europe, North Asia, and North America during the Pliocene (node 17). A third dispersal event took place in the late Miocene (node 7) from East Asia to North America, thereby forming the Ivesioid clade being confined to eastern North America. Finally, *Potentilla* recolonized North Asia in the late Miocene from North America (node 15) after which some of its member dispersed again to the other four biogeographic regions. In accordance with the ancestral area reconstruction, multiple dispersal events between North America and Eurasia occurred in the Argentea clade. In addition, during a very short period (ca. 3 Ma), each biogeographic region is characterized by a rapid radiation, thereby forming the highly diverse Argentea clade (Fig. 1B).

### Diversification rate analyses

**Fig. 2:**
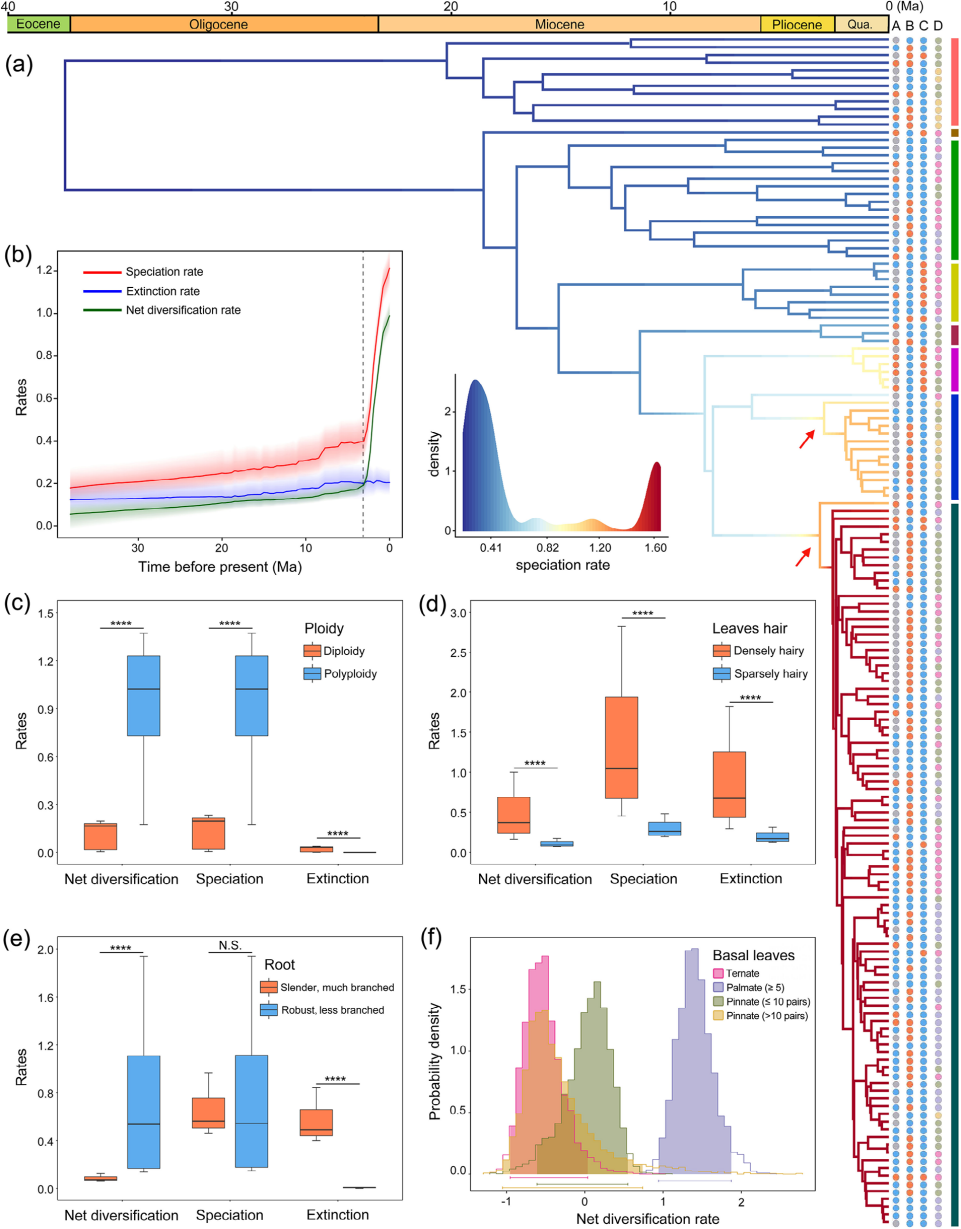
Diversification rate analyses of *Potentilla*. (**a**) Phylorate plot showing the speciation rates along each branch. Arrows, shifts in speciation rates. The four traits used for trait-dependent analyses (A–D) are plotted at right—A: ploidy; B: leaves hair; C: root; D: basal leaves. The colors of the circles in A–D correspond to the boxplots c–f. (**b**) Speciation, extinction, and net diversification rates over time according to BAMM analysis. (**c–e**) Binary trait-dependent diversification inferred by HiSSE analyses. (**f**) Multistate trait-dependent diversification estimated by MuSSE analysis. Asterisks in (**c**-**e**) indicate significant differences according to *t* tests. ****P < 0.0001; N.S., not significant

The phylorate plot of the speciation rate revealed two shifts in diversification within *Potentilla* having high posterior probability (Fig. 2A; Fig. S4): one in the Ivesioid clade and the other in the Argentea clade. Diversification rates were low in early diverging clades, such as Anserina, Alba, and Reptans, but high in more recently diverged clades such as Fragarioides, Ivesioid, and Argentea. The Argentea clade showed rapid diversification after roughly 3 Ma with a diversification rate of 1.6 species per million years (Fig. 2A). In addition, the rate-through-time plots showed that the speciation and net diversification rates of *Potentilla* increased slowly prior to the Pliocene and then significantly accelerated, while extinction rates remained almost always constant (Fig. 2B). For the net diversification rates of *Potentilla* in the five biogeographical regions, whether based on the empirical or the simulated time-calibrated tree, their temporal trends are almost synchronized (Fig. 1C; Fig. S5). The early diversification within the *Potentilla* is shown to be rather slow, mainly occurring on the Qinghai-Tibet Plateau. Then, diversification rates throughout the whole *Potentilla* drastically increased in all biogeographical regions nearly simultaneously after the late Pliocene, a period when global temperatures were strongly decreasing (Fig. 1C). Interestingly, diversification rates in regions at lower latitudes (Qinghai-Tibet Plateau and East Asia) became gradually exceeded by those in regions at higher latitudes (North America, Europe, and North Asia) (Fig. 1C; Fig. S5).

The paleotemperature-dependent analysis employed in RPANDA v.1.9 show that the linear speciation without extinction with temperature dependence is the best model (AICc = 637.606) (Table S5), and the diversification rate increased with the decrease of temperature (α=-0.055), which was consistent with the results of BAMM analysis (Fig. 1C). For the trait-dependent analyses, the best model for the three binary traits was the full HiSSE model, which had unique speciation, extinction, and transition rates between the two observed character states and hidden states (AICc = 622.863, 746.362, and 622.863, respectively; Table S8). Polyploid species, as well as species with densely hairy leaves and robust root with few branches have higher net diversification rates (Fig. 2C–E). For the MuSSE analysis, the full model was selected as best best-fitting model, i.e., it had the lowest AICc (AICc = 930.716) (Table S9). Species with palmate shaped (≥ 5) basal leaves had higher net diversification rates than other basal leaf types (Fig. 2F). Interestingly, this palmate leaf type is generally present among the species of the Argentea clade.

### Distribution pattern of species richness, evolutionary time, diversification rate, and deviation from ancestral niche

**Fig. 3:**
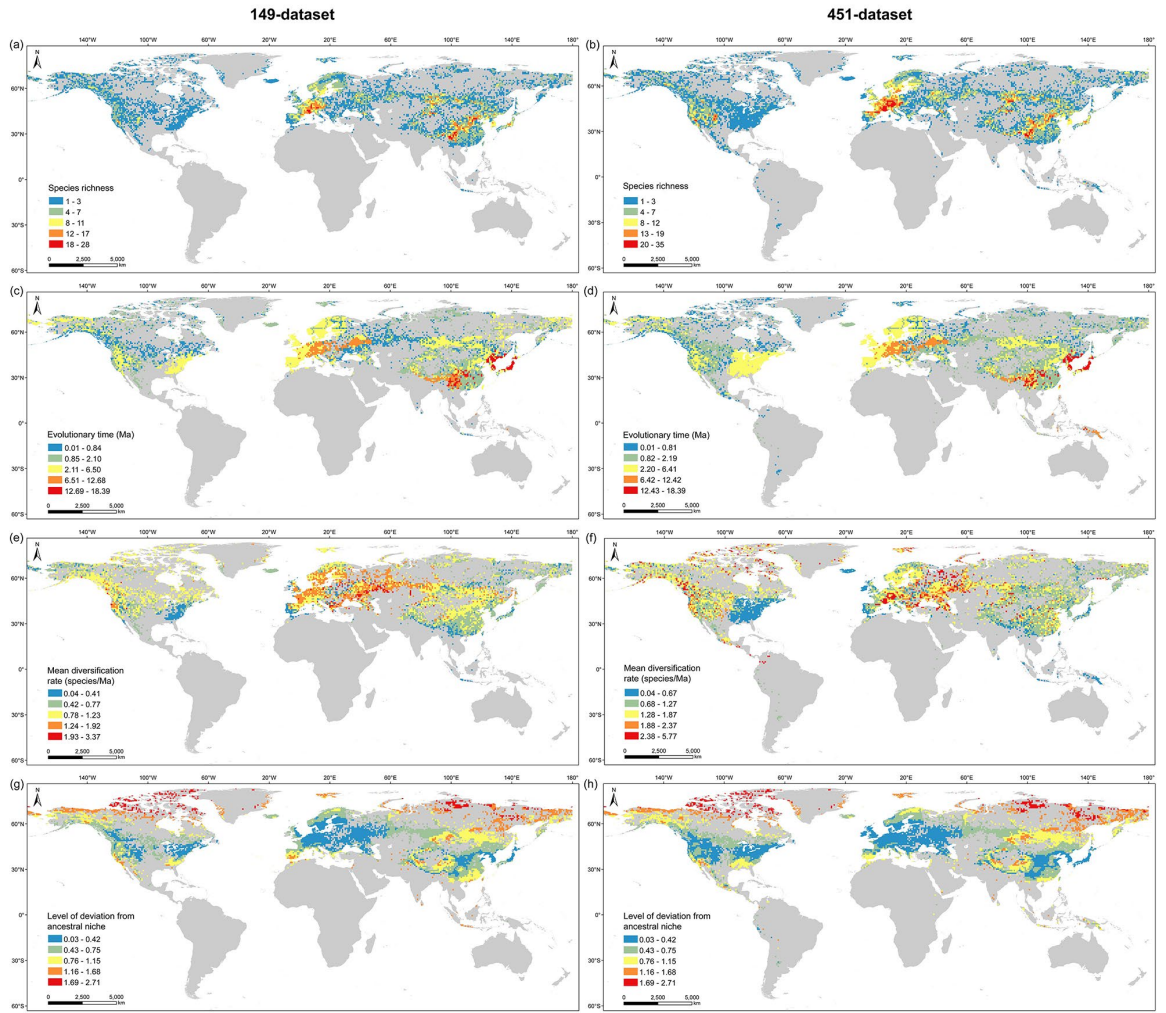
Distribution of species richness (**a**, **b**), evolutionary time (**c**, **d**), mean diversification rates (**e**, **f**), and level of deviation from ancestral climate (**g**, **h**) of *Potentilla* calculated based on the 149-dataset and 451-dataset, respectively. The resolution of grid cells was 100 km × 100 km

**Fig. 4:**
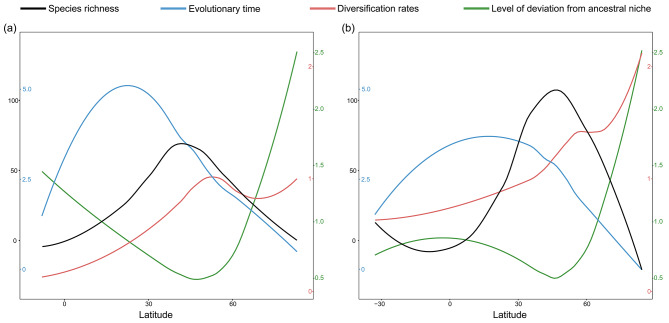
Latitudinal trends in species richness, evolutionary time, diversification rates, and level of deviation from ancestral niche calculated based on the 149-dataset (**a**), and the 451-dataset (**b**)

Distribution pattern analyses showed that *Potentilla* mainly occurred in the northern temperate zone, with only a few species extending into tropical regions (Fig. 3A, B). Species richness of *Potentilla* peaked around 45 °N, and decreased toward both high and low latitudes (Fig. 4). Most *Potentilla* species are confined to montane areas at middle latitudes, such as the Alpine Mountains in Europe, the Tianshan and Altai Mountains in North Asia, the Hengduan and Qilian Mountains within Qinghai-Tibet Plateau, the Qinling and Taihang Mountains in East Asia, and the Nevada Mountains in western North America (Fig. 3A, B). The distribution pattern of species richness based on the 149-dataset resembled that of the 451-dataset, except for the latter exhibiting higher *Potentilla* richness in western North America. Sampling bias analysis showed that the waterbodies have more obvious effect on sampling intensity than the airports, cities and roads (Fig. S6). Northeast and Central Asia showed a low number of collection records (Fig. S7).

Regarding the distribution pattern of evolutionary time, *Potentilla* species in Qinghai-Tibet Plateau and East Asia are much older than those in Europe, North America and North Asia (Fig. 3C, D). In the latitudinal gradient, whether based on empirical or simulated datasets, the evolutionary time at ca. 20 °N is significantly higher than that at other latitudes (Fig. 4). These results are consistent with the dispersal histories of *Potentilla* as revealed by the ancestral area reconstruction (Fig. 1).

The mean diversification rates in Europe, western North Asia and western North America were significantly higher than in all other regions, corroborating the higher net diversification rates estimated by BAMM analyses in these regions (Fig. 1C; Fig. S5). In the latitudinal gradient, diversification rates generally increase with increasing latitude, punctuated by a slight trough around 65 °N (Fig. 4). However, at high latitudes (from 60 °N to 80 °N), the diversification rates calculated based on the 451-dataset were noticeably higher than those based on the 149-dataset. This is possibly because the 451-dataset includes more species from North Asia, North America, and Europe, most of which appeared explosively after 3 Ma (Fig. S3; Table S3).

The ancestral thermal niches of *Potentilla* reconstructed for MAT, MTWM, and MTDQ were 6.66 ℃, 23.68 ℃, and 1.22 ℃, respectively (Fig. S8), implying that *Potentilla* likely originated in a relatively cool environment. Notably, ancestral temperature inferred from the simulated time-calibrated tree was slightly higher than that from the empirical time-calibrated tree (Figs. S8 and S9). This could be attributed to the presence of some inserted Anserina species, which are native to low-latitude regions, particularly New Guinea (Table S2), and consequently elevated the estimated temperature of the root node. The level of deviation from ancestral niche, as well as the ΔMAT, ΔMTWM, and ΔMTDQ, showed lower values at the diversity hotspots of *Potentilla*, such as Europe, western North America, East Asia, and the surrounding area of Qinghai-Tibet Plateau (Fig. 3G, H; Fig. S10). Both datasets showed that the contemporary environment at ca. 45 °N is most similar to the ancestral niche of *Potentilla*, with increased deviation towards to lower and higher latitudes (Fig. 4).

## Discussion

### Phylogeny of *Potentilla*

We present a robust phylogenetic framework for *Potentilla* based on whole plastome data and a global sampling (Fig. S1). The infrageneric relationships among *Potentilla* were generally congruent with prior studies, except for some minor differences [26, 28, 35, 37, 38]. Based on three plastid DNA makers, Dobeš and Paule [26] placed Argentea as sister to Fragarioides, and their relationships with Ivesioid clade are unclear. Our plastome-based phylogeny supported Ivesioid as a sister clade of Argentea with high support. For the Alba clade, Feng et al. [28] indicated that the Himalayan clade (including *P. purpurea*, *P. tetrandra*, and *P. suavis*) formed a sister group to the remaining species of *Potentilla* s.s. in plastid gene tree. However, we resolved the Himalayan clade in the Alba clade as an early derived lineage, which was supported by other plastid-based studies [26, 35–38]. In general, compared with previous studies, most clades had good sampling representativeness with the internal relationships being well resolved, thereby facilitating further biogeographic, evolutionary, and ecological studies.

### Biogeographic history of *Potentilla* in the Northern Hemisphere

The current divergence time estimation suggested a crown group age of 37.44 Ma (95% HPD: 35.63–40.13) for *Potentilla*. This is in agreement with the results of Dobeš and Paule [26] (95% HPD: 34.3–45.2 Ma). Additionally, the divergence times of major nodes within the genus *Potentilla* are in congruence with the above-mentioned study. Our ancestral area reconstruction analysis indicated that the most recent common ancestor of *Potentilla* originated in the Qinghai-Tibet Plateau around the Eocene-Oligocene boundary (Fig. 1B, node 1), which was more accurate than the previously suggested Asian origin [26]. Then, the *Potentilla* diverged into two groups: the Anserina clade and *Potentilla* s.s. The former was mainly distributed in high-altitude areas of the Qinghai-Tibet Plateau, especially in the Hengduan Mts (Fig. S11H). The Anserina clade started to diverge from the early Miocene to the Quaternary, which corresponds with the uplift time of the southeastern Qinghai-Tibet Plateau [39]. As such, the uplift of the Plateau might potentially have triggered the in situ diversification of the Anserina clade, similar to what happened in *Rhodiola* [40] and *Saxifraga* [41].

From the middle Miocene to the early Pliocene, there were three out-of-Asia dispersal events. The first dispersal event occurred from the Qinghai-Tibet Plateau towards Europe, and involved the Alba clade (Fig. 1B, node 16). Except for *P. biflora*, which was also found in North America [24], the other species of the Alba clade are confined to high mountains of Eurasia, particularly the Himalayas and the Alps (Fig. S11F). Therefore, we hypothesize that the Alba clade spread over the Himalayas towards the Caucasus (*P*. *divina* and *P*. *brachypetala*) and the Alps (*P*. *nitida*, *P*. *caulescens*, and *P*. *alba*). Although the Himalayas Mts have uplifted to a considerable elevation during this period [42], this dispersal path was still feasible given that most species of this clade were distributed at high altitudes (Fig. S12). Similar dispersal routes were reported for *Gentiana* [43]. The other two out-of-Asia dispersal events took place in the Reptans clade (node 17) during the early Pliocene and in the Ivesioid-Argentea clade (node 7) during the late Miocene. We postulate that the Reptans clade spread from East Asia towards North Asia and Europe (*P. reptans* and *P. erecta*), and then further migrated towards eastern North America (*P. canadensis*) via the North Atlantic land bridge (NALB). Although it was previously assumed that the NALB became inexistent after the middle Miocene [44], recent studies suggested that a discontinuous land bridge could still have acted as a corridor for plant migration until the onset of the Pliocene [45, 46]. For the latter, given that the Bering Land Bridge (BLB) served as a corridor for plant migrations from the late Cretaceous to late Neogene [46], we inferred that the Ivesioid-Argentea clade spread from East Asia to North America via the BLB. Subsequently, the Ivesioid-Argentea clade split into two clades: the Ivesioid clade, which diversified in west North America, and the Argentea clade, which returned to North Asia. The Ivesioid clade, except *P. biennis*, is characterized by many drought-tolerant traits enabling it to adapt to extremely xeric conditions, indicating that aridification in western North America triggered diversification of the Ivesioid clade [47]. The species-rich clade, Argentea, probably spread from North America to North Asia via the BLB and underwent multiple transcontinental migrations and evolutionary radiation in the Northern Hemisphere over a very short period beginning in the late Pliocene.

### Rapid diversification of *Potentilla* from the late Pliocene to Quaternary

The diversification rates in the genus *Potentilla* accelerated during the late Pliocene to Quaternary, when the global temperature markedly dropped (Fig. 1C). Furthermore, paleotemperature and trait-dependent analyses showed that a decrease in temperature, polyploidization, and some adaptive traits may have promoted an increased diversification in *Potentilla* (Fig. 2). Abrupt environmental changes, such as cooling, may increase the frequency of ameiotic gamete formation, thereby promoting polyploid formation [48]. Compared with diploid taxa, polyploids tend to be more adaptable to extreme environments, which has been suggested to be important for the adaptation to lower temperatures [49]. In China, the polyploid frequency of angiosperms increased from low to high latitudes [50]. In this study, polyploid species in *Potentilla* are characterized by higher diversification rates than for diploids. In addition, polyploid *Potentilla’s* diverged recently, and are distributed in colder environments (Fig. S13). The Argentea clade contains a large number of polyploids and had the highest species richness diversification rates (Fig. 2). All species of this clade rapidly diverged after the late Pliocene, with many of them adapted to extremely low temperatures (Figs. S8 and S9). Moreover, some morphological traits in *Potentilla*, such as densely hairy leaves and robust root were considered to help plants protect against cold conditions [51–53]. These adaptive traits are typically observed in the cold-adapted Argentea clade, as well as in the high-altitude adapted Alba and Anserina clades. By contrast, species of the Reptans and Fragarioides clades prefer warmth and occur in lower altitudes, and typically have sparsely hairy leaves and slender root (Fig. 2; Figs. S8 and S9). Therefore, we postulate that the rapid cooling after the late Pliocene can be regarded as an important driver of polyploidization in *Potentilla*. The polyploidization, accompanied by innovations of adaptive traits, including densely hairy leaves and robust root with few branches enhanced the adaptation of *Potentilla* species to the cold environment. Although paleobotanists believe that Quaternary speciation was rare due to climatic oscillations [54], a recent review of molecular phylogenetic studies found that plant speciation and radiation were ubiquitous during the Quaternary [55]. Our findings provide an example of how plants adapted to low temperatures during the Quaternary and underwent rapid radiation in high latitude regions.

### Evaluation of alternative LDG hypotheses

The LDG can be explained by latitudinal variations in environmental factors, diversification rates, and the time for species accumulation [2]. We found that *Potentilla* has a strong inverse diversity gradient, with most species inhabiting the northern temperate zone between 35 and 55°N latitude. In contrast, evolutionary time generally decreased from low (ca. 20 °N) to high latitudes, possibly because the oldest clade of *Potentilla*, Anserina, was mainly distributed in the Qinghai-Tibet Plateau. Ancestral area reconstruction showed that *Potentilla* originated in the Qinghai-Tibet Plateau during the late Eocene (ca. 37 Ma) and gradually spread to other regions of the Northern Hemisphere after the late Miocene (ca. 10 Ma). Although being colonized later, regions at higher latitudes (North America, Europe, and North Asia) have accumulated more *Potentilla* species compared to regions at lower latitudes (Qinghai-Tibet Plateau and East Asia) (Table S3). Consequently, the time-for-speciation hypothesis is not applicable to the inverse LDG of *Potentilla*.

The rapid radiation after the late Pliocene contributed ∼90% of extant *Potentilla* diversity, in sharp contrast to the relatively ancient origin of *Potentilla*, indicating that rapid radiation contributed to the overall diversity in *Potentilla*. Despite the differences between the 149- and 451-datasets, it is undeniable that diversification rates in the North America, Europe, and North Asia are significantly higher than those in the Qinghai-Tibet Plateau and East Asia (Figs. 1C and 3E-F, and 4). This trend aligns with the higher *Potentilla* richness observed in the former three regions (Table S3). Thus, the high diversity of *Potentilla* in middle latitudinal regions of the Northern Hemisphere likely results from the elevated diversification rates in these regions.

Ancestral niche reconstructions indicate that most of the *Potentilla* species have existed in temperate environmental conditions since their origin (Figs. S8 and S9), suggesting a strong tendency to remain in their ancestral climatic niche. With increased deviation from the ancestral climate, the diversity of overall *Potentilla* species decreased (Fig. 4). Previous studies have also shown that plants originating in temperate climates are less adaptable to warmer conditions but more cold-tolerant than those originating in tropical climates [56]. Therefore, we speculate that the conservatism of *Potentilla* species in cool environments restricted their distribution to the northern temperate zone, and imposed climatic constraints on dispersal into tropical regions. Meanwhile, for species that are conservative in their climatic niche, altering their existing distribution range may prove challenging [8]. Given that many *Potentilla* plants are common herbal medicines used in folk medicine [57], human activities such as overharvesting may cause some species to become threatened or even extinct. So, it is necessary to carry out in situ or ex situ conservation for these species.

In conclusion, our results suggest that the radiating diversification and niche conservatism jointly shaped the inverse LDG of *Potentilla*. It is pervasive for species to retain the climatical preferences of their ancestors [18, 19]. An increasing number of studies have found that niche conservatism play a major role in shaping various distribution patterns, including LDGs [15, 58], inverse LDGs [3, 5, 8, 10], and others [59, 60]. However, the influence of evolutionary time and diversification rates on driving species distribution patterns varies greatly across lineages, regardless of whether these distribution patterns conform to LDGs or inverse LDGs [3, 6, 9, 10, 12]. Generally, the lineages with ancient origins tend to favor the time-for-speciation hypothesis, such as Cycadaceae (stem age: ca. 260 Ma) [12], *Pinus* (ca.155 Ma) [11], stenammine ants (ca. 90 Ma) [3], Caudata (ca. 198 Ma) [6], while lineages with relatively young origins usually support the diversification rate hypothesis, such as Zygophyllaceace (ca. 69 Ma) [60], Rhododendron (ca. 68 Ma) [17], Anseriformes (ca. 71 Ma) [6], Procellariiformes (ca. 61 Ma) [6]. This may be because the effect of the diversification rate on the distribution pattern of a taxon can manifest over a short period, while the effect of evolutionary time is relatively delayed [2]. Although a consensus on the mechanisms behind LDGs or inverse LDGs has not yet been reached, it is evident that the species distribution patterns are driven by multiple factors, and different hypotheses are not necessarily exclusive [2, 6, 60], Therefore, it is crucial to integrate ecological and evolutionary approaches to investigate the mechanisms underlying the global patterns of biological groups.

## Conclusion

Through interdisciplinary evidence from evolutionary ecology, we have illuminated the underlying mechanism driving the inverse LDG of *Potentilla*. Our results indicate that *Potentilla* originated in the Qinghai-Tibet Plateau during the late Eocene and then spread to other regions of the Northern Hemisphere after the late Miocene. It is postulated that the decreased temperature after the late Pliocene significantly accelerated the diversification rates of *Potentilla*. The reconstruction of thermal niche suggests that the ancestor of *Potentilla* preferred relatively cool temperatures, and the regions where environmental conditions similar to its ancestral niche usually have high species diversity. The middle latitude regions, been colonized more recently but harbored higher diversity, suggest that the time-for-speciation effect may not fully explain the inverse LDG of *Potentilla*. While the diversification rates hypotheses and niche conservatism hypotheses jointly contributed to the inverse LDG of *Potentilla*. The integrative framework incorporating both ecological and evolutionary approaches demonstrated here can lead to a better understanding of the factors shaping global patterns of plant diversity.

Although this study reconstructed a time-calibrated phylogeny for *Potentilla* with most complete sampling to date, our sampling for species in North America, Europe, and North Asia remains limited. Most of these unsampled species are regional endemic, posing a huge challenge for our sampling. Future studies should increase the sampling of *Potentilla* species from middle-high latitude regions. Furthermore, field surveys in Northeast and Central Asia should be intensified to compile a more complete geographical distribution database for *Potentilla*.

## Materials and methods

### Taxon sampling, genome-skimming sequencing, and plastome assembly

In this study, we adopted a broad circumscription for *Potentilla*, as in prior reports [26, 35, 61], including the genera *Horkelia*, *Horkeliella*, and *Ivesia* of the Ivesioid clade, and *Argentina* of the Anserina clade. A total of 184 plastomes were analyzed. Among these, 11 were newly sequenced and 8 were assembled from genome skimming data downloaded from the GenBank SRA database; the others were downloaded from GenBank (Table S1). Samples investigated comprised 179 species of Rosaceae, of which 149 are ingroup species and 30 are outgroup species. Our sampling covered all eight proposed clades of *Potentilla* [35, 37]. For the most species-rich Argentea clade, we sampled 94 taxa, comprising 92 species, 1 variety, and 1 subspecies. The newly generated plastomes have been uploaded to GenBank. The formal identification of these samples was undertaken by Tiantian Xue and Shengxiang Yu (Institute of Botany, Chinese Academy of Sciences). All voucher specimens were deposited in the herbarium of the Institute of Botany, Chinese Academy of Sciences (PE). Taxon names, voucher information, and GenBank accession numbers are listed in Table S1. To avoid under sampling, we also included species not covered in phylogeny that met both of the following criteria: (1) species name is accepted in the Plant of the World Online (POWO, https://powo.science.kew.org/), (2) species can be determined which phylogenetic lineage it belongs to by previous molecular analyses, *Flora*, type specimens or other literatures, and (3) occurrence points can be obtained from Global Biodiversity Information Facility (GBIF) or Chinese Virtual Herbarium (CVH). Finally, a total of 302 species were selected for supplemental analyses (Table S2). The sample fraction was calculated at global and region scales (Table S3).

We extracted total genomic DNA from silica-dried leaf materials or herbarium specimens using a modified CTAB method [62]. All of the samples were sequenced at Novogene Corporation (Beijing, China), which generated sequencing libraries using the NEB Next® Ultra™ DNA Library Prep Kit for Illumina following the manufacturer’s recommendations. The DNA libraries were sequenced on the Illumina HiSeq X-Ten platform and 150-bp paired-end reads were generated. We obtained the genome skimming data for each sample (ca. 4 Gb). Before assembling the plastomes, the raw sequence reads were subjected to quality control by Novogene Corporation using fastq software [63] to obtain high-quality clean reads. *De novo* plastome assembly was performed in the GetOrganelle [64] pipeline with default parameters.

### Phylogenetic analysis and divergence time estimation

Whole plastomes with one inverted repeat region excluded were used for phylogenetic reconstruction and molecular clock analysis. We first aligned the plastomes using MAFFT v. 7.490 [65] with the auto strategy, and poorly aligned regions were removed by trimAL v. 1.4 [66] using the command ‘-automated1.’ Subsequently, we performed a maximum-likelihood (ML) analysis in RAxML-NG (RAxML Next Generation) [67], with 1,000 rapid bootstrap (BS) replicates. The best-fit substitution model (GTR + F + I + G4) was selected by ModelFinder [68] according to the corrected Akaike information criterion (AICc).

Divergence times were estimated using the penalized likelihood method in treePL, which is suitable for dealing with large datasets with hundreds of species [69]. The ML tree generated from RAxML-NG as the input tree. First, we identified the best optimization parameters using the prime command. Next, we carried out a cross-validation analysis with test values of 10^− 20^ to 10^20^ to determine the best smoothing value, which was 10^− 11^. Finally, 1,000 ML bootstrap trees with branch lengths generated from RAxML-NG were used to estimate the age of each internal node, and the 95% HPD intervals were calculated using TreeAnnotator v. 2.6.7 in the BEAST package [70]. Although a number of fossils have been reported for *Potentilla*, most of which lacked key identified traits, and their reliability needs to be further evaluated (http://ifpni.org/genus.htm?id=CFE35DDB-0C1E-4248-B033-1D88273E7B95, accessed 15 July 2022). Therefore, two calibration points were utilized within outgroups. The crown age of Rosaceae was constrained with maximum and minimum ages between 96.36 and 94.46 Ma based on the estimate of Zhang et al. [71]. The oldest fossil record of the genus *Rosa* (*Rosa germerensis*, 55.8–48.6 Ma) was used to calibrate the crown age of the *Rosa*-Potentilleae clade (cf. Palaeobiology database https://paleobiodb.org) [72]. To assess the impact of missing species on subsequent analyses, the 302 *Potentilla* species without plastomes were added randomly to their respective clades in the empirical time-calibrated tree, using the *add.species* function in the R package phytools v. 0.7-00 [73]. This approach has been commonly employed in previous macroevolutionary studies of highly diverse groups [74–76]. Ultimately, two time-calibrated trees were generated: an empirical tree comprising 149 species with plastomes, and a simulated tree comprising 451 species.

### Ancestral area reconstruction

To trace the biogeographic history of *Potentilla*, ancestral area reconstruction was conducted using BioGeoBEARS [77] implemented in RASP v. 4.3 [78]. The time-calibrated phylogenetic tree was obtained from treePL, and outgroups were pruned. According to the endemism centers and extant distribution patterns of *Potentilla*, five geographic regions were defined: North America, Europe, North Asia, Qinghai-Tibet Plateau, and East Asia. Six models were tested in RASP v. 4.3: the dispersal-extinction-cladogenesis (DEC), the likelihood version of dispersal-vicariance (DIVALIKE), the BayArea likelihood version of the range evolution model (BAYAREA), and the + J versions of these models, which include founder-event speciation. The BAYAREALIKE + J model with the highest log-likelihood (LnL) value and low AICc value (LnL = -406.3, AICc = 818.7, Table S4) was selected as the best-fit model.

### Diversification rate analyses

The diversification rate of *Potentilla* was estimated using Bayesian Analysis of Macroevolutionary Mixtures (BAMM) (v.2.5) [79]. Initially, the prior values were selected using the *setBAMMpriors* function of BAMMtools in R v.4.2.2 [80]. The parameter *globalSamplingFraction* was set to be 0.3 following the sampling coverage in the current phylogeny to account for incomplete sampling, and the Markov chain Monte Carlo (MCMC) was run for 10 million generations and sampled every 5,000 generations. The initial 10% of samples of the MCMC run were discarded as burn-in, and the remaining data were assessed for convergence to ensure that the ESS values were > 200. We used the *addBAMMshifts* function to infer possible shifts of speciation across the phylogeny. Subsequently, we used the *PlotRateThroughTime* function in BAMMtools to plot the net diversification rates through time for the genera at the global scale and in the five biogeographic regions. Given that the uneven sampling fraction may introduce bias into the diversification rate estimation in these five biogeographical regions. We also plotted the net diversification rates through time for the five biogeographic regions based on the simulated time-calibrated tree, with *globalSamplingFraction* set to 0.9.

To explore the effects of abiotic and biotic factors on the diversification rates of *Potentilla*, we carried out paleoenvironment- and trait-dependent analyses, respectively. We used RPANDA v. 1.9 [81] to evaluate seven paleotemperature-dependent birth–death models (Table S5). The relationships of speciation and extinction rates with paleotemperature were assumed to be constant, linear, or exponential. The global paleotemperature data set was obtained from Sun et al. [82]. We compared the likelihood values, and the model with the smallest AICc was considered the best diversification model.

For the trait-dependent analyses, four traits were selected, including three binary traits (diploidy vs. polyploidy; leaves densely hairy vs. sparsely hairy; and roots robust with less branching vs. slender with much branching) and one multi-state trait (basal leaves ternate vs. palmate ≥ 5 vs. pinnate ≤ 10 pairs vs. pinnate > 10 pairs) (Table S6). Chromosome ploidy data of *Potentilla* were extracted from the Chromosome Counts Database (http://ccdb.tau.ac.il/, accessed 7 July 2022) and Index to Plant Chromosome Numbers (http://legacy.tropicos.org/Project/IPCN, accessed 7 July 2022). The ploidy of a species was divided into diploidy and polyploidy. For species with multiple cytotypes, we scored ploidy as the most frequently reported ploidy level. The three morphological traits were selected and coded based on the *Flora of China* [23], *Flora of North America* [24, 25], *Flora Europaea* [22], and *Flora URSS* [21] and the herbarium specimens in JSTOR (https://plants.jstor.org/) and the Chinese Virtual Herbarium (http://www.cvh.ac.cn/). The effects of the three binary states on diversification rates were estimated using the hidden state speciation and extinction (HiSSE) model, which enables testing of hypotheses related to the effects of observed traits and incorporates unmeasured factors [83]. Twenty-five models were tested in the R package HISSE v. 1.9.10 and the model with the lowest AICc was selected as the best-fit model (Tables S7 and S8). The extinction, speciation, and net diversification rates of each state were calculated. For the basal leaf type, multi-state speciation and extinction (MuSSE) analysis was performed in DIVERSITREE v. 0.9.10 [84]. Four models were tested: a null model with fully constrained variables; a full model allowing all variables to change independently; a model constraining the extinction rate (µ) to be equal and allowing the speciation rate (λ) to vary (free λ); and a model constraining the λ to be equal and allowing µ to vary (free µ) (Table S9). Next, the net diversification rate for each state was obtained by a Bayesian approach (MCMC analysis) with an exponential prior with 5000 generations.

### Ancestral niche reconstruction

In order to estimate the ancestral niche of *Potentilla*, we first compiled a database including the occurrence data and climate data, and conducted principal component analysis (PCA) to determine the main driving climatic factors for the distribution pattern of *Potentilla.* The occurrence data were obtained from CVH (http://www.cvh.ac.cn/) and GBIF (downloaded on 01 January 2021, from 10.15468/dl.fwp545). Then, we removed records meeting one of the following criteria: (1) without longitude or latitude; (2) equal latitude and longitude; (3) zero and/or integer latitude and longitude; (4) coordinates falling within sea; (5) coordinates were outside the species’ native range recorded in POWO. A total of 214,231 distribution records for 451 *Potentilla* species passed the above filtering criteria. For each record, we extracted the values of 19 bioclimatic variables from WorldClim (http://worldclim.org) using ArcGIS 10.6. Highly correlated variables (absolute Pearson’s correlation coefficient *r* ≥ 0.75) were removed to reduce collinearity. The remaining eight bioclimatic variables were: mean annual temperature (MAT), isothermality, max temperature of warmest month (MTWM), mean temperature of wettest quarter (MTWQ), mean temperature of driest quarter (MTDQ), annual precipitation (AP), precipitation seasonality (PS), precipitation of coldest quarter (PCQ). Then, a PCA was performed using the R packages FactoMineR v.2.6 [85] and factoextra v.1.0.7 [86]. The first two principal components (PCs) of bioclimatic variables explained 70.2% of the total climate variation in *Potentilla*, and the MAT, MTWM, and MTDQ had the largest contribution to the first two PCs (Fig. S14). Therefore, we reconstructed the ancestral states of these three bioclimatic variables using the *fastAnc* function in phytools. Mean values of the MAT, MTWM, and MTDQ for each species were used in the reconstruction.

### **Distribution pattern of species richness, evolutionary time, diversification rate, and** deviation **from ancestral climate**

Considering that the *Potentilla* comprises at least 500 species and it is difficult to obtain a complete sampling at the plastome level, here we evaluated the effect of missing species on macroevolutionary analyses. Two datasets were employed to investigate the distribution pattern of *Potentilla*: one dataset comprised solely of the 149 species with genomic data (referred to as the “149-dataset”), while the other dataset encompassed species with and without genomic data (referred to as the “451-dataset”). To mitigate the effect of area on diversity estimation and potential sampling incompleteness, we divided the map of world into 31,203 grid cells with a 100 km × 100 km (1 × 10^4^ km^2^) resolution utilizing ArcGIS 10.6. Both datasets were spatially aligned with these grid cells using ArcGIS 10.6. Subsequently, species richness was computed within each grid cell. In addition, we also used the R package ‘*sampbias*’ [87] to quantify the specific and combined effects of roads, rivers, airports and cities on recorded occurrence data.

Evolutionary time was represented by the age of the oldest species present within a given grid cell. Diversification rate was represented by the mean diversification rate of all species in the grid cell. Species ages were extracted from the time-calibrated tree, while diversification rate of each species was calculated using the *DR_statistic* function in phytools (Table S10). For the level of deviation from ancestral niche, we first calculate the mean value of MAT, MTWM, and MTDQ in each grid cell. The difference between the present and ancestral states in each grid cell was calculated and expressed by ΔMAT, ΔMTWM, and ΔMTDQ, respectively. Just as the equations below:

ΔMAT = |MAT _present_ – MAT _ancestor_|

ΔMTWM = |MTWM _present_ – MTWM _ancestor_|

ΔMTDQ = |MTDQ _present_ – MTDQ _ancestor_|

Then, the three metrics (ΔMAT, ΔMTWM, and ΔMTDQ) were normalized across a range of 0 to 1, with their summation serving as an indicator to measure the deviation between ancestral and contemporary niche. Grid cells with small value of the metrics and their sum indicate their environmental conditions are similar to the ancestral niche of *Potentilla*. Ultimately, the distributions of species richness, evolutionary time, mean diversification rate, and deviation from ancestral climate were visualized using ArcGIS 10.6. Moreover, their latitudinal trends were modeled via local polynomial regression.

### Electronic supplementary material

Below is the link to the electronic supplementary material.

Supplementary Material 1

## Data Availability

New sequenced and other published plastomes in this study can be found in GenBank (https://www.ncbi.nlm.nih.gov/genbank/), and the accession numbers showed in Table S1.

## Abbreviations

AICc: Corrected Akaike Information Criterion
BAMM: Bayesian analysis of macro-evolutionary mixtures
BAYAREA: BayArea likelihood version of the range evolution model
BS: Bootstrap
ca.: Circa
HiSSE: Hidden state speciation and extinction
HPD: Highest posterior density
LDG: Latitudinal diversity gradient
Ma: Million years ago
MAT: Mean annual temperature
MCMC: Markov chain Monte Carlo
ML: Maximum likelihood
MTDQ: Mean temperature of driest quarter
MTWM: Max temperature of warmest month
MuSSE: Multi-state speciation and extinction
PCA: Principal component analysis
