# Supplementary material for: Radiating diversification and niche conservatism jointly shape the inverse latitudinal diversity gradient of Potentilla L. (Rosaceae)
Source: BMC Plant Biol. 2024 May 23;24:443. doi: 10.1186/s12870-024-05083-8 (PMC11112792; doi:10.1186/s12870-024-05083-8)
Supplement: Supplementary file 1 — Supplementary Material 1 [file 12870_2024_5083_MOESM1_ESM.docx]

**Supporting Information for:**

**Radiating diversification and niche conservatism jointly shape the inverse latitudinal diversity gradient of *Potentilla* L. (Rosaceae)**

Contents:

1. Figures S1 to S14
2. Tables S1 to S11
3. References


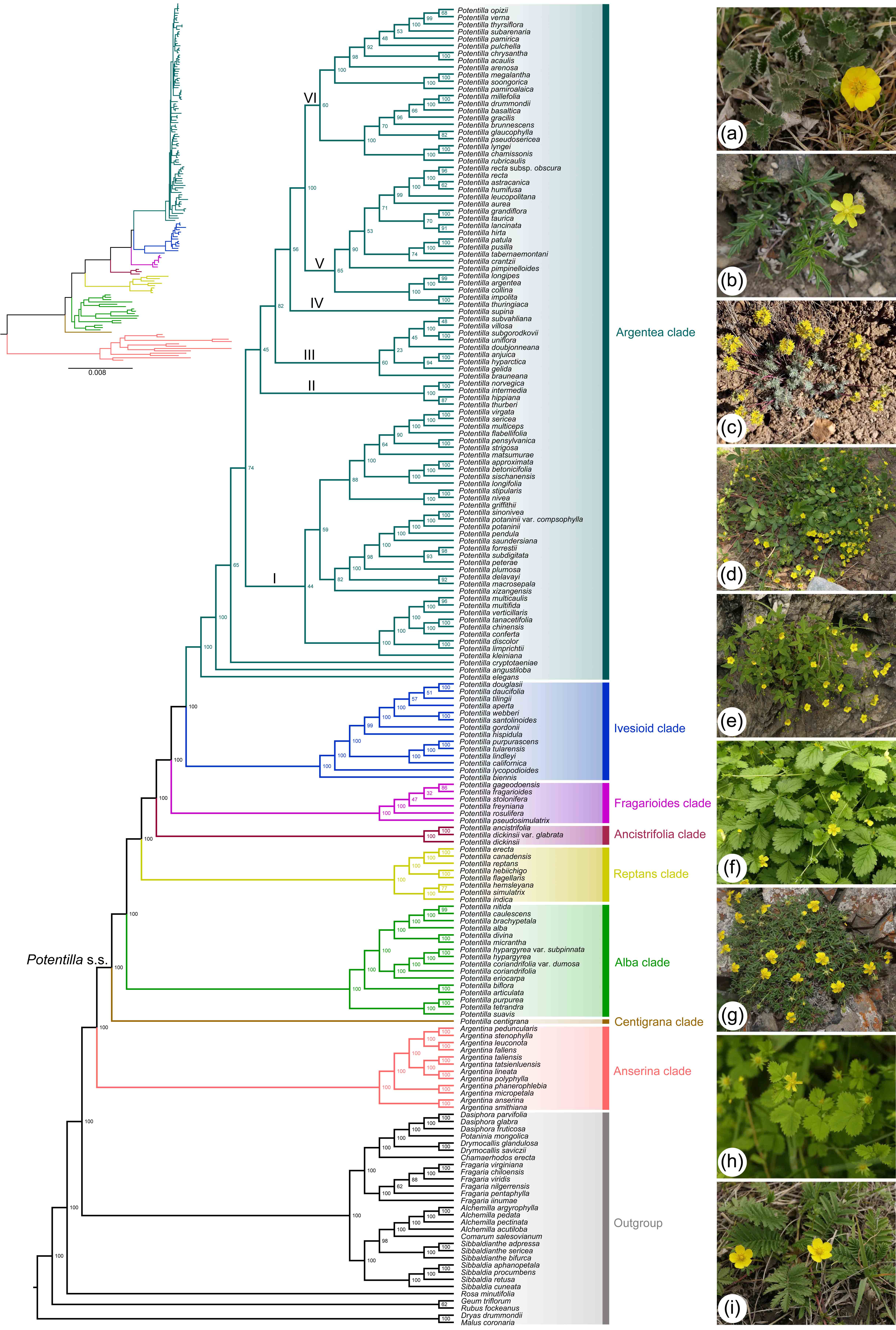


**Figure S1** ML tree of *Potentilla* inferred from whole plastomes. Numbers above branches are bootstrap values. Photographs at right: (a) *P*. *nivea*, (b) *P*. *sischanensis*, (c) *P. webberi*, (d) *P*. *fragarioides*, (e) *P*. *ancistrifolia*, (f) *P*. *simulatrix*, (g) *P*. *biflora*, and (h) *Argentina anserina*. Except for *P. webberi* (*Ivesia webberi*) (https://www.inaturalist.org/photos/131054658), photographs are by the authors of this article.

**
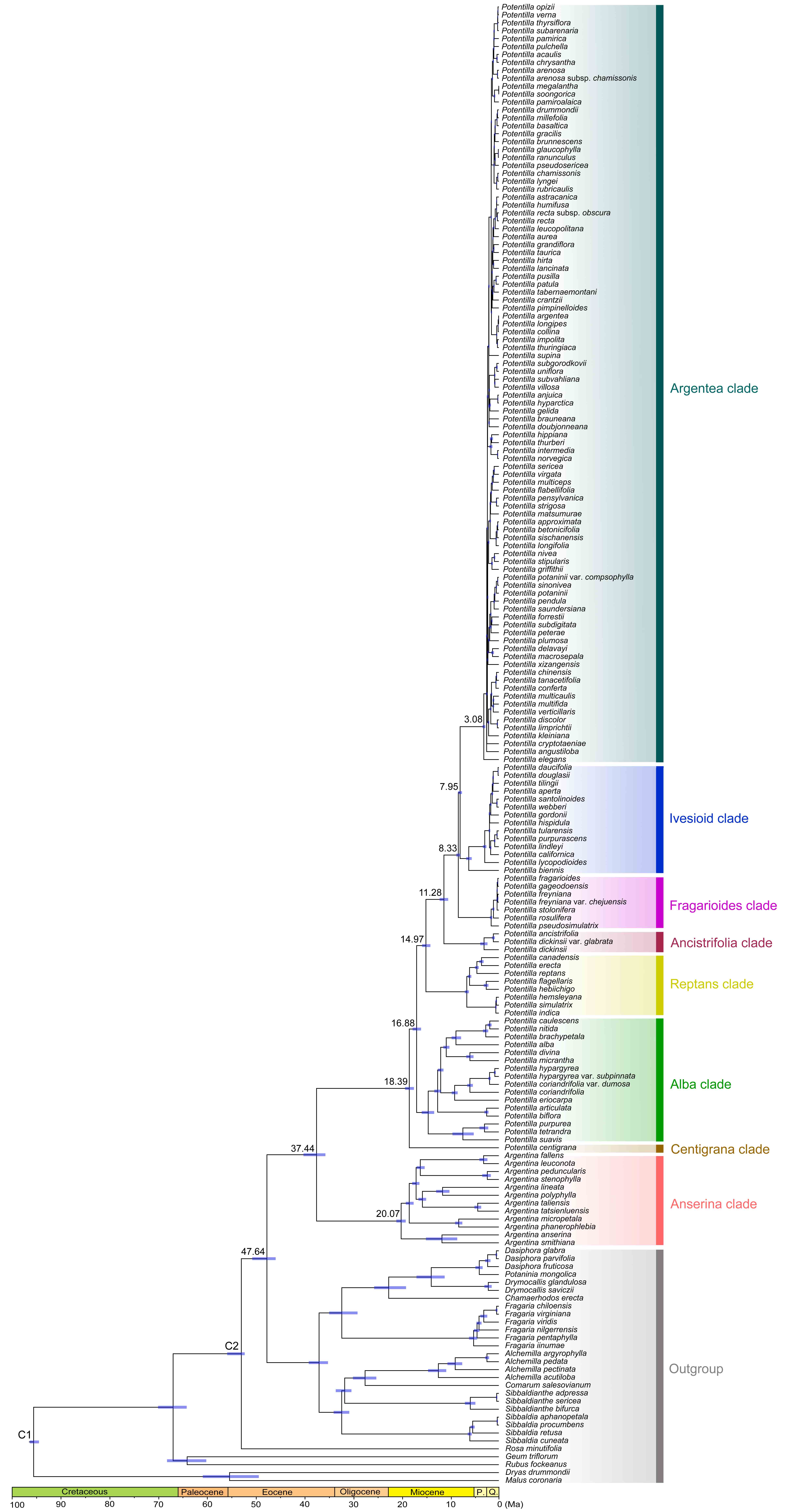
**

**Figure S2** Chronogram of *Potentilla* estimated in treePL. Blue bars represent 95% highest posterior density (HPD) intervals for mean node ages. The C1 and C2 indicate the placements of calibration points. P. = Pliocene; Q. = Quaternary.

**

**

**Figure S3** The simulated time-calibrated tree of *Potentilla*. The species labeled by red are added randomly.


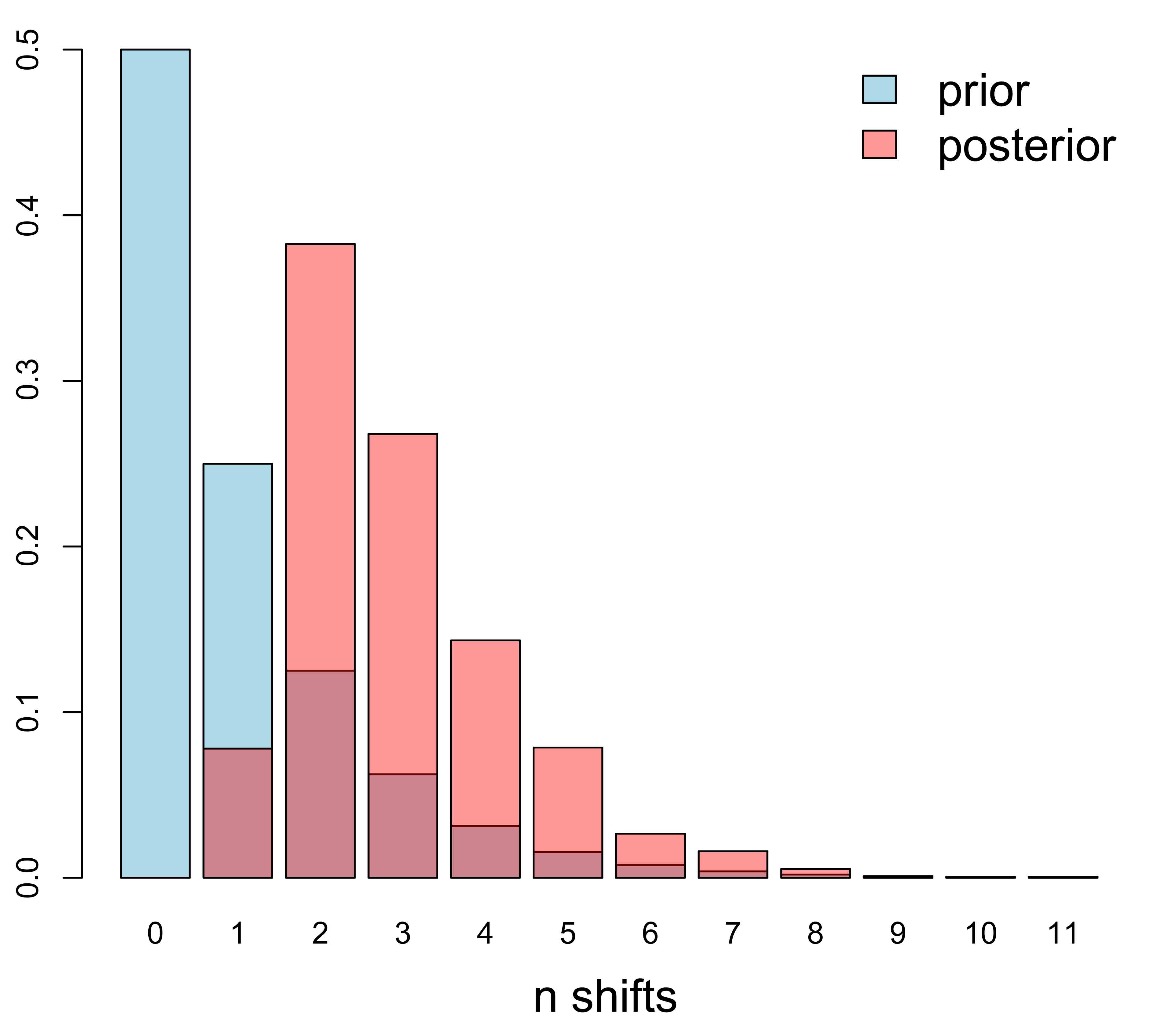


**Figure S4** Posterior probability of each shift rate in the BAMM analysis.


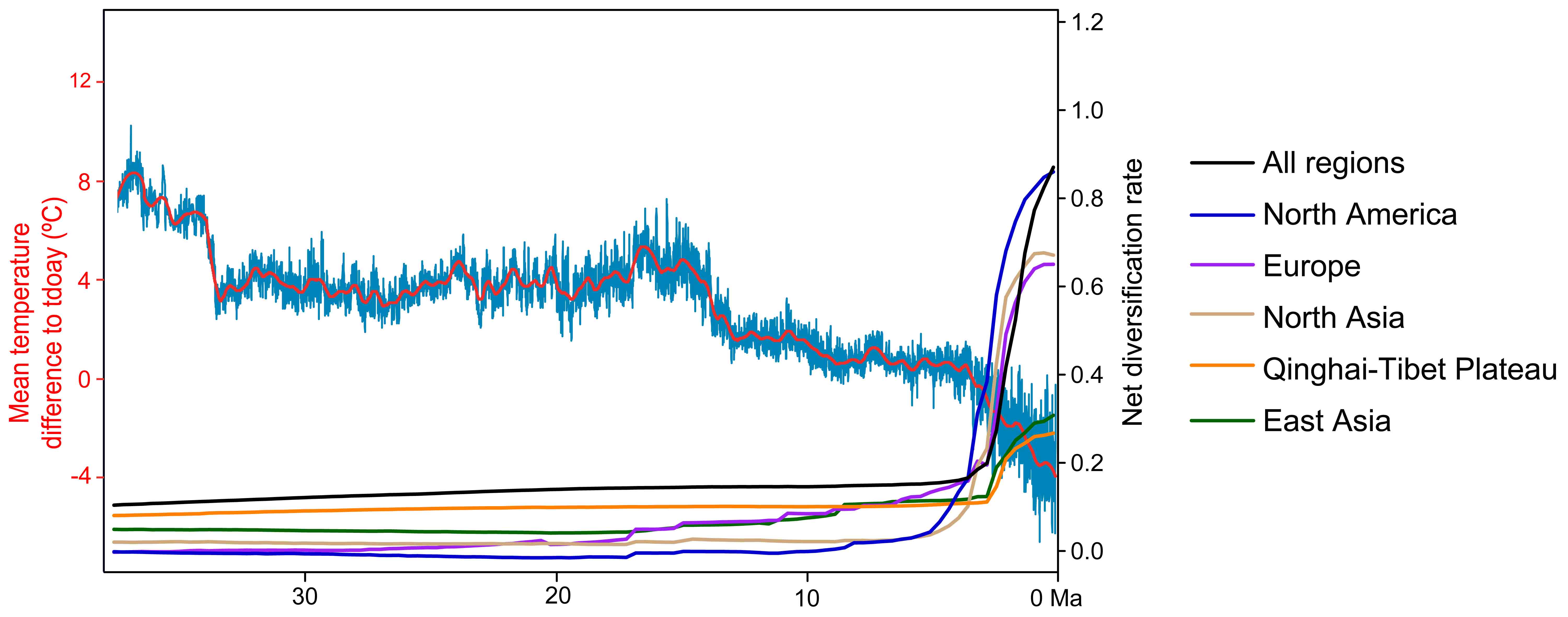


**Figure S5** The temporal patterns in the net diversification rate of *Potentilla* across the globe and five biogeographic regions estimated by the simulated time-calibrated tree. Red curve shows global temperature differences over the last 37.44 Ma as

compared to current temperature and is modified from Westerhold et al. [1].


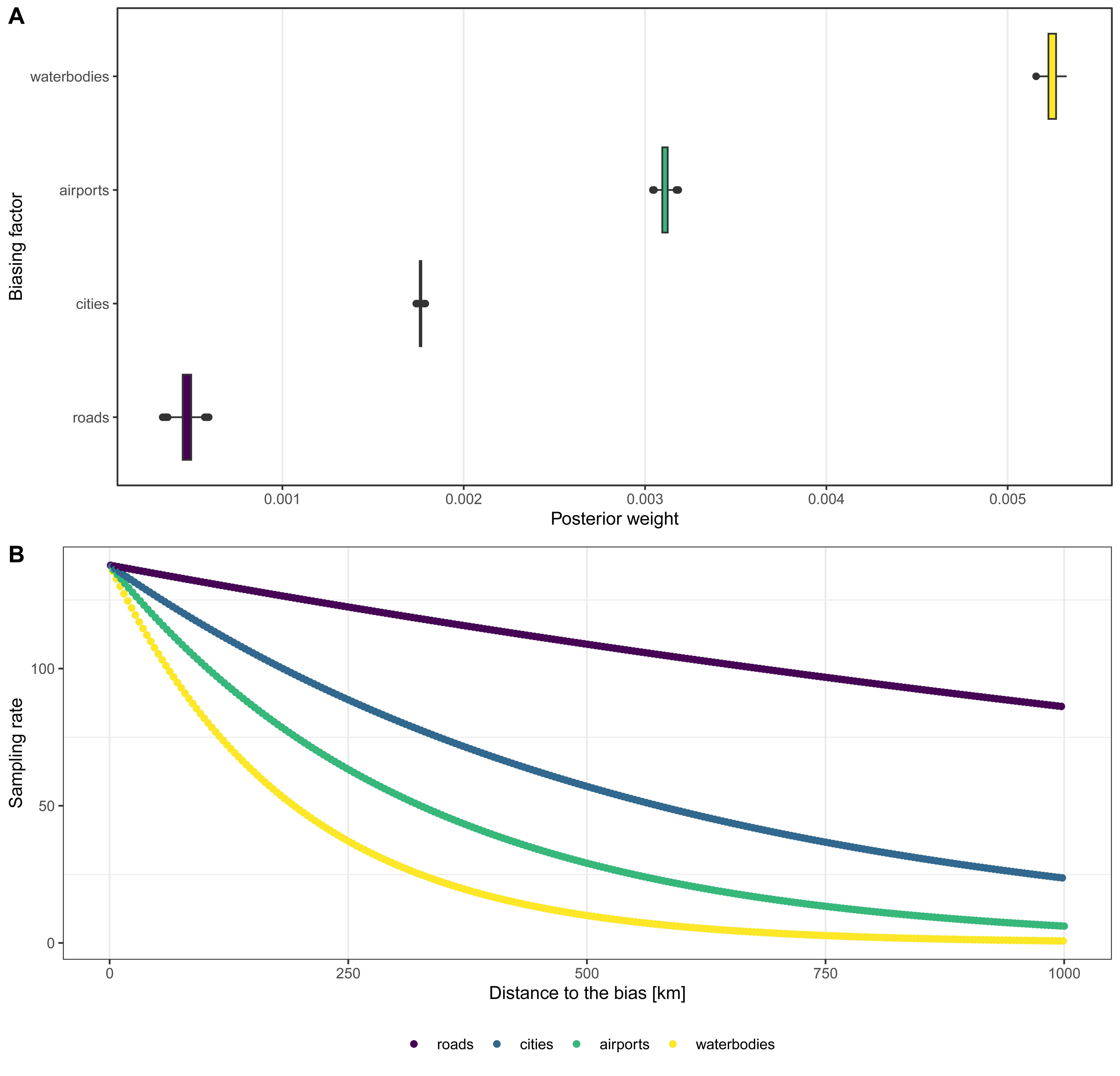


**Figure S6** Results of the empirical validation analysis, estimating the accessibility bias in *Potentilla* occurrences. (a) bias weights defining the effects of each bias factor, (b) sampling rate as function of distance to the closest instance of each bias factor (the expected number of occurrences) given the inferred sampbias model. At the study scale of 0.05 degrees (ca 5 × 5 km) sampbias finds the strongest biasing effect for the proximity of waterbodies and airports.


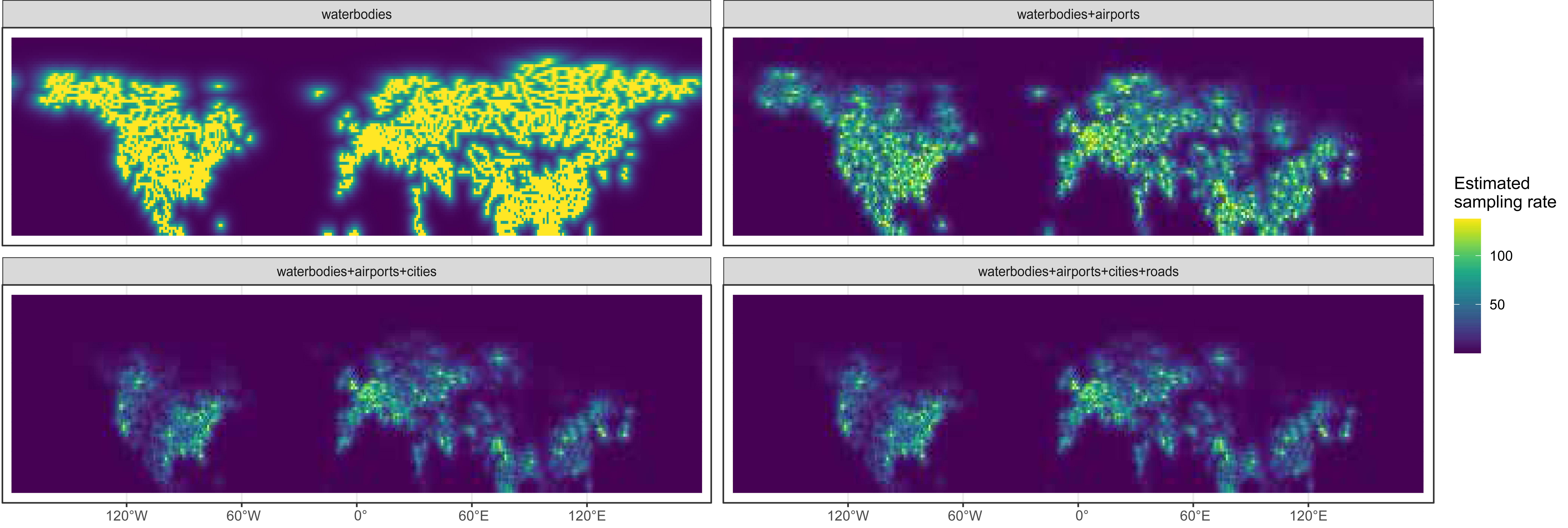


**Figure S7** Spatial projection of the sampling bias in an empirical example data set of *Potentilla* occurrences on the Northern Hemisphere. The colors show the projection of sampling rates given the inferred sampbias model. The highest undersampling is in the Northeast and Central Asia.





**Figure S8** Ancestral thermal niches reconstruction based on the empirical time-calibrated tree. (A) mean annual temperature; (B) max temperature of warmest month; (C) Mean temperature of driest quarter.

**

Figure S9** Ancestral thermal niches reconstruction based on the simulated time-calibrated tree. (A) mean annual temperature; (B) max temperature of warmest month; (C) Mean temperature of driest quarter.





**Figure S10** Distribution pattern of ΔMAT, ΔMTWM, and ΔMTDQ.





**Figure S11** Distribution pattern of the eight clades of *Potentilla* calculated based on the 451-dataset. (A) Argentea clade; (B) Ivesioid clade; (C) Fragarioides clade; (D) Ancistrifolia clade; (E) Reptans clade; (F) Alba clade; (G) Centigrana clade; (H) Anserina clade.


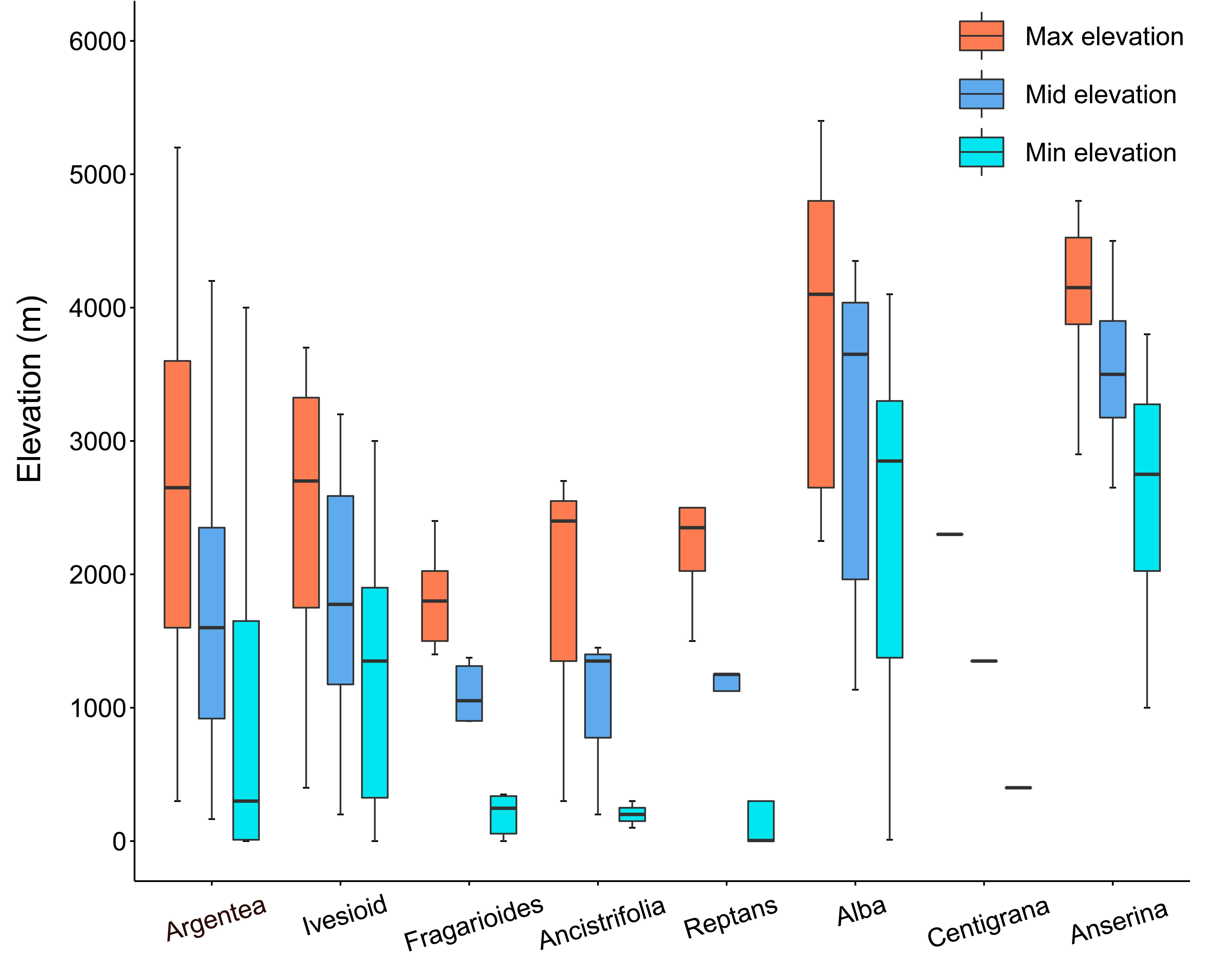


**Figure S12** Elevation of the eight clades of *Potentilla.* The species elevation ranges were obtained from *Flora of China* [2], *Flora of North American* [3, 4], *Flora Europaea* [5], *Flora URSS* [6], and the herbarium specimens in JSTOR (https://plants.jstor.org/) and Chinese Virtual Herbarium (http://www.cvh.ac.cn/).


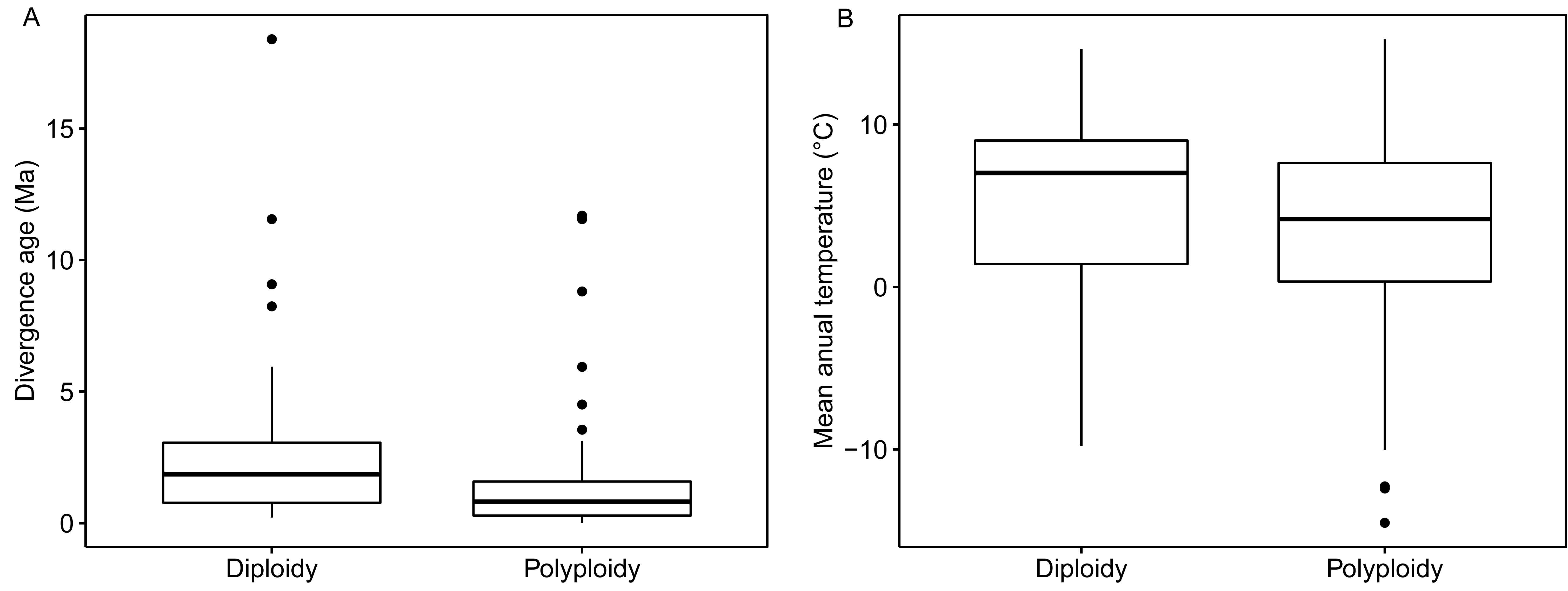


**Figure S13** The divergence ages and mean annual temperatures of diploidy and polyploidy of *Potentilla*.


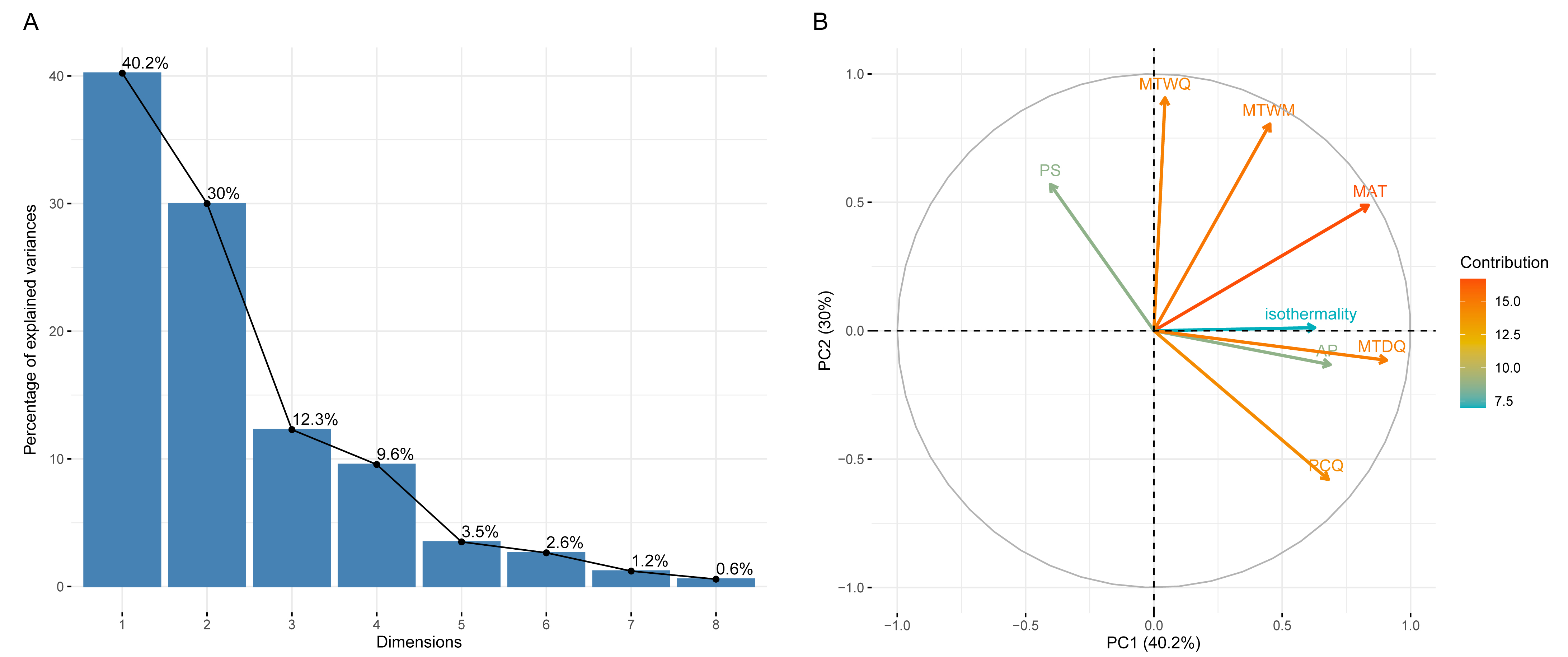


**Figure S14** PCA results of eight bioclimatic variables representing climate lability of *Potentilla* species. (a) The percentage of explained variance is provided as a scree plot. (b) PCA biplot shows the contribution of eight bioclimatic variables to first two PCs.

**Table S1** Taxon names, voucher information and GenBank accession numbers. (* indicate that the raw sequence data was downloaded from SRA database of NCBI).

| **Species** | **Clade** | **Voucher** | **Locality** | **GenBank accession** |
| --- | --- | --- | --- | --- |
| *Potentilla alba* L. | Alba | D.Y.Hong s.n. (PE) | Austria | OQ819109 |
| *Potentilla articulata* Franch. | Alba | PE-Xizang Expedition 12521 (PE) | Xizang, China | OQ819108 |
| *Potentilla biflora* D.F.K.Schltdl. | Alba | W.B.Xu & T.T.Xue Y15498 (PE) | Xinjiang, China | OQ835057 |
| *Potentilla brachypetala* Fisch. & C.A.Mey. ex Lehm. | Alba | N.Desoulavy 714 (PE) | Caucasus, Russia | OQ835058 |
| *Potentilla caulescens* L. | Alba | s.n. 1252 (PE) | Hungary | OQ835062 |
| *Potentilla coriandrifolia* G.Don | Alba | Xizang Expedition 2366 (PE) | Xizang, China | **OQ835069** |
| *Potentilla divina* Albov | Alba | A.Kolakovsky et V.Jabrova. 3123 (PE) | Abkhazia, Georgia | OQ835076 |
| *Potentilla coriandrifolia* var. *dumosa* Franch. | Alba | T.T.Xue Y12627 (PE) | Yunnan, China | OQ835070 |
| *Potentilla eriocarpa* Wall. ex Lehm. | Alba | T.T.Xue Y12629 (PE) | Yunnan, China | OQ835081 |
| *Potentilla hypargyrea* Hand.-Mazz. | Alba | T.T.Yu 22204 (PE) | Yunnan, China | **OQ835094** |
| *Potentilla hypargyrea* var. *subpinnata* T.T.Yu & C.L.Li | Alba | W.B.Xu et al. QTP289 (PE) | Xizang, China | OQ835095 |
| *Potentilla micrantha* Ramond ex DC. | Alba | — | — | HG931056 |
| *Potentilla nitida* L. | Alba | Schönach 820 (PE) | Hungary | OQ835112 |
| *Potentilla purpurea* (Royle) Hook.f. | Alba | — | — | KY419953 |
| *Potentilla suavis* Soják | Alba | — | — | MT114190 |
| *Potentilla tetrandra* (Bunge) Hook.f. | Alba | W.B.Xu & T.T.Xue Y15537 (PE) | Xinjiang, China | OQ835153 |
| *Potentilla ancistrifolia* Bunge | Ancistrifolia | T.T.Xue & X.D.Yang Y12632 (PE) | Henan, China | OQ835046 |
| *Potentilla dickinsii* Franch. & Sav. | Ancistrifolia | S.X.Yu et al. Y12028 (PE) | Hebei, China | OQ835074 |
| *Potentilla dickinsii* var. *glabrata* Nakai | Ancistrifolia | — | — | MT412406 |
| *Argentina anserina* (L.) Rydb. | Anserina | W.B.Xu & T.T.Xue Y12447 (PE) | Qinhai, China | OQ835025 |
| *Argentina fallens* (Cardot) Soják | Anserina | W.B.Xu et al. Y12293 (PE) | Sichuan, China | **OQ835026** |
| *Argentina leuconota* (D.Don) Soják | Anserina | T.T.Xue Y12646 (PE) | Yunnan, China | OQ835027 |
| *Argentina lineata* (Trevir.) Soják | Anserina | — | — | MT677853 |
| *Argentina micropetala* (D.Don) Soják | Anserina | — | — | KY420021 |
| *Argentina peduncularis* (D.Don) Soják | Anserina | W.B.Xu et al. QTP708P (PE) | Yunnan, China | **OQ835028** |
| *Argentina phanerophlebia* (T.T.Yu & C.L.Li) T.Feng & Heng C.Wang | Anserina | — | — | MT114192 |
| *Argentina polyphylla* (Wall. ex Lehm.) Soják | Anserina | W.B.Xu et al. QTP167 (PE) | Yunnan, China | OQ835029 |
| *Argentina smithiana* (Hand.-Mazz.) Soják | Anserina | T.T.Xue Y12561 (PE) | Sichuan, China | **OQ835030** |
| *Argentina stenophylla* (Franch.) Soják | Anserina | W.B.Xu et al. QTP288 (PE) | Xizang, China | OQ835031 |
| *Argentina taliensis* (W.W.Sm.) Soják | Anserina | T.T.Xue Y12645 (PE) | Yunnan, China | **OQ835032** |
| *Argentina tatsienluensis* (Th.Wolf) Soják | Anserina | W.B.Xu et al. QTP670 (PE) | Xizang, China | **OQ835033** |
| *Potentilla acaulis* L. | Argentea | T.T.Xue Y12507 (PE) | Gansu, China | OQ835045 |
| *Potentilla angustiloba* T.T.Yu & C.L.Li | Argentea | W.B.Xu & T.T.Xue Y15454 (PE) | Xinjiang, China | OQ835047 |
| *Potentilla anjuica* V.V.Petrovsky | Argentea | V.Petrovsky 6177 (PE) | Magadan, Russia | OQ835048 |
| *Potentilla approximata* Bunge | Argentea | H.Krascheninnikov 7473 (PE) | Chelyabinsk,Russia | OQ835049 |
| *Potentilla arenosa* (Turcz.) Juz. | Argentea | T.Koroleva 6036 (PE) | Nizhnekolymsk, Russia | OQ835050 |
| *Potentilla argentea* L. | Argentea | T.T.Xue Y12448 (PE) | Beijing, China | OQ835051 |
| *Potentilla astracanica* Jacq. | Argentea | О. Л. Ловелиус et al. 1432149 (PE) | Volgograd, Russia | OQ835052 |
| *Potentilla aurea* L. | Argentea | J.D.Chen 94273 (PE) | Switzerland | OQ835053 |
| *Potentilla basaltica* Tiehm & Ertter | Argentea | Arnold Tiehm 7770 (PE) | Nevada, USA | OQ835054 |
| *Potentilla betonicifolia* Poir. | Argentea | T.T.Xue & X.D.Yang Y12457 (PE) | Hebei, China | OQ835055 |
| *Potentilla brachyloba* (Borbás) Zimmeter | Argentea | Borbás 2834 (PE) | Hungary | OQ835156 |
| *Potentilla brauneana* Hoppe ex Nestl. | Argentea | Huter 844 (PE) | Hungary | OQ835059 |
| *Potentilla brunnescens* Rydb. | Argentea | Richard R. Halse 9749 (PE) | Idaho, USA | OQ835060 |
| *Potentilla chamissonis* Hultén | Argentea | ERR5554618* | — | **OQ835064** |
| *Potentilla chinensis* Ser. | Argentea | S.X.Yu et al. Y12126 (PE) | Beijing, China | OQ835065 |
| *Potentilla chrysantha* Trevir. | Argentea | W.B.Xu & T.T.Xue Y15578 (PE) | Xinjiang, China | OQ835066 |
| *Potentilla collina* Wibel | Argentea | Erik Asplund s.n. (PE) | Sweden | OQ835067 |
| *Potentilla conferta* Bunge | Argentea | T.T.Xue & X.D.Yang Y12453 (PE) | Hebei, China | OQ835068 |
| *Potentilla crantzii* (Crantz) Beck ex Fritsch | Argentea | B.S.Li s.n. (PE) | Tromso, Norway | OQ835071 |
| *Potentilla cryptotaeniae* Maxim. | Argentea | The fourth team of Forest Plant Survey of Northeast China 9542004 (PE) | Jilin, China | OQ835072 |
| *Potentilla delavayi* Franch. | Argentea | T.T.Yu 16936 (PE) | Yunnan, China | OQ835073 |
| *Potentilla discolor* Bunge | Argentea | T.T.Xue Y12646C (PE) | Beijing, China | OQ835075 |
| *Potentilla doubjonneana* Cambess. | Argentea | J.Soják 2698 (PE) | Kirgisia | OQ835077 |
| *Potentilla drummondii* Lehm. | Argentea | Richard R. Halse 9194 (PE) | Oregon, USA | OQ835078 |
| *Potentilla elegans* Cham. & Schltdl. | Argentea | Berkutenko A.N.& Polezhaev A.N. 71 (PE) | Chukotskiy, Russia | OQ835079 |
| *Potentilla flabellifolia* Hook. ex Torr. & A.Gray | Argentea | Richard R. Halse 8719 (PE) | Oregon, USA | OQ835082 |
| *Potentilla forrestii* W.W.Sm. | Argentea | T.T.Xue Y12626 (PE) | Yunnan, China | OQ835138 |
| *Potentilla gelida* C. A. Mey. | Argentea | W.B.Xu & T.T.Xue Y15517 (PE) | Xinjiang, China | OQ835085 |
| *Potentilla glaucophylla* Lehm. | Argentea | A.Tiehm & J.Nachlinger 17972 (PE) | Nevada, USA | OQ835086 |
| *Potentilla gracilis* Douglas ex Hook. | Argentea | D.E.Breedlove et al. 62659 (PE) | Nevada, USA | OQ835087 |
| *Potentilla grandiflora* L. | Argentea | s.n. 81582 (PE) | France | OQ835088 |
| *Potentilla griffithii* Hook.f. | Argentea | W.B.Xu et al. QTP473 (PE) | Xizang, China | OQ835089 |
| *Potentilla hippiana* Lehm. | Argentea | W.Hess 2184 (PE) | New Mexico, USA | OQ835090 |
| *Potentilla hirta* L. | Argentea | s.n. s.n. (PE) | France | OQ835091 |
| *Potentilla humifusa* Willd. ex Schltdl. | Argentea | О. Каеръ 2162 (PE) | Yekaterinburg, Russia | OQ835092 |
| *Potentilla hyparctica* Malte | Argentea | O.Rebristaja. 6573 (PE) | Tyumen, Russia | OQ835093 |
| *Potentilla impolita* Wahlenb. | Argentea | Gy.Szollat s.n. (PE) | Hungary | OQ835096 |
| *Potentilla intermedia* L. | Argentea | H.E.Ahles 77732 (PE) | Massachusetts, USA | OQ835097 |
| *Potentilla kleiniana* Wight & Arn. | Argentea | W.B.Xu et al. QTP068 (PE) | Yunnan, China | OQ835098 |
| *Potentilla lancinata* Cardot | Argentea | T.T.Xue Y12597 (PE) | Yunnan, China | OQ835099 |
| *Potentilla leucopolitana* P.J.Müll. | Argentea | Zimmeter & Blocki 447 (PE) | Hungary | OQ835100 |
| *Potentilla limprichtii* J.Krause | Argentea | C.M.Tan 7066 (PE) | Jiangxi, China | OQ835101 |
| *Potentilla longifolia* Willd. ex D.F.K.Schltdl. | Argentea | S.X.Yu et al. Y12032 (PE) | Hebei, China | OQ835102 |
| *Potentilla longipes* Ledeb. | Argentea | s.n. 125 (PE) | Kazakhstan | OQ835103 |
| *Potentilla lyngei* Jurtzev & Soják | Argentea | ERR5529626* | — | **OQ835104** |
| *Potentilla macrosepala* Cardot | Argentea | H.T.Tsai 58012 (PE) | Yunnan, China | OQ835105 |
| *Potentilla matsumurae* Th.Wolf | Argentea | M.Furuse 13766 (PE) | Yamanashi-ken, Japan | OQ835106 |
| *Potentilla megalantha* Takeda | Argentea | K.Kondo 2072 (PE) | Hokkaido, Japan | OQ835107 |
| *Potentilla millefolia* Rydb. | Argentea | B.Bartholomew et al. 2357 (PE) | California, USA | OQ835108 |
| *Potentilla multicaulis* Bunge | Argentea | W.B.Xu & T.T.Xue Y12488 (PE) | Gansu, China | OQ835109 |
| *Potentilla multiceps* T.T.Yu & C.L.Li | Argentea | Qinghai-Xizang Expedition-Vegetation Group 13165 (PE) | Xizang, China | OQ835110 |
| *Potentilla multifida* L. | Argentea | W.B.Xu & T.T.Xue Y12461 (PE) | Qinhai, China | OQ835111 |
| *Potentilla nivea* L. | Argentea | S.X.Yu et al. Y12076 (PE) | Hebei, China | OQ835113 |
| *Potentilla norvegica* L. | Argentea | M.A.Vincent & M.W.Vincent 15822 (PE) | Michigan, USA | OQ835114 |
| *Potentilla opizii* Domin | Argentea | Jos. Rohlena. 70 (PE) | Bohemia, Czechia | **OQ835115** |
| *Potentilla pamirica* Th.Wolf | Argentea | s.n. 19 (PE) | Russia | OQ835116 |
| *Potentilla pamiroalaica* Juz. | Argentea | W.B.Xu & T.T.Xue Y15539 (PE) | Xinjiang, China | OQ835117 |
| *Potentilla patula* Waldst. & Kit. | Argentea | Z.Saryczeva s.n. (PE) | Hungary | **OQ835118** |
| *Potentilla pendula* T.T.Yu & C.L.Li | Argentea | G.H.Yang 58788 (PE) | Chongqing, China | OQ835119 |
| *Potentilla pensylvanica* L | Argentea | Richard R.Halse 8676 (PE) | Utah, USA | OQ835120 |
| *Potentilla peterae* Hand.-Mazz. | Argentea | W.B.Xu & T.T.Xue Y12458 (PE) | Qinhai, China | OQ835121 |
| *Potentilla pimpinelloides* L. | Argentea | В. Хитрово. 2170 (PE) | Orel, Russia | OQ835122 |
| *Potentilla plumosa* T.T.Yu & C.L.Li | Argentea | W.B.Xu & T.T.Xue Y12408 (PE) | Sichuan, China | OQ835123 |
| *Potentilla potaninii* Th.Wolf | Argentea | W.B.Xu & T.T.Xue Y12438 (PE) | Qinhai, China | OQ835124 |
| *Potentilla potaninii* var. *compsophylla* (Hand.-Mazz.) T.T.Yu & C.L.Li | Argentea | T.T.Xue Y12536 (PE) | Sichuan, China | OQ835125 |
| *Potentilla pseudosericea* Rydb. | Argentea | J.D.Morefield et al. 4619 (PE) | California, USA | OQ835126 |
| *Potentilla pulchella* R.Br. | Argentea | ERR5554972* | — | **OQ835128** |
| *Potentilla pusilla* Host | Argentea | Sarnthein 829 (PE) | Hungary | OQ835129 |
| *Potentilla recta* L. | Argentea | T.T.Xue Y16581 (PE) | Beijing, China | OQ835130 |
| *Potentilla recta* subsp. *obscura* (Willd.) Arcang. | Argentea | Steinitz 1242 (PE) | Hungary | **OQ835131** |
| *Potentilla rubricaulis* Lehm. | Argentea | ERR5529647* | — | **OQ835135** |
| *Potentilla saundersiana* Royle | Argentea | W.B.Xu & T.T.Xue Y12406 (PE) | Sichuan, China | OQ835136 |
| *Potentilla sericea* L. | Argentea | W.B.Xu & T.T.Xue Y15551 (PE) | Xinjiang, China | OQ835139 |
| *Potentilla sinonivea* Hultén | Argentea | W.B.Xu et al. QTP652P (PE) | Xizang, China | OQ835137 |
| *Potentilla sischanensis* Bunge ex Lehm. | Argentea | W.B.Xu & T.T.Xue Y12462 (PE) | Qinhai, China | OQ835141 |
| *Potentilla soongorica* Bunge | Argentea | H.Sun et al. 17250 (PE) | Uzbekistan | OQ835142 |
| *Potentilla stipularis* L. | Argentea | N.Antonova & T.Zaslavskaya 1831 (PE) | Yakutsk, Russia | OQ835143 |
| *Potentilla strigosa* Pall. ex Pursh | Argentea | W.B.Xu & T.T.Xue Y15591 (PE) | Xinjiang, China | OQ835144 |
| *Potentilla subarenaria* Borbás ex Zimmeter | Argentea | K.R.Kupffer 1568 (PE) | Courland, Latvia | OQ835145 |
| *Potentilla subdigitata* Yü et Li | Argentea | B.S.Li et al. 10574 (PE) | Xinjiang, China | OQ835146 |
| *Potentilla subgorodkovii* Jurtzev | Argentea | ERR5529426* | — | **OQ835147** |
| *Potentilla subvahliana* Jurtzev | Argentea | ERR5554849* | — | **OQ835148** |
| *Potentilla supina* L. | Argentea | T.T.Xue Y12646A (PE) | Beijing, China | OQ835149 |
| *Potentilla tabernaemontani* Asch. | Argentea | Danert 119 (PE) | Harzvorland, Germany | **OQ835150** |
| *Potentilla tanacetifolia* Willd. ex D.F.K.Schltdl. | Argentea | S.X.Yu et al. Y12033 (PE) | Hebei, China | OQ835151 |
| *Potentilla taurica* Willd. ex Schltdl. | Argentea | Zimmeter 1241 (PE) | Hungary | OQ835152 |
| *Potentilla thurberi* A.Gray | Argentea | W.Hess 2163 (PE) | New Mexico, USA | OQ835154 |
| *Potentilla thuringiaca* Bernh. ex Link | Argentea | Erik Evers s.n. (PE) | Sweden | OQ835155 |
| *Potentilla uniflora* Ledeb. | Argentea | ERR5529438* | — | **OQ835157** |
| *Potentilla verna* L. | Argentea | s.n. s.n. (PE) | France | OQ835158 |
| *Potentilla verticillaris* Stephan ex Willd. | Argentea | T.T.Xue & X.D.Yang Y12463 (PE) | Hebei, China | OQ835159 |
| *Potentilla villosa* Pall. ex Pursh | Argentea | ERR5529405* | — | **OQ835160** |
| *Potentilla virgata* Lehm. | Argentea | W.B.Xu & T.T.Xue Y15611 (PE) | Xinjiang, China | OQ835161 |
| *Potentilla xizangensis* T.T.Yu & C.L.Li | Argentea | Xizang Expedition 5741 (PE) | Xizang, China | OQ835162 |
| *Potentilla centigrana* Maxim. | Centigrana | T.T.Xue & X.D.Yang Y12576 (PE) | Shaanxi, China | OQ835063 |
| *Potentilla fragarioides* L. | Fragarioides | T.T.Xue Y12646B (PE) | Beijing, China | OQ835084 |
| *Potentilla freyniana* Bornm. | Fragarioides | — | — | MK209638 |
| *Potentilla gageodoensis* M.Kim | Fragarioides | — | — | MT259043 |
| *Potentilla pseudosimulatrix* W.B.Liao, Si Feng Li & Z.Y.Yu | Fragarioides | T.T.Xue & X.D.Yang Y12631 (PE) | Shaanxi, China | OQ835127 |
| *Potentilla rosulifera* H.Lév. | Fragarioides | M.Furuse 53273 (PE) | Kumamoto, Japan | OQ835134 |
| *Potentilla stolonifera* Lehm. ex Ledeb. | Fragarioides | — | — | MK227179 |
| *Potentilla aperta* J.T.Howell | Ivesioid | A.Tiehm 16764 (PE) | Nevada, USA | OQ835040 |
| *Potentilla biennis* Greene | Ivesioid | Richard R. Halse 4670 (PE) | Oregon, USA | OQ835056 |
| *Potentilla californica* (Cham. & Schltdl.) Greene | Ivesioid | J.T.Howell 31565 (PE) | California, USA | OQ835034 |
| *Potentilla daucifolia* Greene | Ivesioid | J.T.Howell et al. 53448 (PE) | California, USA | OQ835036 |
| *Potentilla douglasii* Greene | Ivesioid | Richard R. Halse 9812 (PE) | Oregon, USA | OQ835037 |
| *Potentilla gordonii* (Hook.) Greene | Ivesioid | P.B.Marcum et al. 4039 (PE) | Wyoming, USA | OQ835041 |
| *Potentilla hispidula* (Rydb.) Jeps. | Ivesioid | J.D.Morefield & D.H.McCarty 4165 (PE) | California, USA | OQ835038 |
| *Potentilla* *lindleyi* Greene | Ivesioid | J.Stone & S.Bodine 2978 (PE) | California, USA | OQ835035 |
| *Potentilla* *lycopodioides* (A.Gray) Baill. ex J.T.Howell | Ivesioid | E.C.Twisselmann 16895 (PE) | Nevada, USA | OQ835042 |
| *Potentilla purpurascens* (S.Watson) Greene | Ivesioid | — | — | KY419979 |
| *Potentilla santolinoides* (A.Gray) Greene | Ivesioid | J.T.Howell 34436 (PE) | California, USA | OQ835043 |
| *Potentilla tilingii* (Regel) Greene | Ivesioid | — | — | KY420028 |
| *Potentilla tularensis* J.T.Howell | Ivesioid | J.T.Howell et al. 53871 (PE) | Nevada, USA | OQ835039 |
| *Potentilla webberi* (A.Gray) Greene | Ivesioid | A.Tiehm 16033 (PE) | Nevada, USA | OQ835044 |
| *Potentilla erecta* (L.) Raeusch. | Reptans | A. Quintanar et al. AQ3705 (PE) | Portugal | OQ835080 |
| *Potentilla flagellaris* D.F.K.Schltdl. | Reptans | T.T.Xue Y12449 (PE) | Beijing, China | OQ835083 |
| *Potentilla hebiichigo* Yonek. & H.Ohashi | Reptans | — | — | MK301251 |
| *Potentilla hemsleyana* Th.Wolf | Reptans | T.T.Xue & X.D.Yang Y12639 (PE) | Shanxi, China | OQ835133 |
| *Potentilla indica* (Andrews) Th.Wolf | Reptans | — | — | MK134678 |
| *Potentilla reptans* L. | Reptans | M. Wayda 7676 (PE) | Poland | OQ835132 |
| *Potentilla simulatrix* Th.Wolf | Reptans | T.T.Xue Y12505 (PE) | Gansu, China | OQ835140 |
| *Potentilla canadensis* L. | Rtptans | P.B.Marcum et al. 3036 (PE) | North Carolina, USA | OQ835061 |
| *Alchemilla acutiloba* Opiz | outgroup | — | — | KY420009 |
| *Alchemilla argyrophylla* Oliv. | outgroup | — | — | MT382661 |
| *Alchemilla pectinata* Kunth | outgroup | — | — | KY419937 |
| *Alchemilla pedata* Hochst. ex A.Rich. | outgroup | — | — | MT382662 |
| *Chamaerhodos erecta* (L.) Bunge | outgroup | — | — | KY420001 |
| *Comarum salesovianum* (Stephan) Ledeb. | outgroup | — | — | MT017928 |
| *Dasiphora fruticosa* (L.) Rydb. | outgroup | — | — | KY420016 |
| *Dasiphora glabra* (G.Lodd.) Soják | outgroup | — | — | MW092109 |
| *Dasiphora parvifolia* (Fisch. ex Lehm.) Juz. | outgroup | — | — | NC053696 |
| *Dryas drummondii* Richardson ex Hook. | outgroup | — | — | KY419952 |
| *Drymocallis glandulosa* (Lindl.) Rydb. | outgroup | — | — | KY420015 |
| *Drymocallis saviczii* (I.Schischk. & Kom.) Soják | outgroup | — | — | MT178809 |
| *Fragaria chiloensis* (L.) Mill. | outgroup | — | — | JN884816 |
| *Fragaria iinumae* Makino | outgroup | — | — | KC507759 |
| *Fragaria nilgerrensis* Schltdl. ex J.Gay | outgroup | — | — | MK560340 |
| *Fragaria pentaphylla* Losinsk. | outgroup | — | — | KY434061 |
| *Fragaria virginiana* Mill. | outgroup | — | — | JN884817 |
| *Fragaria viridis* Weston | outgroup | — | — | MH938454 |
| *Geum triflorum* Pursh | outgroup | — | — | KY419977 |
| *Malus coronaria* (L.) Mill. | outgroup | — | — | MN068247 |
| *Potaninia mongolica* Maxim. | outgroup | — | — | MN691039 |
| *Rosa minutifolia* Engelm. | outgroup | — | — | MT755634 |
| *Rubus fockeanus* Kurz | outgroup | C.S.Chang et al. NE020481 (PE) | Rolwaling, Nepal | OQ835163 |
| *Sibbaldia aphanopetala* Hand.-Mazz. | outgroup | — | — | MT178810 |
| *Sibbaldia cuneata* Edgew. | outgroup | W.B.Xu et al. QTP758P (PE) | Yunnan, China | OQ835164 |
| *Sibbaldia procumbens* L. | outgroup | — | — | KY419935 |
| *Sibbaldia retusa* (O.F.Müll.) T.Erikss. | outgroup | — | — | KY420036 |
| *Sibbaldianthe adpressa* (Bunge) Juz. | outgroup | — | — | MT114191 |
| *Sibbaldianthe bifurca* (L.) Kurtto & T.Erikss. | outgroup | — | — | NC052882 |
| *Sibbaldianthe sericea* Grubov | outgroup | — | — | KY419993 |

**Table S2** The 302 *Potentilla* species without plastomes that used to add on time-calibrated tree. The locality of each species was obtained from Plant of the World Online (POWO, https://powo.science.kew.org/).

| **Species** | **Locality** | **Clade** | **Evidence(s) for determining phylogenetic placement** | **Reference(s)** |
| --- | --- | --- | --- | --- |
| *Potentilla alchimilloides* | SC. France to N. & C. Spain | Alba | morphology | Wolf [7] |
| *Potentilla apennina* | SE. Europe | Alba | molecular analysis | Dobeš and Paule [8] |
| *Potentilla arcadiensis* | Greece | Alba | morphology | Iatroú [9] |
| *Potentilla carniolica* | Slovenia to Croatia | Alba | morphology | Ball et al. [5] |
| *Potentilla clandestina* | W. & SC. China | Alba | molecular analysis | Eriksson et al. [10], Feng et al. [11] |
| *Potentilla clusiana* | C. Europe | Alba | molecular analysis | Dobeš and Paule [8], Töpel et al. [12] |
| *Potentilla crassinervia* | Corse, Sardegna | Alba | molecular analysis | Dobeš and Paule [8] |
| *Potentilla curviseta* | NE. Pakistan to W. Himalaya | Alba | molecular analysis | Dobeš and Paule [8], Eriksson et al. [10] |
| *Potentilla deorum* | Greece | Alba | morphology | Ball et al. [5], Wolf [7] |
| *Potentilla doerfleri* | NW. Balkan Pen. | Alba | morphology | Ball et al. [5] |
| *Potentilla elatior* | E. Türkiye to Caucasus | Alba | molecular analysis | Dobeš and Paule [8] |
| *Potentilla grammopetala* | C. Europe | Alba | molecular analysis | Töpel et al. [12] |
| *Potentilla haynaldiana* | SE. Europe | Alba | morphology | Ball et al. [5], Wolf [7] |
| *Potentilla kionaea* | Greece | Alba | morphology | Ball et al. [5], Wolf [7] |
| *Potentilla libanotica* | Türkiye to Lebanon | Alba | morphology | Wolf [7] |
| *Potentilla montana* | SW. Europe, NW. Africa | Alba | morphology | Ball et al. [5], Wolf [7] |
| *Potentilla nivalis* | France to Spain | Alba | morphology | Ball et al. [5], Wolf [7] |
| *Potentilla omeiensis* | China (C Sichuan) | Alba | molecular analysis | Eriksson et al. [10] |
| *Potentilla oweriniana* | Türkiye | Alba | morphology | Ball et al. [5], Wolf [7] |
| *Potentilla saxifraga* | E. France to NW. Italy | Alba | morphology | Ball et al. [5], Wolf [7] |
| *Potentilla sikkimensis* | Nepal to China (Yunnan) and N. Myanmar | Alba | molecular analysis | Eriksson et al. [10] |
| *Potentilla speciosa* | SE. Europe to Iraq | Alba | morphology | Ball et al. [5], Wolf [7] |
| *Potentilla sterilis* | E. Canada, Europe | Alba | molecular analysis | Töpel et al. [12], Persson et al. [13] |
| *Potentilla tenuis* | Qinghai to China (Sichuan, Gansu) | Alba | molecular analysis | Eriksson et al. [10], Feng et al. [11] |
| *Potentilla valderia* | France to Italy | Alba | molecular analysis | Eriksson et al. [10] |
| *Argentina achillea* | Sumatera | Anserina | morphology | Soják [14] |
| *Argentina adinophylla* | New Guinea | Anserina | morphology | Soják [14] |
| *Argentina archboldiana* | New Guinea | Anserina | morphology | Soják [14] |
| *Argentina aristata* | Nepal to SC. China | Anserina | morphology | Soják [14] |
| *Argentina baliemensis* | W. New Guinea | Anserina | morphology | Soják [14] |
| *Argentina bidentula* | W. New Guinea | Anserina | morphology | Soják [14] |
| *Argentina biloba* | W. New Guinea | Anserina | morphology | Soják [14] |
| *Argentina borneensis* | Sumatera, Borneo | Anserina | morphology | Soják [14] |
| *Argentina brassii* | New Guinea | Anserina | morphology | Soják [14] |
| *Argentina cardotiana* | SC. China to N. Myanmar | Anserina | molecular analysis and morphology | Soják [14], Koski and Ashman [15] |
| *Argentina commutata* | Himalaya | Anserina | morphology | Soják [14] |
| *Argentina contigua* | Nepal to SC. China | Anserina | morphology | Soják [14] |
| *Argentina curta* | Sikkim, China (Tibet) and Burma | Anserina | morphology | Soják [14] |
| *Argentina festiva* | Himalaya to SC. China and N. Myanmar | Anserina | molecular analysis and morphology | Soják [14], Koski and Ashman [15] |
| *Argentina glabriuscula* | Nepal to SC. China and N. Myanmar | Anserina | molecular analysis and morphology | Soják [14], Feng et al. [16] |
| *Argentina gombalana* | China (Sichuan) | Anserina | morphology | Soják [14] |
| *Argentina gorokana* | New Guinea | Anserina | morphology | Soják [14] |
| *Argentina habbemana* | New Guinea | Anserina | morphology | Soják [14] |
| *Argentina hooglandii* | New Guinea | Anserina | morphology | Soják [14] |
| *Argentina indivisa* | New Guinea | Anserina | morphology | Soják [14] |
| *Argentina interrupta* | Nepal to SC. China | Anserina | morphology | Soják [14] |
| *Argentina irianensis* | New Guinea | Anserina | morphology | Soják [14] |
| *Argentina kinabaluensis* | Borneo | Anserina | morphology | Soják [14] |
| *Argentina lignosa* | Türkiye to C. Asia | Anserina | morphology | Soják [14] |
| *Argentina linilaciniata* | New Guinea | Anserina | morphology | Soják [14] |
| *Argentina luteopilosa* | SW China (Tibet, Sichuan, Yunnan) | Anserina | morphology | Soják [14] |
| *Argentina mangenii* | New Guinea | Anserina | morphology | Soják [14] |
| *Argentina microphylla* | Himalaya to SW China (Tibet) and N. Myanmar | Anserina | molecular analysis and morphology | Töpel et al. [12], Soják [14] |
| *Argentina millefoliolata* | China (Yunnan) | Anserina | morphology | Soják [14] |
| *Argentina novoguineensis* | New Guinea | Anserina | morphology | Soják [14] |
| *Argentina papuana* | Philippines to Sulawesi, New Guinea | Anserina | morphology | Soják [14] |
| *Argentina parvula* | N. Borneo, Sulawesi | Anserina | morphology | Soják [14] |
| *Argentina pycnophylla* | Papua New Guinea | Anserina | morphology | Soják [14] |
| *Argentina scorpionis* | Papua New Guinea | Anserina | morphology | Soják [14] |
| *Argentina simulans* | New Guinea | Anserina | morphology | Soják [14] |
| *Argentina sumatrana* | Sumatera | Anserina | morphology | Soják [14] |
| *Argentina tapetodes* | China (Tibet) | Anserina | molecular analysis and morphology | Soják [14], Feng et al. [16] |
| *Argentina tristis* | W. & C. Himalaya | Anserina | morphology | Soják [14] |
| *Argentina tugitakensis* | E China (Taiwan) | Anserina | morphology | Soják [14] |
| *Argentina turfosa* | SW China (Tibet, Yunnan) | Anserina | molecular analysis and morphology | Soják [14], Feng et al. [16] |
| *Argentina victorialis* | Papua New Guinea | Anserina | morphology | Soják [14] |
| *Argentina vittata* | SW China (Tibet, Yunnan) | Anserina | molecular analysis and morphology | Soják [14] |
| *Argentina wanimboi* | W. New Guinea | Anserina | morphology | Soják [14] |
| *Argentina wenchuensis* | China (Sichuan, Guizhou) | Anserina | morphology | Tong and Xia [17] |
| *Argentina wilhelminensis* | New Guinea | Anserina | morphology | Soják [14] |
| *Argentina yonoweana* | W. New Guinea | Anserina | morphology | Soják [14] |
| *Potentilla adenotricha* | S. Siberia | Argentea | morphology | Polozhij and Malyschev [18] |
| *Potentilla agrimonioides* | E. Europe to Mongolia and W. Himalaya | Argentea | molecular analysis | Dobeš and Paule [8] |
| *Potentilla albiflora* | Arizona | Argentea | morphology | Ertter et al. [3] |
| *Potentilla algida* | C. Asia | Argentea | morphology | Soják [19] |
| *Potentilla alpicola* | C. Europe | Argentea | molecular analysis | Dobeš and Paule [8] |
| *Potentilla alsatica* | E. France | Argentea | morphology | Gregor [20] |
| *Potentilla ambigens* | WC. U.S.A. | Argentea | morphology | Ertter et al. [3] |
| *Potentilla anachoretica* | N. Siberia to Alaska | Argentea | morphology | Ertter et al. [3] |
| *Potentilla angelliae* | Utah | Argentea | morphology | Ertter et al. [3] |
| *Potentilla argaea* | Türkiye to N. Iran | Argentea | molecular analysis | Dobeš and Paule [8] |
| *Potentilla argenteiformis* | European Russia | Argentea | morphology | Wolf [7] |
| *Potentilla argyroloma* | Iran | Argentea | morphology | Wolf [7] |
| *Potentilla argyrophylla* | Afghanistan to Himalaya and SW China (Tibet) | Argentea | morphology | Li et al. [2] |
| *Potentilla arizonica* | Arizona | Argentea | molecular analysis | Dobeš and Paule [8] |
| *Potentilla aspegrenii* | Sweden | Argentea | morphology | Kurtto and Eriksson [21] |
| *Potentilla asperrima* | Siberia to Russian Far East and NE. China | Argentea | morphology | Li et al. [2], Juzepczuk [6] |
| *Potentilla assalemica* | Transcaucasus to NW. Iran | Argentea | morphology | Soják [22] |
| *Potentilla asturica* | Iberian Pen. | Argentea | type specimen | Friedrich-Schiller-Universität Jena (JE00001599) |
| *Potentilla aucheriana* | Türkiye to Iran | Argentea | morphology | Wolf [7] |
| *Potentilla balansae* | Türkiye | Argentea | morphology | Faghir et al. [23] |
| *Potentilla beringii* | Kamchatka | Argentea | specimen | Moscow University Herbarium (MW0164187) |
| *Potentilla bipinnatifida* | Subarctic America to N. & WC. U.S.A. | Argentea | morphology | Ertter et al. [3] |
| *Potentilla brevifolia* | NW. U.S.A. to Nevada | Argentea | molecular analysis | Dobeš and Paule [8] |
| *Potentilla buccoana* | Türkiye | Argentea | morphology | Wolf [7] |
| *Potentilla bungei* | S. Transcaucasus to Iran | Argentea | morphology | Juzepczuk [6], Wolf [7] |
| *Potentilla butkovii* | C. Asia | Argentea | specimen | National Herbarium of Uzbekistan (TASH001453) |
| *Potentilla calabra* | SE. Europe to Türkiye | Argentea | morphology | Ball et al. [5] |
| *Potentilla caliginosa* | E. Himalaya to SW China (Tibet) | Argentea | molecular analysis | Ma et al. [24] |
| *Potentilla candicans* | Mexico | Argentea | morphology | Wolf [7] |
| *Potentilla cappadocica* | N. & NE. Türkiye | Argentea | morphology | Wolf [7] |
| *Potentilla chamaeleo* | Mongolia | Argentea | type specimen | Martin-Luther-Universität (HAL0046054) |
| *Potentilla chionea* | S. Siberia | Argentea | morphology | Soják [25] |
| *Potentilla cinerea* | C. & SE. Europe | Argentea | morphology | Ball et al. [5] |
| *Potentilla concinna* | W. Canada to Mexico (Chihuahua) | Argentea | molecular analysis | Dobeš and Paule [8] |
| *Potentilla cottamii* | Nevada to Utah | Argentea | morphology | Ertter et al. [3] |
| *Potentilla crenulata* | China (Yunnan) | Argentea | morphology | Li et al. [2] |
| *Potentilla crinita* | WC. U.S.A. | Argentea | molecular analysis | Dobeš and Paule [8] |
| *Potentilla cristae* | California | Argentea | molecular analysis | Dobeš and Paule [8] |
| *Potentilla cryptophila* | Transcaucasus to Iran | Argentea | morphology | Juzepczuk [6], Wolf [7] |
| *Potentilla darvazica* | C. Asia | Argentea | specimen | Moscow University Herbarium (MW0840138) |
| *Potentilla delphinensis* | France | Argentea | morphology | Ball et al. [5], Wolf [7] |
| *Potentilla demotica* | Arizona | Argentea | morphology | Ertter et al. [3] |
| *Potentilla dentata* | Ethiopia to Kenya, SW. Arabian Pen. | Argentea | type specimen | Museum Botanicum Hauniense, University of Copenhagen (C10002830) |
| *Potentilla desertorum* | Afghanistan to Siberia and W. Himalaya | Argentea | molecular analysis | Dobeš and Paule [8] |
| *Potentilla detommasii* | SE. Europe to W. Türkiye | Argentea | morphology | Ball et al. [5], Wolf [7] |
| *Potentilla divaricata* | Türkiye to Iran | Argentea | morphology | Wolf [7] |
| *Potentilla dombeyi* | Ecuador to Peru | Argentea | morphology | Wolf [7] |
| *Potentilla durangensis* | Mexico (Sinaloa, Durango) | Argentea | type specimen | Royal Botanic Gardens, Kew (K000424965) |
| *Potentilla effusa* | WC. & C. Canada to WC. & NC. U.S.A. | Argentea | molecular analysis | Dobeš and Paule [8] |
| *Potentilla ehrenbergiana* | Mexico (Hidalgo, Veracruz, Oaxaca) | Argentea | morphology | Wolf [7] |
| *Potentilla elvendensis* | SW. Iran | Argentea | morphology | Wolf [7] |
| *Potentilla eversmanniana* | E. European Russia | Argentea | morphology | Ball et al. [5], Juzepczuk [6], Wolf [7] |
| *Potentilla evestita* | E. Europe to Russian Far East and W. Himalaya | Argentea | molecular analysis | Koski and Ashman [15] |
| *Potentilla exuta* | E. Kirgizstan to Altay and Mongolia | Argentea | specimen | Moscow University Herbarium (MW0181826) |
| *Potentilla fedtschenkoana* | C. Asia | Argentea | molecular analysis | Koski and Ashman [15] |
| *Potentilla flabellata* | Afghanistan to C. Asia and N. Pakistan | Argentea | morphology | Juzepczuk [6] |
| *Potentilla fragiformis* | Russian Far East to Alaska | Argentea | morphology | Ertter et al. [3], Juzepczuk [6] |
| *Potentilla frigida* | Europe | Argentea | molecular analysis | Dobeš and Paule [8] |
| *Potentilla furcata* | Alaska to W. Canada | Argentea | morphology | Ertter et al. [3] |
| *Potentilla geranioides* | Türkiye to Anti-Lebanon and Iran | Argentea | morphology | Wolf [7] |
| *Potentilla gerardiana* | Afghanistan to W. Himalaya, Assam (Mizoram) | Argentea | morphology | Landrein et al. [26] |
| *Potentilla glaucescens* | E. Europe to Caucasus and Kazakhstan | Argentea | morphology | Juzepczuk [6] |
| *Potentilla gobica* | Mongolia | Argentea | type specimen | Martin-Luther-Universität (HAL0131621) |
| *Potentilla goldmanii* | Mexico to Guatemala | Argentea | specimen | Missouri Botanical Garden (2851203) |
| *Potentilla granulosa* | SW China (Tibet, Sichuan) to Inner Mongolia | Argentea | morphology | Li et al. [2] |
| *Potentilla grayi* | California | Argentea | morphology | Ertter et al. [3] |
| *Potentilla grisea* | C. Asia to W. Himalaya | Argentea | morphology | Landrein et al. [26] |
| *Potentilla heptaphylla* | Europe to Türkiye | Argentea | morphology | Ball et al. [5], Juzepczuk [6] |
| *Potentilla heterosepala* | Mexico to Guatemala, Colombia | Argentea | morphology | Wolf [7] |
| *Potentilla hickmanii* | California | Argentea | morphology | Ertter et al. [3], Wolf [7] |
| *Potentilla hispanica* | Spain, NW. Africa | Argentea | molecular analysis | Koski and Ashman [15] |
| *Potentilla holmgrenii* | Nevada to Utah | Argentea | morphology | Ertter et al. [3] |
| *Potentilla hololeuca* | Türkiye to NW China (Xinjiang) and W. Himalaya | Argentea | molecular analysis | Koski and Ashman [15] |
| *Potentilla hookeriana* | W. Canada to WC. U.S.A. | Argentea | molecular analysis | Dobeš and Paule [8], Koski and Ashman [15] |
| *Potentilla horrida* | Mexico (Sonora, Chihuahua) | Argentea | morphology | Wolf [7] |
| *Potentilla humillis* | Iran | Argentea | morphology | Mozaffarian [27] |
| *Potentilla ikonnikovii* | Mongolia | Argentea | morphology | Baasanmunkh et al. [28] |
| *Potentilla incana* | Europe to W. Siberia and Caucasus | Argentea | molecular analysis | Dobeš and Paule [8] |
| *Potentilla iranica* | Iran | Argentea | molecular analysis | Faghir et al. [29] |
| *Potentilla jenissejensis* | SW. Siberia to Mongolia | Argentea | molecular analysis | Dobeš and Paule [8] |
| *Potentilla jepsonii* | WC. Canada to NW. & WC. U.S.A. | Argentea | molecular analysis | Dobeš and Paule [8] |
| *Potentilla johanniniana* | Italy | Argentea | morphology | Wolf [7] |
| *Potentilla johnstonii* | Nevada | Argentea | morphology | Ertter et al. [3] |
| *Potentilla khanminczunii* | SW. Siberia | Argentea | molecular analysis | Erst et al. [30] |
| *Potentilla kotschyana* | Türkiye to Lebanon | Argentea | morphology | Mozaffarian [27] |
| *Potentilla kryloviana* | S. Siberia | Argentea | morphology | Polozhij and Malyschev [18] |
| *Potentilla kurdica* | N. Iraq to Iran | Argentea | molecular analysis | Töpel et al. [12], Faghir et al. [29] |
| *Potentilla lasiodonta* | C. Canada to NC. U.S.A. | Argentea | morphology | Ertter et al. [3] |
| *Potentilla lazica* | Türkiye | Argentea | morphology | Wolf [7] |
| *Potentilla leptopetala* | Mexico (Chihuahua, Durango) | Argentea | morphology | Wolf [7] |
| *Potentilla leschenaultiana* | India | Argentea | type specimen | Muséum National d'Histoire Naturelle (P01819143) |
| *Potentilla lignipes* | Bolivia | Argentea | type specimen | The William and Lynda Steere Herbarium of the New York Botanical Garden (NY00415966) |
| *Potentilla lomakinii* | NE. Türkiye to Transcacuasus | Argentea | morphology | Juzepczuk [6] |
| *Potentilla luteosericea* | Mexico (Baja California Norte, Sonora) | Argentea | type specimen | California Academy of Sciences (CAS0004136) |
| *Potentilla macdonaldii* | Mexico (Oaxaca) | Argentea | type specimen | University of Texas at Austin Herbarium (TEX00371080) |
| *Potentilla macounii* | Alberta to Montana | Argentea | morphology | Ertter et al. [3] |
| *Potentilla mallota* | Iran | Argentea | morphology | Wolf [7] |
| *Potentilla maura* | Morocco | Argentea | molecular analysis | Dobeš and Paule [8] |
| *Potentilla mexiae* | N. Mexico | Argentea | type specimen | Field Museum of Natural History, Chicago (F0068267F) |
| *Potentilla meyeri* | Türkiye to Iran | Argentea | molecular analysis | Koski and Ashman [15] |
| *Potentilla mollissima* | C. Asia | Argentea | morphology | Juzepczuk [6], Wolf [7] |
| *Potentilla monanthes* | NE. Pakistan to Himalaya | Argentea | morphology | Wolf [7], Landrein et al. [26] |
| *Potentilla mongolica* | Mongolia | Argentea | morphology | Baasanmunkh et al. [28] |
| *Potentilla montenegrina* | W. & N. Balkan Pen. | Argentea | molecular analysis | Koski and Ashman [15] |
| *Potentilla morefieldii* | California | Argentea | molecular analysis | Koski and Ashman [15] |
| *Potentilla multijuga* | California (Ballona Marsh) | Argentea | morphology | Ertter et al. [3] |
| *Potentilla multisecta* | WC. U.S.A. | Argentea | morphology | Ertter et al. [3] |
| *Potentilla nana* | Alaska to W. Canada | Argentea | morphology | Ertter et al. [3] |
| *Potentilla neglecta* | Europe | Argentea | morphology | Ball et al. [5] |
| *Potentilla nepalensis* | NE. Pakistan to W. & C. Himalaya | Argentea | molecular analysis | Dobeš and Paule [8], Koski and Ashman [15] |
| *Potentilla nervosa* | C. Asia to NW China (Xinjiang) | Argentea | molecular analysis | Koski and Ashman [15] |
| *Potentilla nevadensis* | Spain | Argentea | molecular analysis | Dobeš and Paule [8], Koski and Ashman [15] |
| *Potentilla newberryi* | W. U.S.A. | Argentea | molecular analysis | Dobeš and Paule [8], Koski and Ashman [15] |
| *Potentilla nordmanniana* | Caucasus | Argentea | morphology | Juzepczuk [6] |
| *Potentilla nuda* | Iran | Argentea | morphology | Wolf [7] |
| *Potentilla nurensis* | Iran | Argentea | morphology | Juzepczuk [6], Wolf [7] |
| *Potentilla olchonensis* | S. Siberia | Argentea | morphology | Polozhij and Malyschev [18] |
| *Potentilla omissa* | S. Russian Far East | Argentea | type specimen | Charles University in Prague Herbarium (PRC452358) |
| *Potentilla ovina* | W. & C. Canada to WC. U.S.A. | Argentea | molecular analysis | Dobeš and Paule [8] |
| *Potentilla pannosa* | Türkiye to Afghanistan | Argentea | molecular analysis | Faghir et al. [29] |
| *Potentilla pedata* | C. & SE. Europe to C. Asia and Afghanistan | Argentea | molecular analysis | Dobeš and Paule [8] |
| *Potentilla pedersenii* | Subarctic America | Argentea | molecular analysis | Töpel et al. [12] |
| *Potentilla penniphylla* | SW. Siberia to C. Asia | Argentea | specimen | Moscow University Herbarium (MW0094002) |
| *Potentilla persica* | Iran to Afghanistan | Argentea | molecular analysis | Faghir et al. [29] |
| *Potentilla petraea* | Iran | Argentea | molecular analysis | Faghir et al. [29] |
| *Potentilla petrovskyi* | N. Russian Far East | Argentea | morphology | Soják [31] |
| *Potentilla pindicola* | Balkan Pen. To Krym | Argentea | morphology | Wolf [7] |
| *Potentilla plattensis* | Subarctic America to WC. & NC. U.S.A. | Argentea | molecular analysis | Dobeš and Paule [8] |
| *Potentilla praecox* | C. Europe | Argentea | morphology | Wolf [7] |
| *Potentilla pulcherrima* | Canada to N. & WC. U.S.A. | Argentea | molecular analysis | Dobeš and Paule [8], Koski and Ashman [15] |
| *Potentilla pulvinaris* | Türkiye | Argentea | molecular analysis | Töpel et al. [12] |
| *Potentilla pulviniformis* | N. Siberia to N. Russian Far East | Argentea | specimen | Moscow University Herbarium (MW0955871) |
| *Potentilla pyrenaica* | Pyrenees | Argentea | molecular analysis | Dobeš and Paule [8], Koski and Ashman [15] |
| *Potentilla radiata* | Romania to N. Iran | Argentea | molecular analysis | Faghir et al. [29] |
| *Potentilla ranunculoides* | Mexico | Argentea | molecular analysis | Koski and Ashman [15] |
| *Potentilla reuteri* | S. Spain | Argentea | type specimen | Conservatoire et Jardin botaniques de la Ville de Genève (G00437081) |
| *Potentilla rhenana* | Germany | Argentea | morphology | Wolf [7] |
| *Potentilla rhyolitica* | Arizona | Argentea | molecular analysis | Dobeš and Paule [8] |
| *Potentilla richardii* | Mexico (Jalisco to Veracruz) | Argentea | morphology | Wolf [7] |
| *Potentilla rigidula* | SW. Siberia to Mongolia | Argentea | morphology | Polozhij and Malyschev [18] |
| *Potentilla rigoana* | Italy to NW. Balkan Pen. | Argentea | morphology | Wolf [7] |
| *Potentilla rimicola* | California to Mexico (N. Baja California) | Argentea | morphology | Ertter et al. [3] |
| *Potentilla rivalis* | Canada to N. & W. Mexico | Argentea | molecular analysis | Dobeš and Paule [8], Koski and Ashman [15] |
| *Potentilla robbinsiana* | NE. U.S.A. | Argentea | morphology | Ertter et al. [3], Wolf [7] |
| *Potentilla rubella* | Siberia to N. Russian Far East, Greenland | Argentea | morphology | Ertter et al. [3] |
| *Potentilla rudolfii* | SW. Siberia | Argentea | molecular analysis | Erst et al. [30] |
| *Potentilla rupifraga* | N. Russian Far East | Argentea | specimen | Moscow University Herbarium (MW0093937) |
| *Potentilla rupincola* | Colorado | Argentea | type specimen | Rocky Mountain Herbarium - University of Wyoming (RM0003755) |
| *Potentilla ruprechtii* | Türkiye | Argentea | molecular analysis | Koski and Ashman [15] |
| *Potentilla salsa* | C. Asia | Argentea | specimen | Altai State University (1100000096) |
| *Potentilla sanguisorba* | Siberia to N. Russian Far East and Mongolia | Argentea | morphology | Juzepczuk [6], Wolf [7] |
| *Potentilla saposhnikovii* | Siberia | Argentea | morphology | Polozhij and Malyschev [18] |
| *Potentilla schmakovii* | Mongolia | Argentea | morphology | Kechaykin and Kutsev [32] |
| *Potentilla schrenkiana* | C. Asia | Argentea | morphology | Juzepczuk [6], Wolf [7] |
| *Potentilla sergievskajae* | S. Siberia | Argentea | morphology | Polozhij and Malyschev [18] |
| *Potentilla serrata* | Mongolia | Argentea | type specimen | Swedish Museum of Natural History Department of Botany (S-G-10690) |
| *Potentilla sierrae-blancae* | New Mexico | Argentea | morphology | Ertter et al. [3], Wolf [7] |
| *Potentilla silesiaca* | Poland | Argentea | morphology | Wolf [7] |
| *Potentilla sphenophylla* | NW. Caucasus | Argentea | morphology | Juzepczuk [6], Wolf [7] |
| *Potentilla spodiochlora* | C. & E. Himalaya | Argentea | type specimen | Royal Botanic Garden Edinburgh (E00010756) |
| *Potentilla staminea* | Mexico to Guatemala | Argentea | type specimen | The Peabody Museum of Natural History, Yale University Herbarium (YU066732) |
| *Potentilla subjuga* | WC. Canada to WC. U.S.A. | Argentea | molecular analysis | Dobeš and Paule [8] |
| *Potentilla subpalmata* | Türkiye to S. Transcaucasus | Argentea | morphology | Juzepczuk [6], Wolf [7] |
| *Potentilla subviscosa* | Colorado to Mexico (Sonora) | Argentea | molecular analysis | Dobeš and Paule [8], Koski and Ashman [15] |
| *Potentilla szovitsii* | E. Transcaucasus to N. Iran | Argentea | molecular analysis | Faghir et al. [29] |
| *Potentilla tephroleuca* | Afghanistan to C. Asia and Pakistan | Argentea | morphology | Juzepczuk [6], Wolf [7] |
| *Potentilla tericholica* | SW. Siberia | Argentea | morphology | Polozhij and Malyschev [18] |
| *Potentilla tobolensis* | E. European Russian to W. Siberia, S. Russian Far East | Argentea | morphology | Polozhij and Malyschev [18] |
| *Potentilla tollii* | E. Siberia | Argentea | morphology | Juzepczuk [6], Wolf [7] |
| *Potentilla tommasiniana* | EC. & SE. Europe | Argentea | morphology | Wolf [7] |
| *Potentilla tornezyana* | Morocco | Argentea | type specimen | Muséum National d'Histoire Naturelle (P00124470) |
| *Potentilla townsendii* | Mexico (Sonora, Chihuahua) | Argentea | molecular analysis | Dobeš and Paule [8] |
| *Potentilla tschimganica* | C. Asia | Argentea | type specimen | Botanic Garden and Botanical Museum Berlin-Dahlem, Freie Universität Berlin (B_10_0112628) |
| *Potentilla tschukotica* | NE. Siberia to N. Russian Far East | Argentea | morphology | Polozhij and Malyschev [18] |
| *Potentilla tucumanensis* | N. Argentina | Argentea | morphology | Castagnaro et al. [33] |
| *Potentilla turczaninowiana* | C. Asia to W. Himalaya | Argentea | morphology | Landrein et al. [26] |
| *Potentilla turgaica* | E. Europe to Kazakhstan | Argentea | specimen | Moscow University Herbarium (MW0840773) |
| *Potentilla uliginosa* | California (Cunningham Marsh) | Argentea | morphology | Ertter et al. [3], Wolf [7] |
| *Potentilla umbrosa* | Krym | Argentea | molecular analysis | Dobeš and Paule [8] |
| *Potentilla vahliana* | N. Russian Far East, Subarctic America to NE. Canada | Argentea | molecular analysis | Dobeš and Paule [8] |
| *Potentilla visianii* | W. Balkan Pen. | Argentea | morphology | Ball et al. [5], Wolf [7] |
| *Potentilla volgarica* | E. Europe (Volga River) | Argentea | molecular analysis | Schanzer et al. [34] |
| *Potentilla vulcanicola* | Russian Far East, Subarctic America | Argentea | molecular analysis | Dobeš and Paule [8] |
| *Potentilla vvedenskyi* | C. Asia | Argentea | specimen | National Herbarium of Uzbekistan (TASH001459) |
| *Potentilla wheeleri* | S. California | Argentea | molecular analysis | Dobeš and Paule [8] |
| *Potentilla wimanniana* | Poland | Argentea | type specimen | Friedrich-Schiller-Universität Jena (JE00004009) |
| *Potentilla wrangelii* | N. Russian Far East | Argentea | specimen | Moscow University Herbarium (MW0158876) |
| *Potentilla riparia* | Japan (Honshu, Shikoku) | Fragarioides | molecular analysis | Koski and Ashman [15] |
| *Potentilla squamosa* | S. Korea | Fragarioides | morphology | Heo et al. [35] |
| *Potentilla togasii* | Japan (Honshu) | Fragarioides | morphology | Naruhashi [36] |
| *Potentilla toyamensis* | Japan (Honshu) | Fragarioides | morphology | Naruhashi [36] |
| *Potentilla argyrocoma* | Mexico (Baja California Norte) | Ivesioid | molecular analysis | Töpel et al. [37], Mosyakin et al. [38] |
| *Potentilla baileyi* | California to Nevada | Ivesioid | molecular analysis | Töpel et al. [37], Mosyakin et al. [38] |
| *Potentilla bolanderi* | Mexico (Baja California Norte) | Ivesioid | molecular analysis | Töpel et al. [37], Mosyakin et al. [38] |
| *Potentilla callida* | California | Ivesioid | morphology | Ertter and Reveal [4] |
| *Potentilla campestris* | California | Ivesioid | morphology | Ertter and Reveal [4] |
| *Potentilla clevelandii* | California to NW. Mexico | Ivesioid | morphology | Mosyakin et al. [38] |
| *Potentilla congesta* | W. Oregon | Ivesioid | morphology | Ertter and Reveal [4] |
| *Potentilla cryptocaulis* | Oregon to Nevada | Ivesioid | molecular analysis | Ertter and Reveal [4], Töpel et al. [37] |
| *Potentilla hendersonii* | Oregon to California | Ivesioid | molecular analysis | Ertter and Reveal [4], Töpel et al. [37] |
| *Potentilla howellii* | Oregon to California | Ivesioid | morphology | Ertter and Reveal [4] |
| *Potentilla jaegeri* | California to Nevada | Ivesioid | molecular analysis | Ertter and Reveal [4], Töpel et al. [37] |
| *Potentilla kingii* | E. California to Utah | Ivesioid | molecular analysis | Töpel et al. [37], Mosyakin et al. [38] |
| *Potentilla longibracteata* | California | Ivesioid | molecular analysis | Töpel et al. [37], Mosyakin et al. [38] |
| *Potentilla marinensis* | California | Ivesioid | molecular analysis | Ertter and Reveal [4], Töpel et al. [37] |
| *Potentilla micheneri* | N. California | Ivesioid | morphology | Ertter and Reveal [4] |
| *Potentilla muirii* | California | Ivesioid | morphology | Ertter and Reveal [4] |
| *Potentilla multifoliolata* | Arizona | Ivesioid | morphology | Ertter and Reveal [4] |
| *Potentilla nubigena* | California | Ivesioid | morphology | Ertter and Reveal [4] |
| *Potentilla osterhoutii* | SW. U.S.A. | Ivesioid | morphology | Ertter and Reveal [4] |
| *Potentilla paniculata* | California | Ivesioid | morphology | Mosyakin et al. [38] |
| *Potentilla parryi* | California | Ivesioid | morphology | Ertter and Reveal [4] |
| *Potentilla patellifera* | California | Ivesioid | morphology | Ertter and Reveal [4] |
| *Potentilla pickeringii* | California | Ivesioid | morphology | Ertter and Reveal [4] |
| *Potentilla pityocharis* | Nevada | Ivesioid | morphology | Mosyakin et al. [38] |
| *Potentilla rhypara* | Oregon to Nevada | Ivesioid | morphology | Mosyakin et al. [38] |
| *Potentilla rydbergii* | California | Ivesioid | morphology | Mosyakin et al. [38] |
| *Potentilla sabulosa* | Nevada to Arizona | Ivesioid | molecular analysis | Töpel et al. [37] |
| *Potentilla saxosa* | Mexico (Baja California Norte) | Ivesioid | molecular analysis | Töpel et al. [37], Mosyakin et al. [38] |
| *Potentilla sericata* | Oregon to California | Ivesioid | morphology | Ertter and Reveal [4] |
| *Potentilla sericoleuca* | California | Ivesioid | molecular analysis | Töpel et al. [37] |
| *Potentilla setosa* | Nevada to Utah | Ivesioid | molecular analysis | Töpel et al. [37], Mosyakin et al. [38] |
| *Potentilla shockleyi* | Oregon to Nevada | Ivesioid | molecular analysis | Töpel et al. [37], Mosyakin et al. [38] |
| *Potentilla truncata* | Mexico (Baja California Norte) | Ivesioid | molecular analysis | Töpel et al. [37] |
| *Potentilla tweedyi* | NW. U.S.A. | Ivesioid | molecular analysis | Töpel et al. [37] |
| *Potentilla unguiculata* | California | Ivesioid | molecular analysis | Töpel et al. [37] |
| *Potentilla utahensis* | Utah | Ivesioid | molecular analysis | Töpel et al. [37] |
| *Potentilla wilderae* | California | Ivesioid | molecular analysis | Töpel et al. [37] |
| *Potentilla yadonii* | California | Ivesioid | molecular analysis | Töpel et al. [37], Mosyakin et al. [38] |
| *Potentilla anglica* | Macaronesia, Europe to Siberia and Türkiye | Reptans | morphology | Ertter et al. [3], Ball et al. [5], Wolf [7] |
| *Potentilla simplex* | E. Canada to C. & E. U.S.A. | Reptans | molecular analysis | Dobeš and Paule [8] |

**Table S3** Sample fraction of *Potentilla* in global and the five geographic regions.

|  | Total | Species with plastome (sample fraction) | Species with distribution data (sample fraction) |
| --- | --- | --- | --- |
| Global | 503 | 149 (0.30) | 451 (0.90) |
| North America | 153 | 43 (0.28) | 142 (0.93) |
| Europe | 146 | 47 (0.32) | 130 (0.89) |
| North Asia | 127 | 48 (0.38) | 114 (0.90) |
| Qinghai-Tibet Plateau | 91 | 42 (0.46) | 72 (0.79) |
| East Asia | 52 | 42 (0.81) | 48 (0.92) |

**Table S4** Biogeographic models tested in this study with estimated parameters from BioGeoBEARS analyses, with the best model denoted in bold.

| Model | LnL | numparams | d | e | j | AICc | AICcwt |
| --- | --- | --- | --- | --- | --- | --- | --- |
| DEC | -347.7 | 2 | 0.14 | 0.62 | 0 | 699.4 | 5.20E-09 |
| DEC+J | -347.2 | 3 | 0.13 | 0.58 | 0.0059 | 700.6 | 2.80E-09 |
| DIVALIKE | -358 | 2 | 0.17 | 0.84 | 0 | 720 | 1.70E-13 |
| DIVALIKE+J | -357.9 | 3 | 0.17 | 0.86 | 0.0021 | 721.9 | 6.60E-14 |
| BAYAREALIKE | -339.6 | 2 | 0.04 | 0.37 | 0 | 683.4 | 1.60E-05 |
| **BAYAREALIKE+J** | **-327.5** | **3** | **0.055** | **0.45** | **0.0078** | **661.2** | **1** |

**Table S5** RPANDA diversification models fit of paleotemperature-dependence. logL: log-likelihood; AICc: corrected Akaike Information Criterion; λ_0_: speciation rates for a given environmental variable; α: parameter controlling variation of speciation with paleo-environment; μ_0_: extinction rates for a given environmental variable. β: parameter controlling variation of extinction with paleo-environment; bcst: birth with a constant speciation rate; bexp: birth with an exponential speciation rate; blin: birth with a linear speciation rate; dcst: death with a constant extinction rate; dexp: death with an exponential extinction rate; dlin: death with a linear extinction rate.

| **Models** | **logL** | **AICc** | **λ_0_** | **α** | **μ_0_** | **β** |
| --- | --- | --- | --- | --- | --- | --- |
| bcst_dcst | -318.927 | 641.933 | 0.637 | NA | 0.564 | NA |
| bexp_dcst | -316.963 | 640.086 | 0.956 | -0.184 | 0.051 | NA |
| bcst_dexp | -317.742 | 641.644 | 0.581 | NA | 0.330 | 0.056 |
| bexp_dexp | -316.877 | 642.023 | 0.928 | -0.177 | 0.077 | 0.058 |
| **blin_dcst** | **-315.723** | **637.606** | **0.724** | **-0.055** | **0.181** | **NA** |
| bcst_dlin | -317.446 | 641.051 | 0.557 | NA | 0.207 | 0.036 |
| blin_dlin | -316.253 | 640.775 | -0.190 | 0.251 | -0.269 | 0.258 |

**Table S6** The traits coding used in the trait-dependent diversification rate analyses. Ploidy: (1) Diploidy, (2) Polyploidy; Leaves hair: (1) Densely hairy, (2) Sparsely hairy; Root: (1) Robust with less branched, (2) Slender with much branched; Basal leaves: (1) Ternate, (2) Palmate (≥ 5), (3) Pinnate (≤ 10pairs), (4) Pinnate (> 10pairs). “-” not available.

| Latename | Ploidy | Abaxial leaflet | Roots | Basal leaves |
| --- | --- | --- | --- | --- |
| *Argentina anserina* | 2 | 1 | 2 | 3 |
| *Argentina fallens* | 2 | 1 | 2 | 4 |
| *Argentina leuconota* | 1 | 1 | 2 | 4 |
| *Argentina lineata* | 1 | 1 | 2 | 3 |
| *Argentina micropetala* | 1 | 1 | 2 | 3 |
| *Argentina peduncularis* | 2 | 1 | 2 | 4 |
| *Argentina phanerophlebia* | - | 1 | 1 | 3 |
| *Argentina polyphylla* | 2 | 2 | 2 | 3 |
| *Argentina smithiana* | - | 2 | 2 | 3 |
| *Argentina stenophylla* | - | 2 | 2 | 4 |
| *Argentina taliensis* | - | 2 | 2 | 4 |
| *Argentina tatsienluensis* | - | 2 | 2 | 4 |
| *Potentilla californica* | 2 | 2 | 2 | 3 |
| *Potentilla lindleyi* | 2 | 2 | 2 | 3 |
| *Potentilla daucifolia* | - | 1 | 2 | 3 |
| *Potentilla douglasii* | 2 | 2 | 2 | 3 |
| *Potentilla hispidula* | - | 1 | 2 | 4 |
| *Potentilla tilingii* | 2 | 1 | 2 | 3 |
| *Potentilla tularensis* | - | 1 | 2 | 3 |
| *Potentilla purpurascens* | - | 1 | 2 | 4 |
| *Potentilla aperta* | - | 1 | 2 | 4 |
| *Potentilla gordonii* | - | 2 | 2 | 4 |
| *Potentilla lycopodioides* | - | 2 | 2 | 4 |
| *Potentilla santolinoides* | 2 | 1 | 2 | 4 |
| *Potentilla webberi* | - | 1 | 2 | 3 |
| *Potentilla acaulis* | 1 | 1 | 1 | 1 |
| *Potentilla alba* | 2 | 1 | 2 | 2 |
| *Potentilla ancistrifolia* | 1 | 1 | 2 | 3 |
| *Potentilla angustiloba* | - | 1 | 2 | 2 |
| *Potentilla anjuica* | 2 | 1 | 2 | 1 |
| *Potentilla approximata* | 2 | 1 | 2 | 3 |
| *Potentilla arenosa* | 2 | 1 | 2 | 1 |
| *Potentilla argentea* | 2 | 1 | 2 | 2 |
| *Potentilla articulata* | - | 2 | 2 | 1 |
| *Potentilla astracanica* | 2 | 1 | 2 | 2 |
| *Potentilla aurea* | 1 | 2 | 2 | 2 |
| *Potentilla basaltica* | - | 2 | 2 | 4 |
| *Potentilla betonicifolia* | - | 1 | 2 | 1 |
| *Potentilla biennis* | - | 2 | 2 | 1 |
| *Potentilla biflora* | 1 | 2 | 2 | 1 |
| *Potentilla brachypetala* | - | 2 | 2 | 2 |
| *Potentilla brauneana* | 1 | 2 | 2 | 1 |
| *Potentilla brunnescens* | - | 2 | 2 | 2 |
| *Potentilla canadensis* | 2 | 1 | 1 | 2 |
| *Potentilla caulescens* | 1 | 2 | 2 | 2 |
| *Potentilla centigrana* | 1 | 2 | 1 | 1 |
| *Potentilla chamissonis* | 2 | 1 | 2 | 1 |
| *Potentilla chinensis* | 1 | 1 | 2 | 3 |
| *Potentilla chrysantha* | 2 | 2 | 2 | 2 |
| *Potentilla collina* | 2 | 1 | 2 | 2 |
| *Potentilla conferta* | 2 | 1 | 2 | 3 |
| *Potentilla coriandrifolia* | 2 | 2 | 2 | 3 |
| *Potentilla coriandrifolia* var. *dumosa* | 2 | 2 | 2 | 3 |
| *Potentilla crantzii* | 2 | 2 | 1 | 1 |
| *Potentilla cryptotaeniae* | 1 | 2 | 1 | 1 |
| *Potentilla delavayi* | - | 1 | 2 | 1 |
| *Potentilla dickinsii* | 1 | 2 | 2 | 3 |
| *Potentilla dickinsii* var. *glabrata* | - | 2 | 2 | 3 |
| *Potentilla discolor* | - | 1 | 2 | 3 |
| *Potentilla divina* | - | 1 | 2 | 1 |
| *Potentilla doubjonneana* | - | 2 | 2 | 1 |
| *Potentilla drummondii* | 2 | 2 | 2 | 3 |
| *Potentilla elegans* | 1 | 2 | 2 | 1 |
| *Potentilla erecta* | 2 | 2 | 2 | 1 |
| *Potentilla eriocarpa* | 1 | 2 | 2 | 1 |
| *Potentilla flabellifolia* | 2 | 2 | 2 | 1 |
| *Potentilla flagellaris* | 1 | 2 | 1 | 1 |
| *Potentilla fragarioides* | 1 | 2 | 1 | 3 |
| *Potentilla freyniana* | 1 | 2 | 1 | 1 |
| *Potentilla gageodoensis* | - | 2 | 1 | 3 |
| *Potentilla gelida* | 2 | 2 | 1 | 1 |
| *Potentilla glaucophylla* | 2 | 2 | 2 | 2 |
| *Potentilla gracilis* | 2 | 1 | 2 | 2 |
| *Potentilla grandiflora* | 2 | 2 | 2 | 1 |
| *Potentilla griffithii* | - | 1 | 2 | 3 |
| *Potentilla hebiichigo* | 2 | 2 | 1 | 1 |
| *Potentilla hippiana* | 2 | 1 | 2 | 3 |
| *Potentilla hirta* | 2 | 1 | 2 | 2 |
| *Potentilla humifusa* | 2 | 2 | 2 | 2 |
| *Potentilla hyparctica* | 2 | 1 | 2 | 1 |
| *Potentilla hypargyrea* | - | 1 | 2 | 1 |
| *Potentilla hypargyrea* var. *subpinnata* | - | 1 | 2 | 1 |
| *Potentilla impolita* | 2 | 1 | 2 | 2 |
| *Potentilla indica* | 2 | 2 | 1 | 1 |
| *Potentilla intermedia* | 2 | 1 | 2 | 2 |
| *Potentilla kleiniana* | 1 | 2 | 1 | 2 |
| *Potentilla lancinata* | - | 2 | 2 | 3 |
| *Potentilla leucopolitana* | 1 | 1 | 2 | 2 |
| *Potentilla limprichtii* | - | 1 | 2 | 3 |
| *Potentilla longifolia* | 1 | 2 | 2 | 3 |
| *Potentilla longipes* | - | 2 | 2 | 2 |
| *Potentilla lyngei* | 2 | 1 | 2 | 3 |
| *Potentilla macrosepala* | - | 2 | 2 | 1 |
| *Potentilla matsumurae* | 2 | 2 | 2 | 1 |
| *Potentilla megalantha* | 2 | 1 | 2 | 1 |
| *Potentilla micrantha* | 1 | 2 | 2 | 1 |
| *Potentilla millefolia* | - | 2 | 2 | 3 |
| *Potentilla multicaulis* | - | 1 | 2 | 3 |
| *Potentilla multiceps* | - | 1 | 2 | 3 |
| *Potentilla multifida* | 2 | 1 | 2 | 3 |
| *Potentilla nitida* | 2 | 1 | 2 | 1 |
| *Potentilla nivea* | 2 | 1 | 2 | 1 |
| *Potentilla norvegica* | 2 | 2 | 2 | 1 |
| *Potentilla opizii* | - | 2 | 2 | 2 |
| *Potentilla pamirica* | 2 | 1 | 2 | 3 |
| *Potentilla pamiroalaica* | 2 | 1 | 2 | 3 |
| *Potentilla patula* | 2 | 1 | 2 | 2 |
| *Potentilla pendula* | - | 1 | 2 | 1 |
| *Potentilla pensylvanica* | 2 | 2 | 2 | 3 |
| *Potentilla peterae* | - | 1 | 2 | 3 |
| *Potentilla pimpinelloides* | 1 | 2 | 2 | 3 |
| *Potentilla plumosa* | - | 1 | 2 | 3 |
| *Potentilla potaninii* var. *compsophylla* | - | 1 | 2 | 3 |
| *Potentilla potaninii* | - | 1 | 2 | 3 |
| *Potentilla pseudosericea* | - | 1 | 2 | 3 |
| *Potentilla pseudosimulatrix* | - | 2 | 1 | 1 |
| *Potentilla pulchella* | 2 | 1 | 2 | 3 |
| *Potentilla purpurea* | 2 | 2 | 2 | 2 |
| *Potentilla pusilla* | 2 | 2 | 2 | 2 |
| *Potentilla recta* subsp. *obscura* | - | 1 | 2 | 2 |
| *Potentilla recta* | 2 | 1 | 2 | 2 |
| *Potentilla reptans* | 2 | 2 | 1 | 2 |
| *Potentilla hemsleyana* | - | 2 | 1 | 1 |
| *Potentilla rosulifera* | 1 | 2 | 1 | 1 |
| *Potentilla rubricaulis* | 2 | 1 | 2 | 2 |
| *Potentilla sinonivea* | - | 1 | 2 | 1 |
| *Potentilla forrestii* | - | 1 | 2 | 1 |
| *Potentilla saundersiana* | - | 1 | 2 | 2 |
| *Potentilla sericea* | 2 | 1 | 2 | 3 |
| *Potentilla simulatrix* | - | 2 | 1 | 1 |
| *Potentilla sischanensis* | - | 1 | 2 | 3 |
| *Potentilla soongorica* | - | 1 | 2 | 3 |
| *Potentilla stipularis* | 2 | 2 | 2 | 2 |
| *Potentilla stolonifera* | 1 | 2 | 1 | 3 |
| *Potentilla strigosa* | - | 2 | 2 | 3 |
| *Potentilla suavis* | - | 2 | 2 | 2 |
| *Potentilla subarenaria* | 2 | 2 | 2 | 2 |
| *Potentilla subdigitata* | - | 1 | 2 | 1 |
| *Potentilla subgorodkovii* | 2 | 1 | 2 | 1 |
| *Potentilla subvahliana* | 2 | 1 | 2 | 1 |
| *Potentilla supina* | 2 | 2 | 2 | 3 |
| *Potentilla tabernaemontani* | 2 | 2 | 2 | 2 |
| *Potentilla tanacetifolia* | 2 | 2 | 2 | 3 |
| *Potentilla taurica* | - | 1 | 2 | 2 |
| *Potentilla tetrandra* | - | 2 | 2 | 1 |
| *Potentilla thurberi* | - | 1 | 2 | 2 |
| *Potentilla thuringiaca* | 2 | 2 | 2 | 2 |
| *Potentilla thyrsiflora* | 2 | 2 | 2 | 2 |
| *Potentilla uniflora* | 2 | 1 | 2 | 1 |
| *Potentilla verna* | 2 | 2 | 2 | 2 |
| *Potentilla verticillaris* | - | 1 | 2 | 3 |
| *Potentilla villosa* | 1 | 1 | 2 | 1 |
| *Potentilla virgata* | 1 | 1 | 2 | 2 |
| *Potentilla xizangensis* | - | 2 | 2 | 1 |

**Table S7** HiSSE models used in this study. Abbreviations: HiSSE, Hidden State Speciation and Extinction; BiSSE, Binary State Speciation and Extinction; CID, character-independent diversification; τ, net turnover; ε, extinction fraction; q, transition rates between both the observed trait and the hidden trait.

| Model number | Model name |
| --- | --- |
| 1 | HiSSE full model |
| 2 | BiSSE: all free |
| 3 | BiSSE: ε0=ε1 |
| 4 | BiSSE: q’s equal |
| 5 | BiSSE: ε0=ε1, q’s equal |
| 6 | CID-2: q’s equal |
| 7 | CID-2: ε’s, q’s equal |
| 8 | CID-4: q’s equal |
| 9 | CID-4: ε’s equal, q’s equal |
| 10 | HiSSE: q’s equal |
| 11 | HiSSE: ε’s equal, q’s equal |
| 12 | HiSSE: τ0A=τ1A=τ0B, ε0A=ε1A=ε0B, q’s equal |
| 13 | HiSSE: τ0A=τ1A=τ0B, ε’s equal, q’s equal |
| 14 | HiSSE: q0B1B=0, q1B0B=0, all other q’s equal |
| 15 | HiSSE: ε’s equal, q0B1B=0, q1B0B=0, all other q’s equal |
| 16 | HiSSE: τ0A=τ1A=τ0B, ε0A=ε1A=ε0B, q0B1B=0, q1B0B=0, all other q’s equal |
| 17 | HiSSE: τ0A=τ1A=τ0B, ε’s equal, q0B1B=0, q1B0B=0, all other q’s equal |
| 18 | HiSSE: τ0A=τ0B, ε0A=ε0B, q’s equal |
| 19 | HiSSE: τ0A=τ0B, ε’s equal, q’s equal |
| 20 | HiSSE: τ0A=τ0B, ε0A=ε0B, q0B1B=0, q1B0B=0, all other q’s equal |
| 21 | HiSSE: τ0A=τ0B, ε’s equal, q0B1B=0, q1B0B=0, all other q’s equal |
| 22 | HiSSE: τ0A=τ1A, ε0A=ε1A, q’s equal |
| 23 | HiSSE: τ0A=τ1A, ε’s equal, q’s equal |
| 24 | HiSSE: τ0A=τ1A, ε0A=ε1A, q0B1B=0, q1B0B=0, all other q’s equa |
| 25 | HiSSE: τ0A=τ1A, ε’s equal, q0B1B=0, q1B0B=0, all other q’s equal |

**Table S8** Model comparison of HiSSE analysis for the three binary traits. For each HiSSE model, the maximum negative log-likelihood (Loglik), corrected Akaike's information criterion (AICc) value and the difference between the best model and each model (ΔAICc) are provided, with the best model denoted in bold. For simplicity, just model number is provided, the corresponding model name is provided in Table S7.

| Model  number | Ploidy | | | Leaves hair | | | Roots | | |
| --- | --- | --- | --- | --- | --- | --- | --- | --- | --- |
|  | Loglik | AICc | ΔAICc | Loglik | AICc | ΔAICc | Loglik | AICc | ΔAICc |
| **1** | **-295.431** | **622.863** | **0.000** | **-357.181** | **746.362** | **0.000** | **-295.431** | **622.863** | **0.000** |
| 2 | -351.912 | 715.824 | 92.961 | -392.746 | 797.492 | 51.130 | -351.912 | 715.824 | 92.961 |
| 3 | -352.129 | 714.259 | 91.396 | -392.746 | 795.492 | 49.130 | -352.129 | 714.259 | 91.396 |
| 4 | -352.016 | 714.033 | 91.170 | -396.888 | 803.775 | 57.413 | -352.016 | 714.033 | 91.170 |
| 5 | -353.567 | 715.133 | 92.271 | -397.292 | 802.584 | 56.222 | -353.567 | 715.133 | 92.271 |
| 6 | -311.614 | 633.228 | 10.365 | -382.105 | 774.209 | 27.847 | -311.614 | 633.228 | 10.365 |
| 7 | -311.614 | 631.228 | 8.365 | -387.018 | 782.036 | 35.674 | -311.614 | 631.228 | 8.365 |
| 8 | -311.771 | 641.542 | 18.679 | -408.014 | 834.028 | 87.666 | -311.771 | 641.542 | 18.679 |
| 9 | -402.735 | 817.470 | 194.608 | -400.721 | 813.443 | 67.081 | -402.735 | 817.470 | 194.608 |
| 10 | -307.728 | 633.455 | 10.593 | -379.495 | 776.990 | 30.628 | -307.728 | 633.455 | 10.593 |
| 11 | -310.030 | 632.060 | 9.197 | -378.447 | 768.895 | 22.533 | -310.030 | 632.060 | 9.197 |
| 12 | -351.862 | 713.724 | 90.861 | -381.482 | 772.964 | 26.602 | -351.862 | 713.724 | 90.861 |
| 13 | -353.433 | 714.866 | 92.003 | -381.890 | 771.779 | 25.417 | -353.433 | 714.866 | 92.003 |
| 14 | -350.312 | 714.624 | 91.762 | -381.046 | 776.093 | 29.731 | -350.312 | 714.624 | 91.762 |
| 15 | -353.567 | 717.133 | 94.271 | -381.561 | 773.122 | 26.760 | -353.567 | 717.133 | 94.271 |
| 16 | -311.050 | 636.100 | 13.238 | -375.817 | 765.633 | 19.271 | -311.050 | 636.100 | 13.238 |
| 17 | -311.050 | 632.100 | 9.238 | -379.193 | 768.386 | 22.024 | -311.050 | 632.100 | 9.238 |
| 18 | -316.949 | 651.897 | 29.035 | -378.260 | 774.519 | 28.157 | -316.949 | 651.897 | 29.035 |
| 19 | -318.585 | 649.171 | 26.308 | -378.733 | 769.465 | 23.103 | -318.585 | 649.171 | 26.308 |
| 20 | -351.915 | 713.830 | 90.968 | -415.690 | 841.381 | 95.019 | -351.915 | 713.830 | 90.968 |
| 21 | -352.296 | 712.591 | 89.728 | -415.690 | 839.380 | 93.018 | -352.296 | 712.591 | 89.728 |
| 22 | -350.840 | 715.679 | 92.817 | -380.624 | 775.249 | 28.887 | -350.840 | 715.679 | 92.817 |
| 23 | -351.082 | 712.165 | 89.302 | -380.912 | 771.824 | 25.462 | -351.082 | 712.165 | 89.302 |
| 24 | -325.914 | 665.828 | 42.965 | -386.563 | 787.125 | 40.763 | -325.914 | 665.828 | 42.965 |
| 25 | -330.385 | 670.769 | 47.907 | -380.957 | 771.914 | 25.552 | -330.385 | 670.769 | 47.907 |

**Table S9** MuSSE models and model selection for the relationship between basal leaves type and diversification rates. Df: degree of freedom; lnLik: log-likelihood; AICc: corrected Akaike Information Criterion; ΔAICc: the difference in AICc between the model with the lowest AICc; Pr(>|Chi|): p-value of Chi-Square test.

| Model | Df | lnLik | AICc | ΔAICc | ChiSq | Pr(>\|Chi\|) |
| --- | --- | --- | --- | --- | --- | --- |
| null model | 14 | -486.788 | 1001.576 | 70.860 | NA | NA |
| **full model** | **20** | **-445.358** | **930.716** | **0.000** | **82.860** | **8.88E-16** |
| free λ | 17 | -455.854 | 945.708 | 14.991 | 61.869 | 2.34E-13 |
| free μ | 17 | -464.774 | 963.548 | 32.832 | 44.028 | 1.49E-09 |

**Table S10** The divergence time and tip diversification rate of each species calculated based on the empirical time-calibrated tree.

| **Species** | **Diversification rate (species/Ma)** | **Divergence time (Ma)** |
| --- | --- | --- |
| *Argentina anserina* | 0.049 | 11.686 |
| *Argentina fallens* | 0.094 | 3.127 |
| *Argentina leuconota* | 0.094 | 3.127 |
| *Argentina lineata* | 0.068 | 11.554 |
| *Argentina micropetala* | 0.063 | 8.240 |
| *Argentina peduncularis* | 0.097 | 2.364 |
| *Argentina phanerophlebia* | 0.063 | 8.240 |
| *Argentina polyphylla* | 0.068 | 11.554 |
| *Argentina smithiana* | 0.049 | 11.686 |
| *Argentina stenophylla* | 0.097 | 2.364 |
| *Argentina taliensis* | 0.090 | 4.336 |
| *Argentina tatsienluensis* | 0.090 | 4.336 |
| *Potentilla californica* | 0.373 | 1.687 |
| *Potentilla lindleyi* | 0.566 | 0.851 |
| *Potentilla daucifolia* | 1.352 | 0.155 |
| *Potentilla douglasii* | 1.352 | 0.155 |
| *Potentilla hispidula* | 0.360 | 1.876 |
| *Potentilla tilingii* | 0.755 | 1.158 |
| *Potentilla tularensis* | 0.937 | 0.367 |
| *Potentilla purpurascens* | 0.937 | 0.367 |
| *Potentilla aperta* | 0.670 | 1.201 |
| *Potentilla gordonii* | 0.452 | 1.650 |
| *Potentilla lycopodioides* | 0.184 | 2.893 |
| *Potentilla santolinoides* | 0.923 | 0.385 |
| *Potentilla webberi* | 0.923 | 0.385 |
| *Potentilla acaulis* | 1.285 | 0.379 |
| *Potentilla alba* | 0.095 | 8.806 |
| *Potentilla ancistrifolia* | 0.197 | 1.124 |
| *Potentilla angustiloba* | 0.224 | 2.504 |
| *Potentilla anjuica* | 0.931 | 0.200 |
| *Potentilla approximata* | 1.882 | 0.237 |
| *Potentilla arenosa* | 0.751 | 1.148 |
| *Potentilla argentea* | 3.090 | 0.046 |
| *Potentilla articulata* | 0.111 | 2.502 |
| *Potentilla astracanica* | 1.742 | 0.441 |
| *Potentilla aurea* | 0.858 | 1.058 |
| *Potentilla basaltica* | 1.622 | 0.375 |
| *Potentilla betonicifolia* | 1.882 | 0.237 |
| *Potentilla biennis* | 0.125 | 6.176 |
| *Potentilla biflora* | 0.111 | 2.502 |
| *Potentilla brachypetala* | 0.151 | 2.682 |
| *Potentilla brauneana* | 0.480 | 1.985 |
| *Potentilla brunnescens* | 1.038 | 0.788 |
| *Potentilla canadensis* | 0.191 | 3.553 |
| *Potentilla caulescens* | 0.236 | 1.861 |
| *Potentilla centigrana* | 0.036 | 18.390 |
| *Potentilla chamissonis* | 1.809 | 0.210 |
| *Potentilla chinensis* | 1.214 | 0.475 |
| *Potentilla chrysantha* | 1.285 | 0.379 |
| *Potentilla collina* | 1.664 | 0.138 |
| *Potentilla conferta* | 0.853 | 0.586 |
| *Potentilla coriandrifolia* | 0.115 | 5.940 |
| *Potentilla coriandrifolia* var. *dumosa* | 0.189 | 1.902 |
| *Potentilla crantzii* | 0.745 | 1.238 |
| *Potentilla cryptotaeniae* | 0.288 | 2.473 |
| *Potentilla delavayi* | 0.572 | 1.297 |
| *Potentilla dickinsii* | 0.111 | 3.036 |
| *Potentilla dickinsii* var. *glabrata* | 0.197 | 1.124 |
| *Potentilla discolor* | 0.867 | 0.295 |
| *Potentilla divina* | 0.110 | 5.946 |
| *Potentilla doubjonneana* | 0.480 | 1.985 |
| *Potentilla drummondii* | 2.267 | 0.266 |
| *Potentilla elegans* | 0.155 | 3.083 |
| *Potentilla erecta* | 0.191 | 3.553 |
| *Potentilla eriocarpa* | 0.088 | 9.079 |
| *Potentilla flabellifolia* | 0.653 | 1.351 |
| *Potentilla flagellaris* | 0.168 | 2.585 |
| *Potentilla fragarioides* | 1.392 | 0.213 |
| *Potentilla freyniana* | 0.817 | 0.338 |
| *Potentilla gageodoensis* | 1.392 | 0.213 |
| *Potentilla gelida* | 0.513 | 1.827 |
| *Potentilla glaucophylla* | 1.051 | 0.764 |
| *Potentilla gracilis* | 1.166 | 0.753 |
| *Potentilla grandiflora* | 0.871 | 1.022 |
| *Potentilla griffithii* | 0.542 | 1.414 |
| *Potentilla hebiichigo* | 0.168 | 2.585 |
| *Potentilla hippiana* | 0.556 | 1.479 |
| *Potentilla hirta* | 0.824 | 1.088 |
| *Potentilla humifusa* | 1.742 | 0.441 |
| *Potentilla hyparctica* | 0.931 | 0.200 |
| *Potentilla hypargyrea* | 0.327 | 0.824 |
| *Potentilla hypargyrea* var. *subpinnata* | 0.327 | 0.824 |
| *Potentilla impolita* | 1.491 | 0.277 |
| *Potentilla indica* | 0.151 | 0.618 |
| *Potentilla intermedia* | 0.847 | 0.246 |
| *Potentilla kleiniana* | 0.422 | 2.109 |
| *Potentilla lancinata* | 0.824 | 1.088 |
| *Potentilla leucopolitana* | 1.050 | 0.739 |
| *Potentilla limprichtii* | 0.867 | 0.295 |
| *Potentilla longifolia* | 0.774 | 0.558 |
| *Potentilla longipes* | 3.090 | 0.046 |
| *Potentilla lyngei* | 1.809 | 0.210 |
| *Potentilla macrosepala* | 0.572 | 1.297 |
| *Potentilla matsumurae* | 0.537 | 1.701 |
| *Potentilla megalantha* | 1.642 | 0.014 |
| *Potentilla micrantha* | 0.110 | 5.946 |
| *Potentilla millefolia* | 2.267 | 0.266 |
| *Potentilla multicaulis* | 0.787 | 1.097 |
| *Potentilla multiceps* | 0.751 | 1.131 |
| *Potentilla multifida* | 0.787 | 1.097 |
| *Potentilla nitida* | 0.236 | 1.861 |
| *Potentilla nivea* | 0.729 | 0.898 |
| *Potentilla norvegica* | 0.847 | 0.246 |
| *Potentilla opizii* | 3.367 | 0.173 |
| *Potentilla pamirica* | 0.978 | 0.961 |
| *Potentilla pamiroalaica* | 0.831 | 0.891 |
| *Potentilla pendula* | 0.902 | 0.813 |
| *Potentilla pensylvanica* | 0.911 | 0.482 |
| *Potentilla peterae* | 0.581 | 1.598 |
| *Potentilla pimpinelloides* | 0.651 | 1.376 |
| *Potentilla plumosa* | 0.491 | 1.870 |
| *Potentilla potaninii* var. *compsophylla* | 1.925 | 0.231 |
| *Potentilla potaninii* | 1.238 | 0.507 |
| *Potentilla pseudosericea* | 1.051 | 0.764 |
| *Potentilla pseudosimulatrix* | 0.151 | 1.580 |
| *Potentilla pulchella* | 0.923 | 0.989 |
| *Potentilla purpurea* | 0.126 | 2.943 |
| *Potentilla pusilla* | 1.151 | 0.566 |
| *Potentilla recta* subsp. *obscura* | 1.991 | 0.297 |
| *Potentilla recta* | 1.991 | 0.297 |
| *Potentilla reptans* | 0.145 | 4.509 |
| *Potentilla hemsleyana* | 0.280 | 0.513 |
| *Potentilla rosulifera* | 0.259 | 1.101 |
| *Potentilla rubricaulis* | 1.117 | 0.448 |
| *Potentilla sinonivea* | 1.925 | 0.231 |
| *Potentilla forrestii* | 0.632 | 1.442 |
| *Potentilla saundersiana* | 0.712 | 0.965 |
| *Potentilla schurii* | 1.151 | 0.566 |
| *Potentilla sericea* | 0.874 | 0.957 |
| *Potentilla simulatrix* | 0.280 | 0.513 |
| *Potentilla sischanensis* | 1.212 | 0.360 |
| *Potentilla soongorica* | 1.642 | 0.014 |
| *Potentilla stipularis* | 0.729 | 0.898 |
| *Potentilla stolonifera* | 0.474 | 0.359 |
| *Potentilla strigosa* | 0.911 | 0.482 |
| *Potentilla suavis* | 0.077 | 7.381 |
| *Potentilla subarenaria* | 1.580 | 0.244 |
| *Potentilla subdigitata* | 0.632 | 1.442 |
| *Potentilla subgorodkovii* | 1.069 | 0.407 |
| *Potentilla subvahliana* | 0.878 | 0.815 |
| *Potentilla supina* | 0.457 | 2.032 |
| *Potentilla tabernaemontani* | 0.854 | 1.000 |
| *Potentilla tanacetifolia* | 1.214 | 0.475 |
| *Potentilla taurica* | 0.871 | 1.022 |
| *Potentilla tetrandra* | 0.126 | 2.943 |
| *Potentilla thurberi* | 0.556 | 1.479 |
| *Potentilla thuringiaca* | 1.491 | 0.277 |
| *Potentilla thyrsiflora* | 2.374 | 0.209 |
| *Potentilla uniflora* | 1.069 | 0.407 |
| *Potentilla verna* | 3.367 | 0.173 |
| *Potentilla verticillaris* | 0.692 | 1.130 |
| *Potentilla villosa* | 0.878 | 0.815 |
| *Potentilla virgata* | 0.874 | 0.957 |
| *Potentilla xizangensis* | 0.426 | 2.234 |

**Table S11** The divergence time and tip diversification rate of each species calculated based on the simulated time-calibrated tree.

| **Species** | **Diversification rate (species/Ma)** | **Divergence time (Ma)** |
| --- | --- | --- |
| *Argentina achillea* | 0.155 | 1.896 |
| *Argentina adinophylla* | 0.073 | 11.167 |
| *Argentina anserina* | 0.092 | 6.121 |
| *Argentina archboldiana* | 0.168 | 2.023 |
| *Argentina aristata* | 0.152 | 0.432 |
| *Argentina baliemensis* | 0.051 | 15.762 |
| *Argentina bidentula* | 0.058 | 15.029 |
| *Argentina biloba* | 0.219 | 0.705 |
| *Argentina borneensis* | 0.196 | 2.470 |
| *Argentina brassii* | 0.217 | 2.501 |
| *Argentina cardotiana* | 0.118 | 4.254 |
| *Argentina commutata* | 0.149 | 2.756 |
| *Argentina contigua* | 0.174 | 0.443 |
| *Argentina curta* | 0.148 | 0.846 |
| *Argentina fallens* | 0.234 | 0.399 |
| *Argentina festiva* | 0.168 | 2.023 |
| *Argentina glabriuscula* | 0.096 | 6.256 |
| *Argentina gombalana* | 0.143 | 3.337 |
| *Argentina gorokana* | 0.234 | 0.399 |
| *Argentina habbemana* | 0.092 | 6.121 |
| *Argentina hooglandii* | 0.155 | 1.896 |
| *Argentina indivisa* | 0.049 | 16.945 |
| *Argentina interrupta* | 0.174 | 0.443 |
| *Argentina irianensis* | 0.155 | 3.023 |
| *Argentina kinabaluensis* | 0.106 | 3.397 |
| *Argentina leuconota* | 0.123 | 3.127 |
| *Argentina lignosa* | 0.148 | 0.846 |
| *Argentina lineata* | 0.158 | 4.189 |
| *Argentina linilaciniata* | 0.106 | 3.397 |
| *Argentina luteopilosa* | 0.219 | 0.705 |
| *Argentina mangenii* | 0.060 | 15.405 |
| *Argentina micropetala* | 0.140 | 1.536 |
| *Argentina microphylla* | 0.313 | 1.509 |
| *Argentina millefoliolata* | 0.313 | 1.509 |
| *Argentina novoguineensis* | 0.129 | 4.997 |
| *Argentina papuana* | 0.090 | 7.426 |
| *Argentina parvula* | 0.140 | 1.536 |
| *Argentina peduncularis* | 0.213 | 2.364 |
| *Argentina phanerophlebia* | 0.152 | 0.432 |
| *Argentina polyphylla* | 0.112 | 6.733 |
| *Argentina pycnophylla* | 0.126 | 1.215 |
| *Argentina scorpionis* | 0.086 | 7.857 |
| *Argentina simulans* | 0.155 | 3.023 |
| *Argentina smithiana* | 0.119 | 1.125 |
| *Argentina stenophylla* | 0.392 | 0.414 |
| *Argentina sumatrana* | 0.126 | 1.215 |
| *Argentina taliensis* | 0.090 | 4.336 |
| *Argentina tapetodes* | 0.086 | 7.857 |
| *Argentina tatsienluensis* | 0.090 | 4.336 |
| *Argentina tristis* | 0.117 | 3.352 |
| *Argentina tugitakensis* | 0.112 | 6.733 |
| *Argentina turfosa* | 0.117 | 3.352 |
| *Argentina victorialis* | 0.091 | 4.692 |
| *Argentina vittata* | 0.062 | 13.073 |
| *Argentina wanimboi* | 0.196 | 2.470 |
| *Argentina wenchuensis* | 0.217 | 2.501 |
| *Argentina wilhelminensis* | 0.392 | 0.414 |
| *Argentina yonoweana* | 0.205 | 3.455 |
| *Potentilla acaulis* | 1.581 | 0.379 |
| *Potentilla adenotricha* | 3.451 | 0.007 |
| *Potentilla agrimonioides* | 1.887 | 0.194 |
| *Potentilla alba* | 0.095 | 8.806 |
| *Potentilla albiflora* | 1.466 | 0.117 |
| *Potentilla alchimilloides* | 0.759 | 0.137 |
| *Potentilla algida* | 0.819 | 0.767 |
| *Potentilla alpicola* | 0.662 | 0.952 |
| *Potentilla alsatica* | 0.511 | 0.883 |
| *Potentilla ambigens* | 1.261 | 0.638 |
| *Potentilla anachoretica* | 1.255 | 0.223 |
| *Potentilla ancistrifolia* | 0.197 | 1.124 |
| *Potentilla angelliae* | 0.983 | 0.313 |
| *Potentilla anglica* | 0.208 | 2.694 |
| *Potentilla angustiloba* | 0.685 | 0.097 |
| *Potentilla anjuica* | 1.418 | 0.200 |
| *Potentilla apennina* | 0.383 | 0.199 |
| *Potentilla aperta* | 2.372 | 0.045 |
| *Potentilla approximata* | 2.338 | 0.237 |
| *Potentilla arcadiensis* | 0.285 | 2.653 |
| *Potentilla arenosa* | 1.721 | 0.270 |
| *Potentilla argaea* | 1.912 | 0.036 |
| *Potentilla argentea* | 3.543 | 0.046 |
| *Potentilla argenteiformis* | 0.983 | 0.313 |
| *Potentilla argyrocoma* | 0.944 | 0.921 |
| *Potentilla argyroloma* | 1.176 | 0.583 |
| *Potentilla argyrophylla* | 1.214 | 0.641 |
| *Potentilla arizonica* | 3.400 | 0.069 |
| *Potentilla articulata* | 0.111 | 2.502 |
| *Potentilla aspegrenii* | 1.015 | 0.872 |
| *Potentilla asperrima* | 1.010 | 0.905 |
| *Potentilla assalemica* | 0.895 | 0.695 |
| *Potentilla astracanica* | 1.811 | 0.441 |
| *Potentilla asturica* | 1.665 | 0.125 |
| *Potentilla aucheriana* | 0.854 | 0.454 |
| *Potentilla aurea* | 0.886 | 1.058 |
| *Potentilla baileyi* | 0.764 | 0.801 |
| *Potentilla balansae* | 0.997 | 0.492 |
| *Potentilla basaltica* | 1.635 | 0.375 |
| *Potentilla beringii* | 2.428 | 0.236 |
| *Potentilla betonicifolia* | 3.996 | 0.073 |
| *Potentilla biennis* | 0.125 | 6.176 |
| *Potentilla biflora* | 0.111 | 2.502 |
| *Potentilla bipinnatifida* | 1.661 | 0.312 |
| *Potentilla bolanderi* | 0.925 | 0.698 |
| *Potentilla brachypetala* | 0.759 | 0.137 |
| *Potentilla brauneana* | 0.638 | 1.165 |
| *Potentilla brevifolia* | 1.627 | 0.230 |
| *Potentilla brunnescens* | 1.060 | 0.788 |
| *Potentilla buccoana* | 0.901 | 0.507 |
| *Potentilla bungei* | 0.830 | 0.765 |
| *Potentilla butkovii* | 1.634 | 0.363 |
| *Potentilla calabra* | 0.731 | 0.682 |
| *Potentilla californica* | 1.183 | 0.610 |
| *Potentilla caliginosa* | 1.168 | 0.473 |
| *Potentilla callida* | 2.372 | 0.045 |
| *Potentilla campestris* | 1.289 | 0.435 |
| *Potentilla canadensis* | 0.267 | 2.253 |
| *Potentilla candicans* | 1.997 | 0.070 |
| *Potentilla cappadocica* | 1.240 | 0.644 |
| *Potentilla carniolica* | 0.307 | 1.494 |
| *Potentilla caulescens* | 0.502 | 1.605 |
| *Potentilla centigrana* | 0.036 | 18.390 |
| *Potentilla chamaeleo* | 4.749 | 0.133 |
| *Potentilla chamissonis* | 2.261 | 0.210 |
| *Potentilla chinensis* | 1.254 | 0.475 |
| *Potentilla chionea* | 1.085 | 0.485 |
| *Potentilla chrysantha* | 1.581 | 0.379 |
| *Potentilla cinerea* | 0.649 | 1.358 |
| *Potentilla clandestina* | 0.261 | 0.073 |
| *Potentilla clevelandii* | 0.968 | 0.591 |
| *Potentilla clusiana* | 0.267 | 0.929 |
| *Potentilla collina* | 1.929 | 0.138 |
| *Potentilla concinna* | 0.905 | 0.602 |
| *Potentilla conferta* | 0.893 | 0.586 |
| *Potentilla congesta* | 1.135 | 0.453 |
| *Potentilla coriandrifolia* | 0.115 | 5.940 |
| *Potentilla coriandrifolia* var. *dumosa* | 0.189 | 1.902 |
| *Potentilla cottamii* | 1.257 | 0.366 |
| *Potentilla crantzii* | 0.947 | 0.958 |
| *Potentilla crassinervia* | 0.272 | 0.676 |
| *Potentilla crenulata* | 2.710 | 0.150 |
| *Potentilla crinita* | 2.176 | 0.234 |
| *Potentilla cristae* | 1.599 | 0.444 |
| *Potentilla cryptocaulis* | 0.786 | 0.727 |
| *Potentilla cryptophila* | 2.198 | 0.107 |
| *Potentilla cryptotaeniae* | 0.414 | 1.356 |
| *Potentilla curviseta* | 0.214 | 3.302 |
| *Potentilla darvazica* | 1.339 | 0.472 |
| *Potentilla daucifolia* | 1.852 | 0.155 |
| *Potentilla delavayi* | 1.506 | 0.313 |
| *Potentilla delphinensis* | 0.826 | 1.016 |
| *Potentilla demotica* | 0.940 | 0.612 |
| *Potentilla dentata* | 0.979 | 0.574 |
| *Potentilla deorum* | 0.400 | 0.641 |
| *Potentilla desertorum* | 0.559 | 1.420 |
| *Potentilla detommasii* | 0.903 | 0.849 |
| *Potentilla dickinsii* | 0.111 | 3.036 |
| *Potentilla dickinsii* var. *glabrata* | 0.197 | 1.124 |
| *Potentilla discolor* | 2.176 | 0.234 |
| *Potentilla divaricata* | 4.976 | 0.033 |
| *Potentilla divina* | 0.177 | 3.255 |
| *Potentilla doerfleri* | 0.383 | 0.199 |
| *Potentilla dombeyi* | 1.198 | 0.501 |
| *Potentilla doubjonneana* | 1.717 | 0.322 |
| *Potentilla douglasii* | 1.852 | 0.155 |
| *Potentilla drummondii* | 5.765 | 0.076 |
| *Potentilla durangensis* | 3.658 | 0.112 |
| *Potentilla effusa* | 2.064 | 0.121 |
| *Potentilla ehrenbergiana* | 2.515 | 0.053 |
| *Potentilla elatior* | 0.267 | 0.929 |
| *Potentilla elegans* | 0.907 | 0.086 |
| *Potentilla elvendensis* | 1.754 | 0.075 |
| *Potentilla erecta* | 0.191 | 3.553 |
| *Potentilla eriocarpa* | 0.088 | 9.079 |
| *Potentilla eversmanniana* | 2.812 | 0.093 |
| *Potentilla evestita* | 0.649 | 1.368 |
| *Potentilla exuta* | 1.116 | 0.440 |
| *Potentilla fedtschenkoana* | 3.390 | 0.103 |
| *Potentilla flabellata* | 1.717 | 0.322 |
| *Potentilla flabellifolia* | 1.180 | 0.218 |
| *Potentilla flagellaris* | 0.168 | 2.585 |
| *Potentilla forrestii* | 0.632 | 1.442 |
| *Potentilla fragarioides* | 1.793 | 0.213 |
| *Potentilla fragiformis* | 0.659 | 1.055 |
| *Potentilla freyniana* | 1.108 | 0.338 |
| *Potentilla frigida* | 1.243 | 0.602 |
| *Potentilla furcata* | 0.948 | 0.315 |
| *Potentilla gageodoensis* | 1.793 | 0.213 |
| *Potentilla gelida* | 0.854 | 0.454 |
| *Potentilla geranioides* | 0.948 | 0.315 |
| *Potentilla gerardiana* | 0.516 | 1.507 |
| *Potentilla glaucescens* | 0.923 | 0.783 |
| *Potentilla glaucophylla* | 1.465 | 0.434 |
| *Potentilla gobica* | 1.428 | 0.490 |
| *Potentilla goldmanii* | 1.484 | 0.127 |
| *Potentilla gordonii* | 0.564 | 1.334 |
| *Potentilla gracilis* | 2.064 | 0.121 |
| *Potentilla grammopetala* | 0.272 | 0.676 |
| *Potentilla grandiflora* | 1.194 | 0.641 |
| *Potentilla granulosa* | 0.608 | 1.460 |
| *Potentilla grayi* | 0.550 | 1.452 |
| *Potentilla griffithii* | 1.575 | 0.206 |
| *Potentilla grisea* | 1.371 | 0.259 |
| *Potentilla haynaldiana* | 0.214 | 3.302 |
| *Potentilla hebiichigo* | 0.168 | 2.585 |
| *Potentilla hemsleyana* | 0.280 | 0.513 |
| *Potentilla hendersonii* | 0.915 | 0.413 |
| *Potentilla heptaphylla* | 2.299 | 0.141 |
| *Potentilla heterosepala* | 3.451 | 0.007 |
| *Potentilla hickmanii* | 0.762 | 0.830 |
| *Potentilla hippiana* | 0.649 | 1.368 |
| *Potentilla hirta* | 1.243 | 0.602 |
| *Potentilla hispanica* | 1.015 | 0.606 |
| *Potentilla hispidula* | 2.091 | 0.076 |
| *Potentilla holmgrenii* | 5.476 | 0.010 |
| *Potentilla hololeuca* | 0.511 | 0.883 |
| *Potentilla hookeriana* | 3.367 | 0.017 |
| *Potentilla horrida* | 0.879 | 0.939 |
| *Potentilla howellii* | 1.554 | 0.377 |
| *Potentilla humifusa* | 1.811 | 0.441 |
| *Potentilla humillis* | 0.659 | 1.247 |
| *Potentilla hyparctica* | 2.468 | 0.105 |
| *Potentilla hypargyrea* | 0.327 | 0.824 |
| *Potentilla hypargyrea* var. *subpinnata* | 0.327 | 0.824 |
| *Potentilla ikonnikovii* | 1.656 | 0.432 |
| *Potentilla impolita* | 4.976 | 0.033 |
| *Potentilla incana* | 1.959 | 0.143 |
| *Potentilla indica* | 0.151 | 0.618 |
| *Potentilla intermedia* | 1.481 | 0.246 |
| *Potentilla iranica* | 1.111 | 0.537 |
| *Potentilla jaegeri* | 1.675 | 0.283 |
| *Potentilla jenissejensis* | 0.622 | 1.270 |
| *Potentilla jepsonii* | 0.965 | 0.760 |
| *Potentilla johanniniana* | 1.395 | 0.092 |
| *Potentilla johnstonii* | 1.734 | 0.139 |
| *Potentilla khanminczunii* | 1.339 | 0.472 |
| *Potentilla kingii* | 1.081 | 0.575 |
| *Potentilla kionaea* | 0.307 | 1.494 |
| *Potentilla kleiniana* | 1.085 | 0.485 |
| *Potentilla kotschyana* | 1.634 | 0.363 |
| *Potentilla kryloviana* | 0.743 | 1.214 |
| *Potentilla kurdica* | 0.647 | 1.338 |
| *Potentilla lancinata* | 1.154 | 0.521 |
| *Potentilla lasiodonta* | 3.996 | 0.073 |
| *Potentilla lazica* | 2.468 | 0.105 |
| *Potentilla leptopetala* | 0.723 | 1.192 |
| *Potentilla leschenaultiana* | 1.428 | 0.490 |
| *Potentilla leucopolitana* | 1.768 | 0.267 |
| *Potentilla libanotica* | 0.672 | 0.079 |
| *Potentilla lignipes* | 0.739 | 1.124 |
| *Potentilla limprichtii* | 5.936 | 0.042 |
| *Potentilla lindleyi* | 0.896 | 0.758 |
| *Potentilla lomakinii* | 1.702 | 0.382 |
| *Potentilla longibracteata* | 1.221 | 0.214 |
| *Potentilla longifolia* | 0.774 | 0.558 |
| *Potentilla longipes* | 3.543 | 0.046 |
| *Potentilla luteosericea* | 0.581 | 1.504 |
| *Potentilla lycopodioides* | 0.249 | 2.594 |
| *Potentilla lyngei* | 2.261 | 0.210 |
| *Potentilla macdonaldii* | 1.941 | 0.170 |
| *Potentilla macounii* | 0.648 | 1.431 |
| *Potentilla macrosepala* | 1.484 | 0.127 |
| *Potentilla mallota* | 0.886 | 0.438 |
| *Potentilla marinensis* | 2.597 | 0.269 |
| *Potentilla matsumurae* | 1.255 | 0.223 |
| *Potentilla maura* | 1.466 | 0.117 |
| *Potentilla megalantha* | 1.654 | 0.014 |
| *Potentilla mexiae* | 0.907 | 0.086 |
| *Potentilla meyeri* | 1.348 | 0.485 |
| *Potentilla micheneri* | 1.845 | 0.155 |
| *Potentilla micrantha* | 0.171 | 5.105 |
| *Potentilla millefolia* | 2.280 | 0.266 |
| *Potentilla mollissima* | 0.554 | 1.423 |
| *Potentilla monanthes* | 1.268 | 0.002 |
| *Potentilla mongolica* | 1.134 | 0.246 |
| *Potentilla montana* | 0.502 | 1.605 |
| *Potentilla montenegrina* | 5.936 | 0.042 |
| *Potentilla morefieldii* | 0.965 | 0.760 |
| *Potentilla muirii* | 0.809 | 1.049 |
| *Potentilla multicaulis* | 0.885 | 1.042 |
| *Potentilla multiceps* | 1.168 | 0.473 |
| *Potentilla multifida* | 0.822 | 1.097 |
| *Potentilla multifoliolata* | 0.477 | 1.512 |
| *Potentilla multijuga* | 1.337 | 0.389 |
| *Potentilla multisecta* | 0.712 | 1.261 |
| *Potentilla nana* | 3.658 | 0.112 |
| *Potentilla neglecta* | 1.008 | 0.034 |
| *Potentilla nepalensis* | 1.747 | 0.241 |
| *Potentilla nervosa* | 1.115 | 0.079 |
| *Potentilla nevadensis* | 1.768 | 0.267 |
| *Potentilla newberryi* | 0.685 | 0.097 |
| *Potentilla nitida* | 0.420 | 1.861 |
| *Potentilla nivalis* | 0.211 | 1.279 |
| *Potentilla nivea* | 1.395 | 0.092 |
| *Potentilla nordmanniana* | 0.961 | 0.912 |
| *Potentilla norvegica* | 1.481 | 0.246 |
| *Potentilla nubigena* | 0.548 | 1.438 |
| *Potentilla nuda* | 1.656 | 0.432 |
| *Potentilla nurensis* | 2.176 | 0.016 |
| *Potentilla olchonensis* | 1.336 | 0.462 |
| *Potentilla omeiensis* | 0.205 | 1.825 |
| *Potentilla omissa* | 1.336 | 0.462 |
| *Potentilla opizii* | 4.749 | 0.133 |
| *Potentilla osterhoutii* | 1.221 | 0.214 |
| *Potentilla ovina* | 1.180 | 0.218 |
| *Potentilla oweriniana* | 0.150 | 4.963 |
| *Potentilla pamirica* | 1.781 | 0.110 |
| *Potentilla pamiroalaica* | 1.599 | 0.444 |
| *Potentilla paniculata* | 0.249 | 2.594 |
| *Potentilla pannosa* | 0.583 | 1.382 |
| *Potentilla parryi* | 2.193 | 0.067 |
| *Potentilla patellifera* | 4.048 | 0.109 |
| *Potentilla pedata* | 1.702 | 0.382 |
| *Potentilla pedersenii* | 1.781 | 0.110 |
| *Potentilla pendula* | 0.902 | 0.813 |
| *Potentilla penniphylla* | 0.736 | 0.996 |
| *Potentilla pensylvanica* | 1.341 | 0.482 |
| *Potentilla persica* | 0.997 | 0.492 |
| *Potentilla peterae* | 0.889 | 0.529 |
| *Potentilla petraea* | 1.661 | 0.312 |
| *Potentilla petrovskyi* | 0.294 | 2.340 |
| *Potentilla pickeringii* | 1.554 | 0.377 |
| *Potentilla pimpinelloides* | 0.802 | 0.976 |
| *Potentilla pindicola* | 0.681 | 0.816 |
| *Potentilla pityocharis* | 1.049 | 0.631 |
| *Potentilla plattensis* | 0.795 | 0.435 |
| *Potentilla plumosa* | 2.176 | 0.016 |
| *Potentilla potaninii* | 1.238 | 0.507 |
| *Potentilla potaninii* var. *compsophylla* | 1.925 | 0.231 |
| *Potentilla praecox* | 0.513 | 1.830 |
| *Potentilla pseudosericea* | 1.997 | 0.070 |
| *Potentilla pseudosimulatrix* | 0.290 | 0.289 |
| *Potentilla pulchella* | 1.754 | 0.075 |
| *Potentilla pulcherrima* | 1.575 | 0.206 |
| *Potentilla pulvinaris* | 1.186 | 0.429 |
| *Potentilla pulviniformis* | 0.885 | 1.042 |
| *Potentilla purpurascens* | 1.428 | 0.367 |
| *Potentilla purpurea* | 0.205 | 1.825 |
| *Potentilla pusilla* | 2.198 | 0.107 |
| *Potentilla pyrenaica* | 0.554 | 1.423 |
| *Potentilla radiata* | 0.710 | 1.037 |
| *Potentilla ranunculoides* | 1.506 | 0.313 |
| *Potentilla recta* | 2.081 | 0.297 |
| *Potentilla recta* subsp. *obscura* | 2.081 | 0.297 |
| *Potentilla reptans* | 0.208 | 2.694 |
| *Potentilla reuteri* | 0.830 | 0.765 |
| *Potentilla rhenana* | 1.098 | 0.765 |
| *Potentilla rhyolitica* | 1.008 | 0.034 |
| *Potentilla rhypara* | 0.458 | 1.591 |
| *Potentilla richardii* | 0.354 | 2.241 |
| *Potentilla rigidula* | 1.214 | 0.641 |
| *Potentilla rigoana* | 0.724 | 0.493 |
| *Potentilla rimicola* | 1.465 | 0.434 |
| *Potentilla riparia* | 0.247 | 1.482 |
| *Potentilla rivalis* | 1.912 | 0.036 |
| *Potentilla robbinsiana* | 1.257 | 0.366 |
| *Potentilla rosulifera* | 0.581 | 0.866 |
| *Potentilla rubella* | 1.697 | 0.050 |
| *Potentilla rubricaulis* | 1.484 | 0.448 |
| *Potentilla rudolfii* | 3.686 | 0.104 |
| *Potentilla rupifraga* | 5.765 | 0.076 |
| *Potentilla rupincola* | 1.337 | 0.389 |
| *Potentilla ruprechtii* | 1.887 | 0.194 |
| *Potentilla rydbergii* | 1.076 | 0.586 |
| *Potentilla sabulosa* | 2.091 | 0.076 |
| *Potentilla salsa* | 1.959 | 0.143 |
| *Potentilla sanguisorba* | 0.731 | 0.682 |
| *Potentilla santolinoides* | 1.234 | 0.385 |
| *Potentilla saposhnikovii* | 1.665 | 0.125 |
| *Potentilla saundersiana* | 0.712 | 0.965 |
| *Potentilla saxifraga* | 0.672 | 0.079 |
| *Potentilla saxosa* | 1.276 | 0.614 |
| *Potentilla schmakovii* | 0.867 | 0.970 |
| *Potentilla schrenkiana* | 0.710 | 1.037 |
| *Potentilla schurii* | 1.245 | 0.566 |
| *Potentilla sergievskajae* | 1.129 | 0.632 |
| *Potentilla sericata* | 2.193 | 0.067 |
| *Potentilla sericea* | 1.015 | 0.872 |
| *Potentilla sericoleuca* | 1.289 | 0.435 |
| *Potentilla serrata* | 0.616 | 1.340 |
| *Potentilla setosa* | 0.683 | 1.041 |
| *Potentilla shockleyi* | 0.530 | 1.092 |
| *Potentilla sierrae-blancae* | 1.941 | 0.170 |
| *Potentilla sikkimensis* | 0.220 | 1.500 |
| *Potentilla silesiaca* | 0.547 | 1.747 |
| *Potentilla simplex* | 0.267 | 2.253 |
| *Potentilla simulatrix* | 0.280 | 0.513 |
| *Potentilla sinonivea* | 3.400 | 0.069 |
| *Potentilla sischanensis* | 5.476 | 0.010 |
| *Potentilla soongorica* | 1.654 | 0.014 |
| *Potentilla speciosa* | 0.211 | 1.279 |
| *Potentilla sphenophylla* | 1.055 | 0.659 |
| *Potentilla spodiochlora* | 1.289 | 0.567 |
| *Potentilla squamosa* | 0.290 | 0.289 |
| *Potentilla staminea* | 0.730 | 0.878 |
| *Potentilla sterilis* | 0.177 | 3.255 |
| *Potentilla stipularis* | 0.745 | 0.898 |
| *Potentilla stolonifera* | 0.682 | 0.359 |
| *Potentilla strigosa* | 1.341 | 0.482 |
| *Potentilla suavis* | 0.220 | 1.500 |
| *Potentilla subarenaria* | 1.673 | 0.244 |
| *Potentilla subdigitata* | 1.116 | 0.440 |
| *Potentilla subgorodkovii* | 1.258 | 0.407 |
| *Potentilla subjuga* | 0.624 | 1.492 |
| *Potentilla subpalmata* | 0.570 | 1.621 |
| *Potentilla subvahliana* | 2.515 | 0.053 |
| *Potentilla subviscosa* | 1.032 | 0.742 |
| *Potentilla supina* | 1.697 | 0.050 |
| *Potentilla szovitsii* | 1.176 | 0.583 |
| *Potentilla tabernaemontani* | 1.198 | 0.501 |
| *Potentilla tanacetifolia* | 1.254 | 0.475 |
| *Potentilla taurica* | 0.872 | 1.022 |
| *Potentilla tenuis* | 0.261 | 0.073 |
| *Potentilla tephroleuca* | 0.923 | 0.783 |
| *Potentilla tericholica* | 1.194 | 0.641 |
| *Potentilla tetrandra* | 0.126 | 2.943 |
| *Potentilla thurberi* | 0.901 | 0.507 |
| *Potentilla thuringiaca* | 2.428 | 0.236 |
| *Potentilla thyrsiflora* | 2.478 | 0.209 |
| *Potentilla tilingii* | 1.276 | 0.614 |
| *Potentilla tobolensis* | 0.638 | 1.165 |
| *Potentilla togasii* | 0.812 | 0.742 |
| *Potentilla tollii* | 0.647 | 1.423 |
| *Potentilla tommasiniana* | 2.055 | 0.288 |
| *Potentilla tornezyana* | 0.816 | 0.826 |
| *Potentilla townsendii* | 1.268 | 0.002 |
| *Potentilla toyamensis* | 0.812 | 0.742 |
| *Potentilla truncata* | 1.995 | 0.303 |
| *Potentilla tschimganica* | 1.115 | 0.079 |
| *Potentilla tschukotica* | 3.367 | 0.017 |
| *Potentilla tucumanensis* | 0.946 | 0.325 |
| *Potentilla tularensis* | 4.048 | 0.109 |
| *Potentilla turczaninowiana* | 1.172 | 0.543 |
| *Potentilla turgaica* | 1.721 | 0.270 |
| *Potentilla tweedyi* | 1.845 | 0.155 |
| *Potentilla uliginosa* | 0.889 | 0.529 |
| *Potentilla umbrosa* | 1.172 | 0.543 |
| *Potentilla unguiculata* | 2.296 | 0.061 |
| *Potentilla uniflora* | 1.258 | 0.407 |
| *Potentilla utahensis* | 0.915 | 0.413 |
| *Potentilla vahliana* | 0.930 | 1.022 |
| *Potentilla valderia* | 0.122 | 6.338 |
| *Potentilla verna* | 3.470 | 0.173 |
| *Potentilla verticillaris* | 0.879 | 0.939 |
| *Potentilla villosa* | 1.627 | 0.230 |
| *Potentilla virgata* | 0.911 | 0.957 |
| *Potentilla visianii* | 0.724 | 0.493 |
| *Potentilla volgarica* | 0.414 | 2.227 |
| *Potentilla vulcanicola* | 0.722 | 1.056 |
| *Potentilla vvedenskyi* | 0.733 | 1.230 |
| *Potentilla webberi* | 2.296 | 0.061 |
| *Potentilla wheeleri* | 0.946 | 0.325 |
| *Potentilla wilderae* | 1.253 | 0.536 |
| *Potentilla wimanniana* | 1.134 | 0.246 |
| *Potentilla wrangelii* | 0.215 | 2.847 |
| *Potentilla xizangensis* | 0.860 | 0.809 |

**References**

1. Westerhold T, Marwan N, Drury AJ, Liebrand D, Agnini C, Anagnostou E, et al. An astronomically dated record of Earth's climate and its predictability over the last 66 million years. Science. 2020;369(6509):1383–1387.

2. Li CL, Ikeda H, Ohba H: *Potentilla* Linnaeus. In: Wu ZY, Raven PH, Hong DY, editors. Flora of China (vol. 9). Beijing: Science Press; 2003. pp.291–327.

3. Ertter B, Elven R, Reveal JL, Murray DF: *Potentilla* Linnaeus. In: Flora of North America Editorial Committee, editor. Flora of North America (vol. 9). Oxford: Oxford University Press; 2014. pp.121–218.

4. Ertter B, Reveal JL: *Ivesia* Torrey and A. Gray, *Horkelia* Chamisso and Schlechtendal, *Horkeliella* Rydberg, *Duchesnea* Smith. In: Flora of North America Editorial Committee, editor. Flora of North America (vol. 9). Oxford: Oxford University Press; 2014. pp.219–274.

5. Ball PW, Pawlowski B, Walters SM: *Potentilla* L. In: Tutin TG, Heywood VH, Burges NA, Moore DM, Valentine DH, Walters SM, et al., editors. Flora Europaea (vol. 2). Cambridge: Cambridge University Press; 1968. pp.36–47.

6. Juzepczuk SV: *Potentilla* L. In: Komarov VL, editor. Flora URSS (vol. 10). Moscow & Leningrad: Izdatel’stvo Akademii Nauk SSSR; 1941. pp.78–223.

7. Wolf T. Monographie der Gattung *Potentilla*. Bibliotheca Botanica. 1908;16(71):1–715.

8. Dobeš C, Paule J. A comprehensive chloroplast DNA-based phylogeny of the genus *Potentilla* (Rosaceae): implications for its geographic origin, phylogeography and generic circumscription. Mol Phylogen Evol. 2010;56(1):156–175.

9. Iatroú G. A new species of *Potentilla* (Rosaceae) from Peloponnesus, Greece. Candollea. 1985;40(1):121–128.

10. Eriksson T, Lundberg M, Töpel M, Östensson P, Smedmark JEE. *Sibbaldia*: a molecular phylogenetic study of a remarkably polyphyletic genus in Rosaceae. Plant Syst Evol. 2015;301(1):171–184.

11. Feng T, Moore MJ, Yan MH, Sun YX, Zhang HJ, Meng AP, et al. Phylogenetic study of the tribe Potentilleae (Rosaceae), with further insight into the disintegration of *Sibbaldia*. J Syst Evol. 2017;55(3):177–191.

12. Töpel M, Lundberg M, Eriksson T, Eriksen B. Molecular data and ploidal levels indicate several putative allopolyploidization events in the genus *Potentilla* (Rosaceae). PLoS currents. 2011;3:RRN1237.

13. Persson N, Toresen I, Andersen HL, Smedmark JEE, Eriksson T. Detecting destabilizing species in the phylogenetic backbone of *Potentilla* (Rosaceae) using low-copy nuclear markers. Aob Plants. 2020;12(3):plaa017.

14. Soják J. *Argentina* Hill, a genus distinct from *Potentilla* (Rosaceae). Thaiszia - J Bot. 2010;20:91–97.

15. Koski MH, Ashman TL. Macroevolutionary patterns of ultraviolet floral pigmentation explained by geography and associated bioclimatic factors. New Phytol. 2016;211(2):708–718.

16. Feng T, Moore MJ, Sun Y, Meng A, Chu H, Li J, et al. A new species of *Argentina* (Rosaceae, Potentilleae) from Southeast Tibet, with reference to the taxonomic status of the genus. Plant Syst Evol. 2015;301(3):911–921.

17. Tong YH, Xia NH. New Combinations for Chinese *Argentina* Hill (Rosaceae). JTrop & Subtrop Bot. 2016;24(4):426–428.

18. Polozhij AV, Malyschev LI: Rosaceae. In: Malyschev LI, editor. Flora of Siberia (vol. 8). Enfield (NH), USA & Plymouth, UK: Science Publishers, Inc.; 2004. pp.32–78.

19. Soják J. Nomenklatorische Anmerkungen zur Gattung *Potentilla*. Folia Geobot Phytotax. 1969;4:205–209.

20. Gregor T. *Potentilla alsatica* T. Gregor, ein Fingerkraut der *Potentilla-collina*-Gruppe aus der südlichen Oberrheinebene. Bauhinia. 2004;18:5–21.

21. Kurtto A, Eriksson T. Atlas Florae Europaeae notes. 15. Generic delimitation and nomenclatural adjustments in Potentilleae (Rosaceae). Ann Bot Fenn. 2003;40:135–141.

22. Soják J. New taxa and nomenclatural combinations in *Potentilla* L. (Rosaceae) Notes on *Potentilla* XXII. Feddes Repertorium. 2006;117:486–500.

23. Faghir MB, Attar F, Sojak J. *Potentilla radiata* Lehm. and *Potentilla balansae* Sojak. two new record species for the Flora of Iran. Journal of Taxonomy and Biosystematics. 2010;2:39–46.

24. Ma YZ, Li ZH, Wang X, Shang BL, Wu GL, Wang YJ. Phylogeography of the genus *Dasiphora* (Rosaceae) in the Qinghai-Tibetan Plateau: divergence blurred by expansion. Biol J Linn Soc. 2014;111(4):777–788.

25. Soják J. Taxonomische und phytogeographische Anmerkungen zur Gattung *Potentilla*. Preslia. 1970;42:70–81.

26. Landrein S, Borosova R, osborne J, Shah M, Rajput MTM, Tahir SS, et al: Rosaceae (I)-Potentilleae & Roseae. In: Ali SI, Qaiser M, editors. Flora of Pakistan (vol. 216); 2009. pp.14–64.

27. Mozaffarian V. New species and new records from Gilan Province, Iran. Iran J Bot. 2016;22:112–120.

28. Baasanmunkh S, Urgamal M, Oyuntsetseg B, Grabovskaya-Borodina A, Oyundelger K, Tsegmed Z, et al. Updated checklist of vascular plants endemic to Mongolia. Diversity-Basel. 2021;13(12): 619.

29. Faghir MB, Attar F, Farazmand A, Kazempour Osaloo S. Phylogeny of the genus *Potentilla* (Rosaceae) in Iran based on nrDNA ITS and cpDNA *trn*L-F sequences with a focus on leaf and style characters' evolution. Turk J Bot. 2014;38(3):417–429.

30. Erst AS, Nikulin AY, Nikulin VY, Ebel AL, Zibzeev EV, Sharples MT, et al. Distribution analysis, updated checklist, and DNA barcodes of the endemic vascular flora of the Altai mountains, a Siberian biodiversity hotspot. Syst Biodivers. 2022;20(1):2049391.

31. Soják J. A New Arctic *Potentilla* (Rosaceae). Willdenowia. 1985;30:167–169.

32. Kechaykin A, Kutsev M. Notes on *Potentilla* L. (Rosaceae) from the Altai. 2. New species from South Siberia and West Mongolia. Feddes Repertorium. 2015;126:73–76

33. Castagnaro A, Ricci JD, Arias M, Albornoz R. A new southern hemisphere species of *Potentilla* (Rosaceae). Novon. 1998;8(4):333–336.

34. Schanzer IA, Fedorova AV, Shelepova OV, Suleymanova GF. Molecular phylogeny and phylogeography of *Potentilla multifida* L. agg. (Rosaceae) in northern Eurasia with special focus on two rare and critically endangered endemic species, *P. volgarica* and *P. eversmanniana*. Plants-Basel. 2020;9(12):1798.

35. Heo KI, Lee S, Kim Y, Park J, Lee ST. Taxonomic studies of the tribe Potentilleae (Rosaceae) in Korea. Korean J Pl Taxon. 2019;49(1):28–69.

36. Naruhashi N: Rosoideae. In: Iwatsuki K, Boufford DE, Ohba H, editors. Flora of Japan (vol. IIb). Tokyo: Kodansha; 2003. pp.193–206.

37. Töpel M, Antonelli A, Yesson C, Eriksen B. Past climate change and plant evolution in western North America: a case study in Rosaceae. Plos One. 2012;7(12):e50358.

38. Mosyakin SL, Ertter B, Shiyan NM. New combinations in *Potentilla* for taxa originally validated or treated in *Ivesia*, *Horkelia*, and *Horkeliella* (Rosaceae: Potentilleae). Phytotaxa. 2020;474(3):261–271.
